# ALPaCA: Adapting Llama for Pathology Context Analysis to enable slide-level question answering

**DOI:** 10.1038/s41467-026-76372-z

**Published:** 2026-08-20

**Authors:** Zeyu Gao, Kai He, Weiheng Su, Xiaobo Pang, Ines P. Machado, Mercedes Jimenez-Linan, Brian Rous, Chunbao Wang, Chengzu Li, William McGough, Shangqi Gao, Di Zhang, Tieliang Gong, Ming Y. Lu, Faisal Mahmood, Mengling Feng, Chen Li, Mireia Crispin-Ortuzar

**Affiliations:** 1 Department of Oncology, University of Cambridge, Cambridge, UK; 2 CRUK Cambridge Centre, University of Cambridge, Cambridge, UK; 3 Saw Swee Hock School of Public Health, National University of Singapore, Singapore, Singapore; 4 School of Computer Science and Technology, Xi’an Jiaotong University, Xi’an, China; 5 Department of Pathology, Cambridge University Hospitals, Cambridge, UK; 6 Department of Pathology, First Affiliated Hospital of Xi’an Jiaotong University, Xi’an, China; 7 Language Technology Lab, University of Cambridge, Cambridge, UK; 8 Department of Pathology, Harvard Medical School, Boston, USA

**Keywords:** Cancer imaging, Computational models, Machine learning, Pathology, Cancer screening

## Abstract

Large Vision Language Models (LVLMs) are increasingly used in computational pathology for image classification, description generation, question answering and interactive diagnostics. However, most pathology LVLMs analyse small regions of interest rather than pyramidal, gigapixel-scale whole-slide images (WSIs), limiting their use for tasks requiring whole-slide assessment across sub-regions and magnification levels. Here, we present ALPaCA (Adapting Llama for Pathology Context Analysis), a slide-level LVLM framework for WSI question answering across diverse cancer types and tissue sites. ALPaCA is trained using 35,913 WSIs with curated descriptions and 341,051 question-answer pairs from TCGA and GTEx. It combines a LongFormer vision-text adaptor with a Gaussian mixture model-based prototyping adaptor and Llama3.1. ALPaCA exceeds 90% accuracy on internal close-ended benchmarks and maintains 77–82% accuracy on independent external cohorts. Expert pathologist evaluation of open-ended responses supports its slide-level reasoning capability. Additionally, ALPaCA can be fine-tuned on organ- or disease-specific datasets, supporting specialised pathology question answering.

## Abstract (web summary)

Large Vision Language Models (LVLMs) have revolutionised computational pathology, but still struggle with the analysis of pyramidal, gigapixel-scale whole-slide images (WSIs). Here, the authors introduce ALPaCA (Adapting Llama for Pathology Context Analysis), a general-purpose slide-level LVLM designed for WSI analysis and question answering across a broad range of cancer types and tissue sites.

## Introduction

In recent years, computational pathology has evolved rapidly, largely due to progress in deep learning and digital scanning technology^1^. These breakthroughs have made it possible to automatically analyse whole-slide images (WSIs), supporting tasks like tumour morphological classification^2–5^, biomarker prediction^6–8^, expression prediction^9–11^, treatment response^12–14^ and prognostic analysis^15–17^. Notably, the rise of foundational models for pathology—trained on extensive collections of unlabelled WSIs—has introduced task-agnostic model backbones capable of learning broad and generalisable representations^18–22^. This innovation substantially enhances performance and label efficiency across diverse computational pathology tasks. However, most of these models disregard textual pathology knowledge and still rely on predefined tasks that require trainable heads for downstream applications, limiting their capacity for interactive reasoning and practical diagnostic use.

The rise of large language models (LLMs)^23–25^ has reshaped numerous domains of artificial intelligence by achieving unprecedented levels of language understanding and generation. Building on this foundation, Large Vision-Language Models (LVLMs)^26–29^ have emerged as multimodal extensions of LLMs, integrating visual information with textual reasoning to enable tasks such as zero-shot image classification, captioning, question answering, and interactive reasoning. Previous works^30–32^ have demonstrated the potential of LVLMs in medical imaging diagnosis and research. Among them, pathology-focused LVLMs^33–36^ are advancing an emerging direction in computational pathology, enabling pathology descriptions, interactive diagnostics, and even follow-up immunohistochemistry (IHC) recommendations for H&E-stained pathology images. Fundamentally, these LVLMs follow a similar framework: pathology image embeddings, extracted using a pre-trained frozen domain-specific encoder, are converted into LLM-recognisable tokens through a simple linear adaptor and concatenated with text tokens for LLM fine-tuning and inference. However, this framework is restricted to region-of-interest (ROI) analysis, focusing on small, predefined areas in isolation, and lacks the capability for comprehensive slide-level analysis. In real-world clinical practice, pathological diagnostics often demand reasoning across entire slides, integrating information from multiple sub-regions and magnification levels. This presents significant challenges for LVLMs that would need adaptors designed to handle the scale, complexity, and hierarchical structure of WSIs efficiently. While recent efforts have explored slide-level vision-language models, most lack integration with powerful LLMs and focus on generating simple slide descriptions^37–39^ or support tumour-focused question-answering (QA)^40,41^.

Here, we present ALPaCA (Adapting Llama for Pathology Context Analysis), a general-purpose LVLM designed for WSI QA across a broad range of cancer types and tissue sites. ALPaCA leverages frozen CONCH^42^, a vision encoder pre-trained on 1.17 million pathology image-caption pairs, to extract hierarchical patch embeddings. To enable comprehensive WSI analysis, ALPaCA incorporates vision-only and vision-text interactive slide-level adaptors for compressing patch embeddings into WSI tokens and integrating them with the publicly available Llama3.1-8B LLM. ALPaCA is trained sequentially on general slide-caption and slide-QA datasets curated from The Cancer Genome Atlas (TCGA) and The Genotype-Tissue Expression (GTEx), with each slide paired to a pathology report or note. We evaluate ALPaCA across diverse diagnostic types and tissue sites, benchmark it against general-purpose and pathology-specific LVLMs, assess its performance on two privately curated clinical cohorts, and further examine whether disease-specific fine-tuning supports specialised slide-level pathology QA tasks.

## Results

### A slide-level large vision language model for pathology question answering

**Fig. 1:**
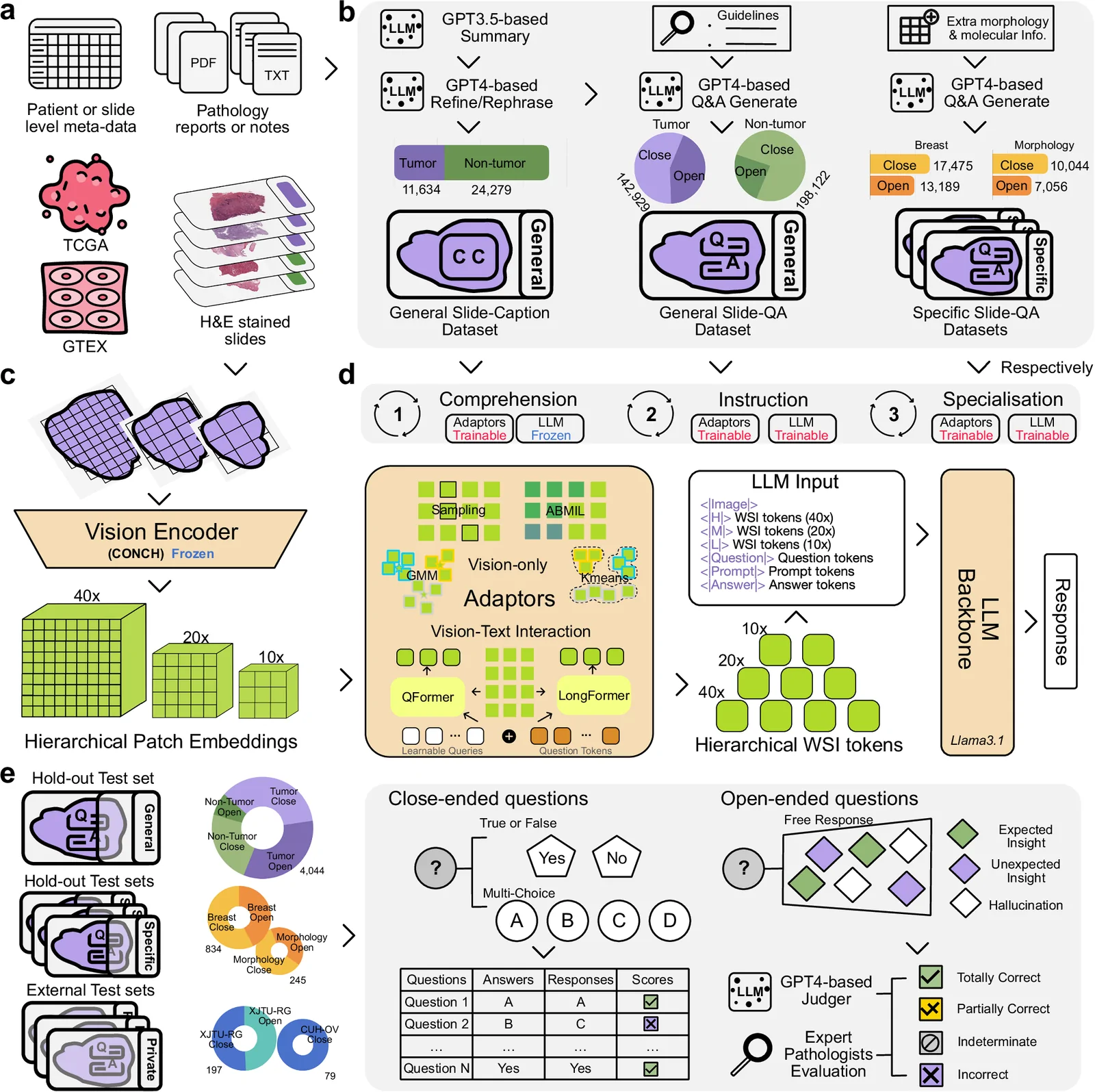
Study overview. **a** Source data from TCGA (tumour) and GTEx (non-tumour), including metadata, pathology reports/notes, and digitalised H& E-stained slides. **b** Dataset curation. Pathology reports were summarised with GPT-3.5, and slide descriptions were generated by GPT-4o using summaries/notes, and metadata to form the General Slide-Caption dataset. Question-answer pairs were created by GPT-4o with pathologist guidelines for the General Slide-QA dataset. Moreover, incorporating extra morphological and molecular information for a subset of slides, the Specific Slide-QA datasets were curated. **c** Hierarchical patch embeddings were extracted by the CONCH encoder at different magnifications. **d** ALPaCA overview. Patch embeddings were transformed into WSI tokens by various adaptors and combined with text tokens in a structured format as input for the LLM (Llama3.1-8B). Training proceeded in three stages---Comprehension, Instruction, and Specialisation---using curated datasets in sequence. **e** ALPaCA evaluation. Performance was assessed with hold-out and external test sets for general and specific QA. Close-ended Q& A accuracy was calculated directly, while open-ended Q& A was evaluated with a GPT-4o-based judger and expert pathologists. Source data are provided as a Source Data file.

We propose ALPaCA, a comprehensive framework for integrating image-level foundation models with powerful LLMs for pathology slide analysis, while also establishing a systematic approach for slide-level QA evaluation (Fig. 1). To develop a slide-level LVLM capable of addressing both general and specific pathology QA tasks, we introduced three key innovations. Firstly, we curated two large-scale datasets, General Slide-Caption and General Slide-QA, sourced from TCGA and GTEx, comprising 35,913 WSIs with corresponding descriptions and 341,051 Q&A pairs (Fig. 1a, b and Supplementary Fig. 1). Secondly, we trained multiple ALPaCA variants with various slide-level adaptors to effectively integrate the pathology foundation model (CONCH) with the Llama3.1 LLM using a sequential training strategy on the curated datasets (Fig. 1c, d). In particular, six different slide-level adaptors were developed to explore optimal integration approaches, including four vision-only adaptors (i.e., Sampling, Attention-Based Multi-Instance Learning (ABMIL), KMeans, and Gaussian Mixture Model (GMM)) and two vision-text interactive adaptors (QFormer and LongFormer). The best-performing variant, ALPaCA-Hybrid, incorporates LongFormer and GMM, where LongFormer specialises in extracting question-specific detailed local visual representations, while GMM effectively captures global visual information for addressing coarse-grained questions. Finally, we fine-tuned ALPaCA-Hybrid on two disease-specific QA datasets, each tailored to address in-depth morphological and molecular questions related to the corresponding disease type.

To assess ALPaCA variants (Fig. 1e), we allocated a hold-out test set of slides spanning all diagnostic types and tissue sites in the curated datasets, with patient-level separation for TCGA and case-level separation for GTEx. This set was excluded from all training stages to ensure unbiased evaluation. General pathology QA performance was then assessed using 4044 slide-level Q&A pairs across all ALPaCA variants and benchmarked against four representative LVLMs (open-source and commercial) as well as a pathology-specific slide-level LVLM (SlideChat^41^). Both automated and expert pathologist assessments were employed to ensure a comprehensive and unbiased evaluation (Methods: Evaluation). We further curated two private external cohorts to evaluate the real-world generalisability of ALPaCA-Hybrid, using the same baseline LVLMs allowed under the respective data-use constraints. Finally, two specialised pathology QA tasks were assessed using ALPaCA-Hybrid, the best-performing variant identified in our general evaluations.

Beyond these main evaluations, we conducted additional analyses to further characterise ALPaCA’s behaviour. Specifically, we tested alternative pathology foundation model encoders (UNI^19^, MUSK^43^, PathGen^44^, Supplementary Note 1: Patch encoders) and included text-only and random-image baselines (Supplementary Note 2: Baseline controls) to assess the contribution of visual features. We also visualised spatial attention patterns (Supplementary Note 3: Visualisation) and compared performance between low-power and high-power question types (Supplementary Note 4: Low- vs high-power question analysis), corresponding to questions answerable from low-magnification cues only and those that additionally require high-magnification detail.

### Performance on close-ended questions

**Fig. 2:**
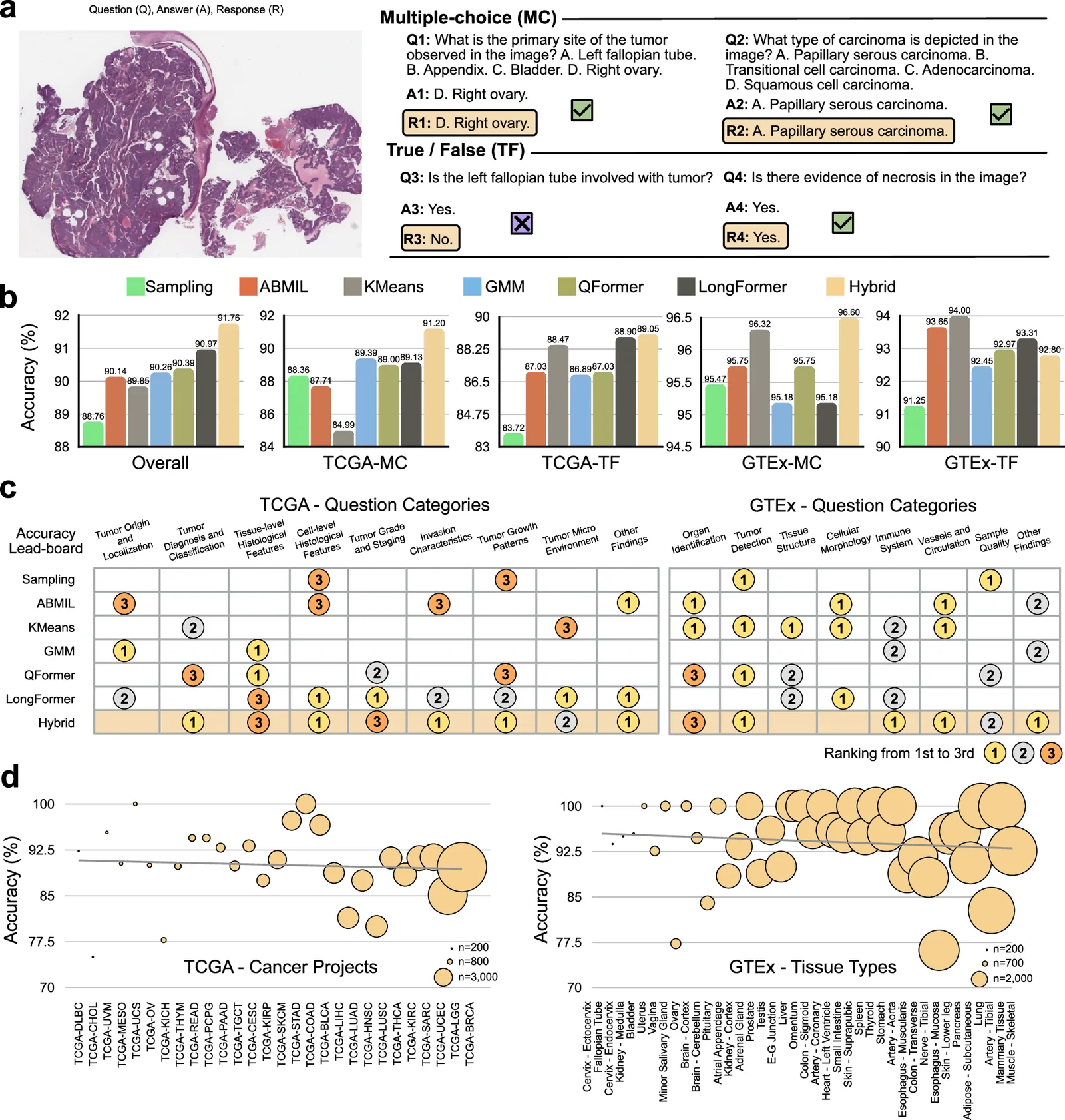
ALPaCA performance on close-ended questions. **a** An example case of an ovarian tumour slide with corresponding multiple-choice (MC) and True/False (TF) questions, ground-truth answers, and ALPaCA-Hybrid responses. **b** Overall accuracy for different ALPaCA variants with various adaptors (*n *= 2403 questions), including breakdowns for tumour-related multiple-choice (TCGA; *n *= 773 questions), tumour-related True/False (TCGA; *n *= 694 questions), non-tumour-related multiple-choice (GTEx; *n *= 353 questions) and non-tumour-related True/False (GTEx; *n *= 583 questions). **c** Performance lead-board of different ALPaCA variants across various question categories for TCGA (*n *= 1467 questions) and GTEx (*n *= 936 questions), respectively. **d** Performance of ALPaCA-Hybrid across cancer projects in TCGA (*n* = 1467 questions) and tissue sites in GTEx (*n *= 936 questions), along with the trend line of accuracy. Bubble size represents the number of training Q& A instructions (n) from each cancer project or tissue site, arranged from smallest to largest along the x-axis. Source data are provided as a Source Data file.

We first evaluated all ALPaCA variants on the close-ended general pathology QA task, which included True/False and Multiple-Choice questions (Fig. 2a) across 32 TCGA cancer projects and 40 GTEx tissue sites, with 1467 questions covering 9 slide examination categories for tumour cases and 936 questions covering 8 histopathological analysis categories for non-tumour cases. Except for the basic Sampling and KMeans variants, all models achieved over 90% accuracy, with ALPaCA-Hybrid performing best overall at 91.76% (Fig. 2b). It also achieved the highest accuracy across tumour-related multiple-choice questions (91.20%), tumour-related True/False questions (89.05%), and non-tumour multiple-choice questions (96.60%), while showing slightly weaker performance on non-tumour True/False questions (92.80%).

Analysis by question category (Fig. 2c) showed that most non-tumour tasks were handled consistently well across ALPaCA variants. For tumour-related tasks, ALPaCA-Hybrid and ALPaCA-LongFormer performed best, especially in categories requiring both global and region-level reasoning. Across all variants, the most challenging categories were tumour grading and staging, invasion characteristics, and tumour micro-environment (Supplementary Fig. 2), reflecting their dependence on fine-grained regional cues and multi-region information integration. Vision-text interactive adaptors (LongFormer, Hybrid) were therefore more effective, whereas QFormer’s heavier BERT backbone likely required more extensive pretraining than was available.

We further examined ALPaCA-Hybrid across cancer projects and tissue sites to assess potential dataset-dependent biases (Fig. 2d). Performance remained robust: 24/28 cancer projects and 36/40 tissue sites exceeded 85% accuracy, and even the lowest-performing subsets (TCGA-CHOL and Oesophagus-Mucosa) remained above 75%. Variability did not correlate with training data size but rather with question difficulty. For example, errors in TCGA-CHOL predominantly involved staging and invasion, which pathologists confirmed were indeterminate on the slide alone; a subtype-classification error (cholangiocarcinoma vs hepatocellular carcinoma) was attributed to atypical histologic appearance. In the Oesophagus-Mucosa subset, most errors were traced to a single GTEx case where the original pathology note was incorrect; expert review confirmed mucosa was present despite the report stating otherwise.

### Automatic and pathologist evaluation of open-ended questions

**Fig. 3:**
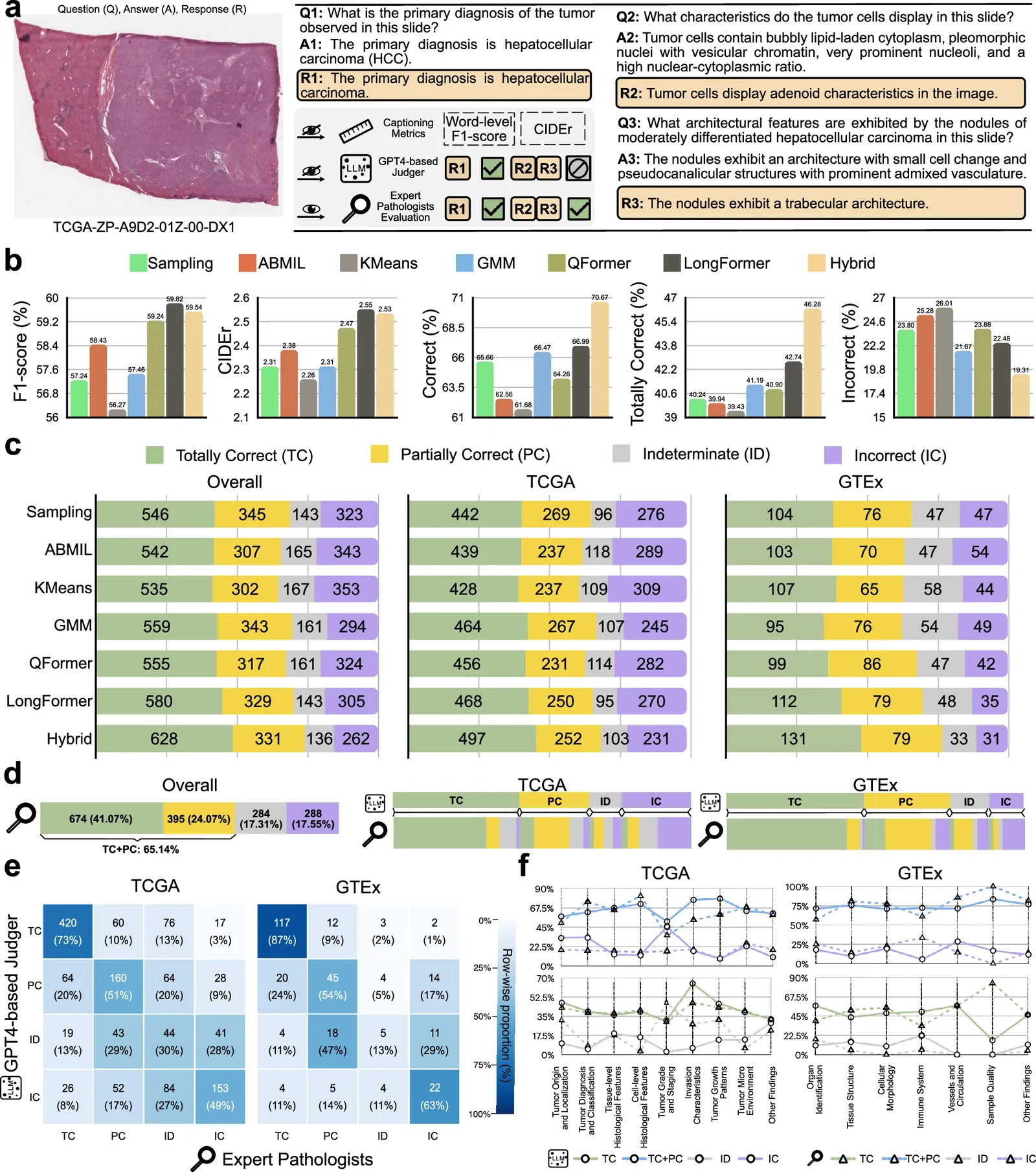
ALPaCA performance on open-ended questions. **a** An example case of a liver tumour slide with corresponding open-ended questions, ground-truth answers, and ALPaCA-Hybrid responses, evaluated through three different assessment methods. **b** Overall performance of different ALPaCA variants with various adaptors, measured by word-level F1-score, CIDEr, and correctness rates from the GPT-4o-based judger on answerable questions (*n *= 1357 questions). **c** Detailed quantitative assessment results from the GPT-4o-based judger for TCGA (*n *= 1083 questions) and GTEx (*n *= 274 questions). **d** Overall performance of ALPaCA-Hybrid evaluated by expert pathologists across the full open-ended test set (*n *= 1641 questions), with separate results for TCGA (*n *= 1351 questions) and GTEx (*n *= 290 questions). Expert adjustments to GPT-4o-based judger assessments are also shown. **e** Comparison of expert pathologists' assessments and GPT-4o-based judger evaluations across four evaluation categories for the full open-ended test set, including TCGA (*n *= 1351 questions) and GTEx (*n *= 290 questions). **f** Comparison of expert pathologists' assessments and GPT-4o-based judger evaluations across question categories for the full open-ended test set, including TCGA (*n *= 1351 questions) and GTEx (*n *= 290 questions), respectively. Source data are provided as a Source Data file.

Beyond close-ended questions, we evaluated ALPaCA’s performance on the open-ended general pathology QA task, comprising 1641 questions across tumour and non-tumour cases. This task better simulates real-world pathology diagnostics, where no predefined candidate options or even standard correct answers exist, making it crucial to evaluate whether ALPaCA can generate reasonable and relevant responses to the given questions and target slides. Given the challenges of evaluating open-ended QA, where correctness cannot be directly determined as in close-ended QA, we designed a comprehensive evaluation process (Fig. 3a) that compares automated and expert assessments across all 1641 questions. For quantitative reporting, we focus on the 1357 questions that expert pathologists judged answerable from the corresponding diagnostic slides.

First, we computed word-level F1 and CIDEr scores to obtain an initial estimate of response quality. ALPaCA variants with trainable adaptors outperformed fixed token-selection methods, with ALPaCA-LongFormer achieving the highest scores (59.82% F1, 2.55 CIDEr; Fig. 3b). These gains largely reflect improved aggregation of patch embeddings. However, because these lexical metrics measure surface similarity rather than semantic correctness, they cannot fully represent qualitative response quality.

Second, we applied a GPT-4o-based judger that classified each response as Totally Correct (TC), Partially Correct (PC), Incorrect (IC), or Indeterminate (ID) based on its relevance to the question, its semantic alignment with the ground-truth answer, and whether the question was answerable. This provided a meaning-level evaluation beyond lexical overlap. As shown in Fig. 3b, ALPaCA-GMM and ALPaCA-LongFormer achieved similar overall correct rates (the sum of TC and PC rates, 66–67%), with GMM exhibiting a slightly lower IC rate and LongFormer a higher TC rate. ALPaCA-Hybrid outperformed all variants, reaching a 70.67% overall correct rate, the highest TC rate (46.28%), and the lowest IC rate (19.31%).

We next examined how different ALPaCA variants performed across tumour (TCGA) and non-tumour (GTEx) cases and across question categories (Fig. 3c and Supplementary Fig. 3a, b). ALPaCA-GMM and ALPaCA-LongFormer achieved comparable performance on tumour-related questions, whereas GMM was weaker on GTEx questions, which often require recognising fine-grained histological features described in slide-specific notes. LongFormer performed best on tasks requiring regional or multi-region reasoning, such as grading, invasion, and cell-level morphology, while GMM excelled in global tasks, including tumour origin, diagnosis, and organ identification. By combining these complementary strengths, ALPaCA-Hybrid delivered the most consistent performance across all categories, showing clear advantages in both tumour micro-environment and tissue-structure-related tasks.

Lastly, to further assess clinical reliability, two consultant pathologists independently evaluated responses generated by ALPaCA-Hybrid. They followed the same evaluation criteria but, unlike the automated judger, also had access to the original slides for direct review. To quantify differences between expert interpretation and the GPT-4o judger, we report results across the full set of 1641 questions (Fig. 3d–f). Overall performance remained stable relative to the automated judger, with only a modest reduction in overall correctness and a decrease in incorrect classifications. Most discrepancies arose from tumour-related questions that experts judged unanswerable from a single slide (e.g., staging, margin status), which GPT-4o tended to label as IC rather than ID. Agreement between experts and GPT-4o was higher for GTEx than for TCGA cases, reflecting the greater noise in TCGA reports. Notably, many responses initially labelled as incorrect in diagnosis-related questions were upgraded by experts to TC or PC. These revisions arose for several reasons: in some cases, ALPaCA-Hybrid provided diagnostically correct but less granular subtypes (e.g., identifying lung adenocarcinoma instead of the more specific micropapillary variant); in others, responses aligned with updated diagnostic guidelines that merge rare subtypes into broader categories (e.g., breast medullary carcinoma now classified within infiltrating duct carcinoma); and in a subset of cases, discrepancies stemmed from ground-truth labels derived from ICD-O coding that did not fully reflect the actual clinical diagnosis on the slide. In all such instances, experts judged the model’s responses to be clinically appropriate.

More detailed analyses on open-ended performance across different cancer projects and tissue sites, as well as assessments by joint or individual expert pathologists, are presented in Supplementary Figs. 3c, d, 4.

### Evaluation against baseline models and independent clinical cohorts

**Fig. 4:**
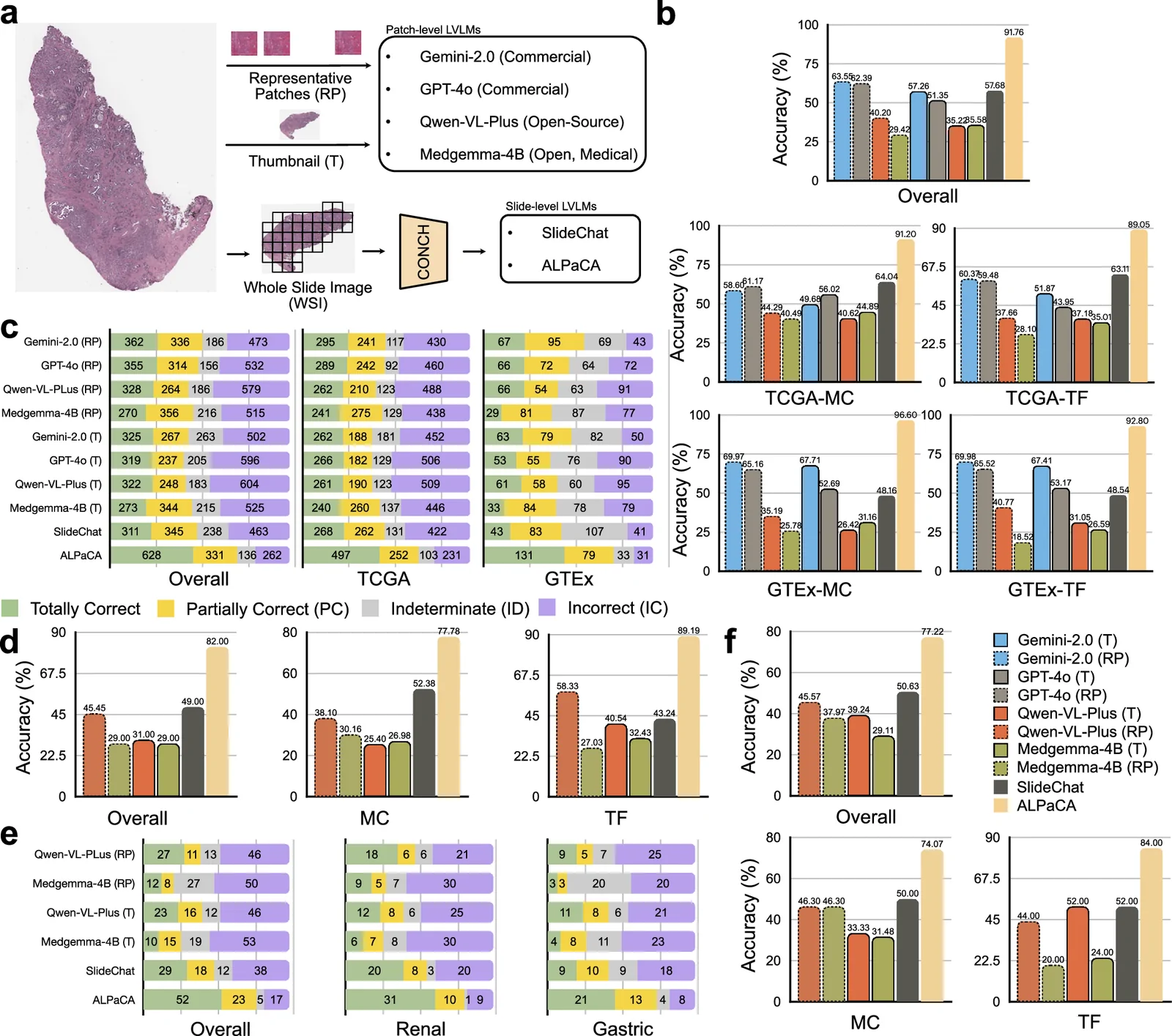
Performance comparison between ALPaCA and LVLM baselines. **a** Overview of the benchmarking setup. **b** Close-ended QA performance on the hold-out General Slide-QA test set, showing overall accuracy (*n *= 2403 questions) and separate results for multiple-choice (MC) and True/False (TF) questions across TCGA (*n *= 1467 questions) and GTEx (*n *= 936 questions). **c** Open-ended QA performance on the hold-out General Slide-QA test set, evaluated by the GPT-4o-based judger for answerable questions, reported overall (*n *= 1357 questions) and separately for TCGA (*n *= 1083 questions) and GTEx (*n *= 274 questions). **d** Close-ended QA performance of non-commercial baseline LVLMs and ALPaCA on the external XJTU-RG cohort (*n *= 100 questions; MC, *n *= 63; TF, *n *= 37). **e** Open-ended QA performance on the XJTU-RG cohort evaluated by the GPT-4o-based judger, reported overall (*n *= 97 questions) and stratified by cancer type, including renal cell carcinoma (*n *= 51 questions) and gastric cancer (*n *= 46 questions). **f** Close-ended QA performance on the external CUH-OV cohort (*n *= 79 questions; MC, *n *= 54; TF, *n *= 25). Source data are provided as a Source Data file.

To assess whether slide-level training and WSI-specific input handling provide advantages over existing LVLMs, we benchmarked ALPaCA-Hybrid against general-purpose LVLMs and SlideChat on both close-ended and open-ended QA tasks using the hold-out General Slide-QA test set. Since general-purpose LVLMs cannot directly process gigapixel WSIs, we evaluated them using two practical input strategies: WSI thumbnails and representative patches from each slide (Fig. 4a).

In close-ended evaluation, Gemini-2.0 achieved the highest accuracy among general-purpose LVLMs, followed by GPT-4o, whereas open-source models (QwenVL-Plus and MedGemma-4B) performed substantially worse (Fig. 4b). For the stronger general-purpose LVLMs, representative patches outperformed thumbnails, suggesting that low-resolution slide views are insufficient for reliable pathology reasoning. Despite Gemini-2.0 being the strongest general-purpose baseline, ALPaCA-Hybrid retained a substantial accuracy advantage of 28.21%. SlideChat performed slightly better than commercial LVLMs on TCGA tumour cases but dropped sharply on GTEx, consistent with its tumour-dominant training distribution. In contrast, ALPaCA-Hybrid maintained high accuracy across both tumour and non-tumour tasks, indicating broader domain coverage and stronger robustness. Beyond overall accuracy, we further evaluated clinically meaningful performance in two question categories, i.e., tumour grading and staging, and tumour detection, using confusion matrices, Cohen’s kappa, sensitivity, and specificity (Supplementary Fig. 5), where ALPaCA-Hybrid consistently outperformed competing LVLM baselines.

Open-ended evaluation using the GPT-4o-based judger showed consistent trends (Fig. 4c). SlideChat again performed best among baselines on TCGA tumour cases but poorly on GTEx, whereas Gemini-2.0 with representative patches was the strongest general-purpose LVLM, followed by GPT-4o. Even so, the strongest baseline lagged behind ALPaCA-Hybrid by 15% in overall correctness (TC + PC) and by approximately 10% in incorrect-response reduction. These results indicate that robust slide-level QA requires task-specific training and architectures that explicitly account for WSI-scale analysis.

We then assessed the real-world generalisability of ALPaCA-Hybrid using two independent clinical cohorts, evaluating its performance on external patient data and comparing it with the non-commercial LVLM baselines permitted under the corresponding data-use constraints. The two cohorts, i.e., XJTU-RG (20 patients) and CUH-OV (10 patients), comprising a total of 42 diagnostic slides, were sourced from different medical centres and, despite their modest sizes, encompassed diverse diagnostic subtypes within renal, gastric, and ovarian cancers. During evaluation, no background clinical information (e.g., cancer type) was provided to any model, and questions followed formats analogous to those in the General Slide-QA dataset. These settings enabled a direct assessment of each model’s capacity for general slide-level question answering under realistic clinical conditions.

For the XJTU-RG cohort, ALPaCA-Hybrid showed strong generalisability. In the close-ended evaluation (Fig. 4d), SlideChat achieved the highest accuracy among baseline LVLMs (49%), followed by Qwen-VL-Plus with representative patches (45.45%), while MedGemma-4B performed the worst, especially on True/False questions, where it frequently defaulted to the negative response No, resulting in accuracy below random guessing. ALPaCA-Hybrid maintained substantially higher performance, reaching 82% accuracy, even though performance moderately decreased under external-cohort conditions. In the open-ended evaluation (Fig. 4e), trends were consistent with the close-ended setting. ALPaCA-Hybrid achieved 75 out of 97 overall correctness (TC + PC), whereas the best baseline LVLM reached only 47 out of 97. Performance also varied across cancer types: results were higher for renal cancer cases than for gastric cancer cases, mirroring the relative difficulty differences observed in general pathology diagnosis.

For the CUH-OV cohort, ALPaCA-Hybrid exhibited a more pronounced performance drop, achieving 77.22% overall accuracy (Fig. 4f). True/False and multiple-choice QA accuracies decreased to 84% and 74.07%, respectively. Even with this decrease, ALPaCA-Hybrid still outperformed all non-commercial LVLM baselines by at least 27%, demonstrating clear advantages over existing LVLMs. This reduction is largely attributable to several dataset-level mismatches between CUH-OV and the public training sources (TCGA and GTEx). CUH-OV encompasses a wide spectrum of ovarian cancer subtypes, including high-grade serous, low-grade serous, clear cell, endometrioid, and serous borderline tumours; it also includes slides from multiple clinically relevant tissue sites such as fallopian tube, ovary, omentum, and pelvic. By contrast, TCGA-OV consists of only 107 cases, almost all annotated with coarse diagnostic labels (e.g., serous cystadenocarcinoma, high-grade serous carcinoma) and includes only primary-tumour sections without metastatic or multi-site sampling. Although GTEx contains small numbers of related normal tissues (e.g., fallopian tube or omentum under the Adipose-Visceral tissue category), these samples are sparse and lack disease-specific context around ovarian cancer. Consequently, ALPaCA has limited exposure to fine-grained subtype diversity and multi-site morphological variation in ovarian cancer, which contributes to the reduced performance on CUH-OV.

This observed degradation also highlights clear avenues for strengthening the slide-level LVLM, including expanding subtype coverage within each cancer, incorporating tumour samples from multiple anatomical sites beyond primary sections, and utilising more detailed, up-to-date pathology reports aligned with current diagnostic guidelines.

### Accommodating in-depth disease-specific question answering

**Fig. 5:**
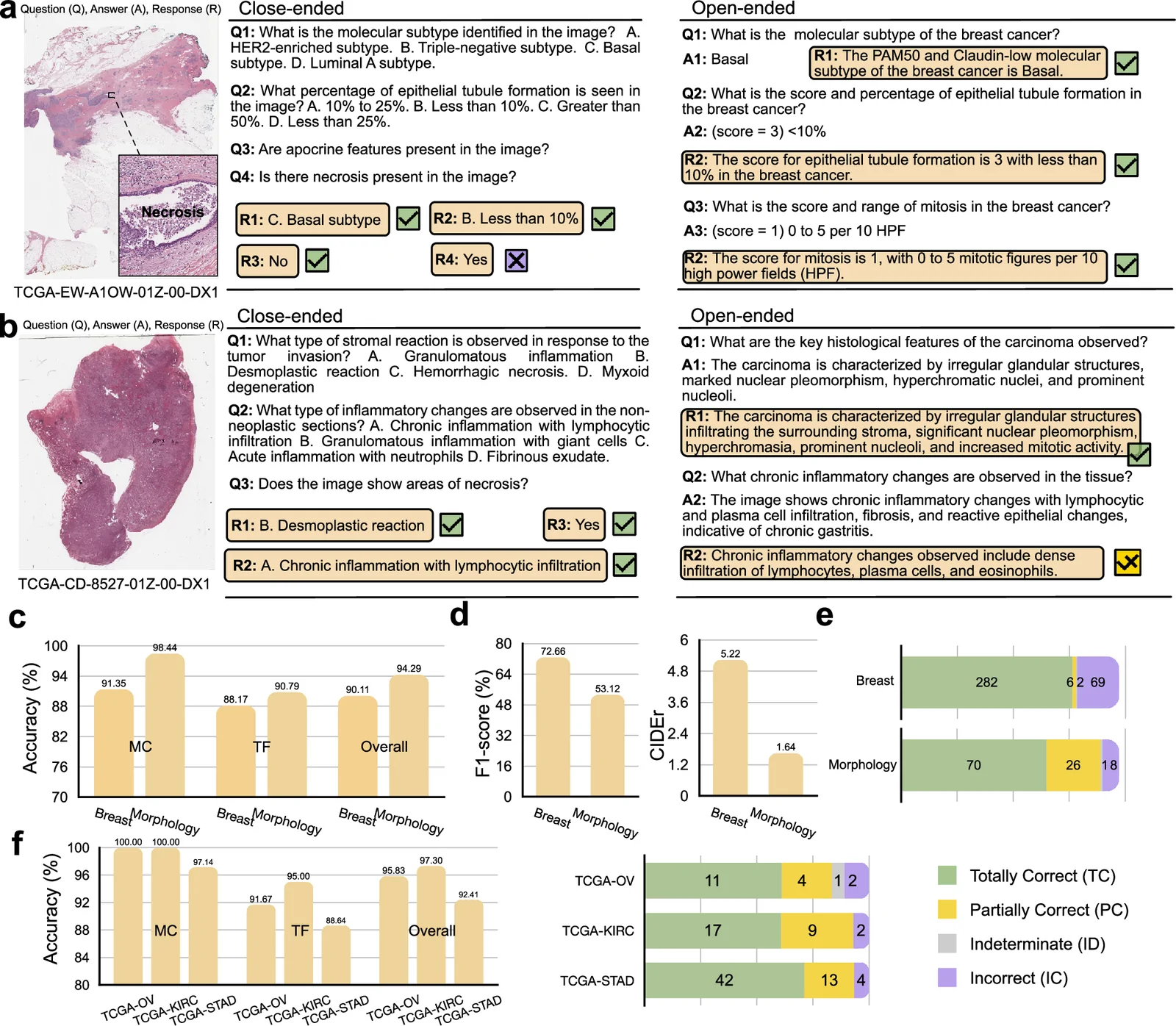
ALPaCA performance on specific QA datasets. **a**, **b** Example cases of a breast tumour slide from the Specific Slide-QA (Breast) dataset and a gastric tumour slide from the Specific Slide-QA (Morphology) dataset, with corresponding close- and open-ended questions, ground-truth answers, ALPaCA-Hybrid responses and GPT-4o-based judger assessments.** c** Close-ended performance of ALPaCA-Hybrid on the two specific datasets, including overall accuracy and separate accuracies for multiple-choice (MC) and True/False (TF) questions in Specific Slide-QA (Breast; *n *= 475 questions; MC, *n *= 289; TF, *n *= 186) and Specific Slide-QA (Morphology; *n *= 140 questions; MC, *n *= 64; TF, *n *= 76). **d**, **e** Open-ended performance of ALPaCA-Hybrid on the two specific datasets, including captioning metrics and detailed quantitative assessment results. Responses were evaluated by the GPT-4o-based judger for Specific Slide-QA (Breast; *n *= 359 questions) and by expert pathologists for Specific Slide-QA (Morphology; *n *= 105 questions). **f** Performance of ALPaCA-Hybrid across three cancer projects in the Specific Slide-QA (Morphology) dataset, including close-ended questions from TCGA-OV (*n *= 24), TCGA-KIRC (*n *= 37) and TCGA-STAD (*n *= 79), and open-ended questions from TCGA-OV (*n *= 18), TCGA-KIRC (*n *= 28) and TCGA-STAD (*n *= 59). Source data are provided as a Source Data file.

Finally, we evaluated the performance of ALPaCA-Hybrid after fine-tuning on two disease-specific QA datasets: Specific Slide-QA (Breast) and Specific Slide-QA (Morphology). The former focuses on detailed molecular and morphological aspects of breast cancer, including molecular subtypes, lymphovascular invasion, necrosis, nuclear pleomorphism, fibrous focus, mitosis, and other key pathological features. (Fig. 5a) The latter encompasses fine-grained histological morphology QA for gastric cancer, clear cell renal carcinoma, and ovarian cancer. (Fig. 5b)

Overall, ALPaCA-Hybrid achieved performances on the two disease-specific QA datasets that were comparable to or even exceeded its results on the general QA dataset. Specifically, in the Specific Slide-QA (Breast) test set, the close-ended accuracy was 90.11%, slightly lower than the 96.41% achieved on Specific Slide-QA (Morphology) (Fig. 5c). This discrepancy is attributed to the greater difficulty of questions in Specific Slide-QA (Breast), which includes not only molecular subtypes but also quantitative morphological questions. In contrast, while Specific Slide-QA (Morphology) focuses on more fine-grained morphological details, most questions involve identifying whether a particular morphological feature is present or determining its classification, making the close-ended task relatively easier.

For open-ended performance, ALPaCA-Hybrid notably outperformed in Specific Slide-QA (Breast), achieving a word-level F1-score of 72.66% vs. 53.12% and a CIDEr score of 5.22 vs. 1.64 compared to Specific Slide-QA (Morphology) (Fig. 5d). This is because Specific Slide-QA (Breast) open-ended questions were generated from structured tabular data, resulting in simpler, more standardised responses that the model could fully reproduce. In contrast, the Specific Slide-QA (Morphology) dataset contains free-text descriptions with higher variability, making it more challenging for the model to generate responses that exactly match the ground truth.

For Specific Slide-QA (Breast), open-ended responses were further assessed using the GPT-4o-based judger (Fig. 5e). In this dataset, ALPaCA-Hybrid correctly answered 288 out of 359 open-ended questions, including 282 TC responses, achieving an accuracy of over 80%. In contrast, for the Specific Slide-QA (Morphology) dataset, open-ended responses were evaluated by pathologists rather than the GPT-4o-based judger to ensure that this LLM-assisted benchmark reflects independent, pathologist-verified judgement rather than consistency with LLM-generated priors. Among the 105 open-ended questions, ALPaCA-Hybrid was rated as TC (70) or PC (26) on 91.43% of questions, with only 8 IC responses. This expert assessment suggests substantially stronger practical performance than implied by the lexical metrics such as CIDEr, indicating that when ALPaCA-Hybrid’s responses did not precisely match the reference answers, they often still conveyed similar meanings or contained the correct information. A breakdown of performance across the three cancer projects in this dataset is shown in Fig. 5f.

In addition, as shown in Fig. 5a, annotation errors exist in certain breast cancer cases. The slide was initially labelled as necrosis absent, whereas ALPaCA-Hybrid detected its presence. Upon further review, pathologists confirmed small necrosis areas in the central region of the slide, underscoring ALPaCA’s capability to identify overlooked pathological details.

## Discussion

In this study, we have introduced ALPaCA, a slide-level LVLM designed for WSI question answering. We demonstrated that connecting a foundation pathology vision encoder with an LLM via specialised adaptors enables effective slide-level QA. We also curated a large-scale WSI-level QA dataset spanning multiple cancer types and tissue sites, providing a foundation for developing LVLMs for WSI analysis. This demonstrated the feasibility of automatically constructing extensive WSI-level QA datasets. In addition, we designed a comprehensive evaluation framework for slide-level LVLMs, ranging from simple close-ended question accuracy to automated scoring of open-ended responses and expert pathologist assessments. After a detailed comparison of six candidate adaptors, we proposed the hybrid adaptor (ALPaCA-Hybrid), which achieved the best performance across multiple evaluation scenarios. Furthermore, we showed that fine-tuning ALPaCA on specific datasets enables in-depth, disease-specific interactive QA, supporting more complex and specialised pathology diagnostic workflows.

Despite these advancements, this work has certain limitations. First, although we curated one of the largest publicly available datasets for this task, the overall data volume remains relatively limited, particularly for rare tumours and real-world non-tumour cases. The training data are primarily derived from TCGA and GTEx cohorts, which cover a broad range of cancer types and tissue sites but may not fully reflect the diversity and heterogeneity encountered in real-world clinical settings. In addition, the GTEx database, which is largely composed of postmortem donor tissues, differs from the diagnostic slides and adjacent normal tissue sections typically encountered in routine clinical practice. Second, deriving slide-level supervision from patient-level pathology reports introduces an inherent mismatch: individual reports often summarise findings across multiple slides, anatomical sites, or sampling depths. Consequently, some generated questions cannot be reliably answered using a single WSI, as observed primarily in the TCGA subset. In contrast, the GTEx subset demonstrates that when textual descriptions are curated explicitly at the slide level, high-quality and less ambiguous QA pairs can be generated. This highlights the importance of either fine-grained per-slide annotation or multi-slide, patient-level reasoning frameworks for future pathology QA systems. Finally, ALPaCA does not incorporate explicit spatial grounding or region-level supervision, which limits clinical traceability for tasks requiring precise localisation or quantitative tissue assessment. While the model learns implicit region-answer associations via patch aggregation and attention, integrating explicit detection, segmentation, or region-aware supervision remains an important direction for future work. Moreover, ALPaCA is trained via instruction tuning without reinforcement learning-based optimisation; incorporating preference-based or reinforcement learning strategies may further improve decision consistency and uncertainty handling in clinical settings.

In conclusion, ALPaCA provides a slide-level LVLM framework for both research and clinical applications. With further refinement and validation on real-world datasets, ALPaCA can evolve into a more robust and clinically applicable slide-level LVLM. It has the potential to drive large-scale pathological data mining in cancer research while enhancing automated pathology reporting, diagnostic decision-making, and second-opinion validation in clinical practice. Furthermore, ALPaCA’s modular framework ensures adaptability to future advancements. Both the vision encoder and LLM backbone can be updated with the latest models as research progresses, enhancing performance and generalisation. Beyond its direct applications, ALPaCA’s framework, training strategy, and evaluation process provide a structured foundation for future advancements in slide-level LVLMs, contributing to the development of more robust and clinically relevant AI-driven WSI analysis solutions.

## Methods

### Ethics statement

This research complied with all relevant ethical regulations. Publicly available de-identified TCGA and GTEx data were used in accordance with the terms and conditions of the respective data providers. The collection and use of data from the XJTU-RG cohort were approved by the Ethics Committee of the First Affiliated Hospital of Xi’an Jiaotong University (KYLLSL2021-420 and KYLLSL2022-333), which approved a waiver of informed consent because the study was retrospective and all patient data were fully anonymised before analysis. The study involved no participant contact or intervention and no access to personally identifiable information or protected health information. The CUH-OV cohort was derived from the prospective Cambridge Translational Cancer Research Ovarian Study 04 (CTCR-OV04) at Addenbrooke’s Hospital, Cambridge University Hospitals NHS Foundation Trust. The study was approved by the Cambridgeshire and Hertfordshire Research Ethics Committee (REC reference: 08/H0306/61), and all participants provided written informed consent for research use of their donated samples.

This study analysed de-identified WSIs and associated pathology reports or notes. Sex and gender were not variables of interest in the study design or analysis and were not used as model inputs, labels or covariates. Although sex metadata are available for some TCGA and GTEx cases, they were not analysed in the present study. No sex or gender data were collected for XJTU-RG, and the study team made no independent determination of sex or gender by self-report or assignment. The parent CTCR-OV04 study reported all CUH-OV participants as female, but did not report gender identity or the method of sex ascertainment. Accordingly, no sex- or gender-stratified analyses were performed because the study focused on developing and evaluating slide-level question answering from WSIs and associated pathology reports, rather than assessing participant-level sex- or gender-associated differences.

### Curation of datasets

#### Slide caption dataset

First, we curated a slide-level caption dataset, General Slide-Caption, comprising 35,913 WSIs with corresponding descriptions. The source data were derived from the two largest publicly available human tissue databases: TCGA, which includes 32 cancer studies and 134 diagnosis types, and GTEx, which spans 40 non-tumour tissue sites. For TCGA, each WSI (FFPE diagnostic slide stained with H&E at 40x or 20x magnification) is paired with a patient-level pathology report (in PDF format) and basic patient metadata. The Python package PyMuPDF converted PDF files into text files. For PDFs in image format, which could not be processed automatically, optical character recognition (OCR) techniques were applied. The raw pathology reports often included irrelevant sections, such as gross examination. We used the commercial solution ChatGPT (GPT-3.5 Turbo) to summarise each raw report into a structured text description. This process excluded any gross examination information while retaining all microscopic pathological findings. Next, we refined these descriptions using the general-purpose version of ChatGPT (GPT-4o) with patient metadata and well-designed prompts. This step ensured that the descriptions were accurate and tailored to specific patient contexts, focusing exclusively on the content of primary tumour slides, the main type of TCGA diagnostic slides. For GTEx, each WSI (FFPE diagnostic slide stained with H&E at 20x magnification) is accompanied by a pathology note and basic tissue metadata. Since the pathology notes are well-structured and specific to the corresponding WSI, we only rephrased them using GPT-4o with the aid of tissue metadata and carefully designed prompts. This process excluded any quantitative tissue information (e.g., length, thickness) that is hard to infer from WSIs.

#### General slide question-answering dataset

Second, we curated a slide-level QA dataset, General Slide-QA, comprising 341,051 instructions. This dataset includes True or False and Multiple-Choice close-ended Q&A pairs, along with open-ended pairs requiring free-text responses. Based on the General Slide-Caption dataset, slide-level Q&A pairs were generated by GPT-4o using prompts with clear guidelines, designed by experienced pathologists, tailored for close-ended and open-ended scenarios of tumour and non-tumour cases, respectively.

#### Specific slide question-answering datasets

Lastly, two specific and in-depth slide-level QA datasets were curated: Specific Slide-QA (Breast), focusing on TCGA-BRCA cases, and Specific Slide-QA (Morphology), covering TCGA-STAD, TCGA-KIRC, and TCGA-OV cases. Instead of utilising the general descriptions generated for the General Slide-Caption dataset, we incorporated additional molecular annotations of TCGA-BRCA cases, as provided in ref. ^45^, to create an in-depth molecular QA dataset. Furthermore, we leveraged the detailed morphological descriptions generated by PathChat, the state-of-the-art pathology AI copilot^36^, to construct the in-depth morphology QA dataset. Specifically, for each WSI, we extracted 1024 × 1024 patches at 40 × , 20 × , and 10 × magnifications and applied KMeans clustering to obtain 32, 16, and 8 representative clusters, respectively. PathChat was then used to generate morphological descriptions for the patches closest to each cluster centre, and these descriptions were subsequently summarised into comprehensive slide-level reports by GPT-4o. Using these enriched annotations and descriptions, we curated specific, detailed, close-ended, and open-ended Q&A pairs tailored to breast, gastric, renal, and ovarian cancers.

#### Evaluation datasets

For testing and evaluation, we randomly selected 3 cases per non-tumour tissue site from the GTEx database and 1 or 2 cases per diagnosis type from the TCGA database as internal hold-out test cases, totalling 337 cases, with the remaining cases used for training. Notably, these hold-out cases were excluded from all stages of training to prevent data leakage, ensuring an unbiased evaluation. This split was applied consistently across the three curated datasets: General Slide-QA, Specific Slide-QA (Breast), and Specific Slide-QA (Morphology).

In addition, to ensure the reliability of the evaluation benchmark and mitigate potential inconsistencies introduced during the PathChat-assisted description generation process, we further reviewed the Specific Slide-QA (Morphology) test set with expert pathologists. For the close-ended Q&A pairs, pathologists reviewed all items against the corresponding whole-slide images. Of the 167 close-ended Q&A pairs, 140 were retained, including 5 with corrected answers, while 27 were removed because the questions were incorrect or could not be reliably answered from the current slide. For the open-ended Q&A pairs, 111 questions were reviewed, and 6 incorrect questions were removed, leaving 105 valid items for expert scoring.

To further assess the model’s generalisation ability in real-world clinical settings, we incorporated two external validation cohorts. (1) XJTU-RG: This cohort comprised 20 patients (10 renal cell carcinoma and 10 gastric cancer), with one or two diagnostic slides per patient (21 slides in total). A total of 197 question-answer pairs were constructed, including 97 open-ended and 100 close-ended Q&A pairs. (2) CUH-OV: This cohort included 10 ovarian cancer cases, each with 1-3 slides, resulting in 21 slides and 79 close-ended Q&A pairs. Open-ended evaluation was not conducted for this cohort due to data governance restrictions. For both external cohorts, close-ended questions were directly authored by pathologists based on slide-level assessment. Open-ended questions, where permitted by data governance, were derived from original pathology reports through an LLM-assisted summarisation and question-generation process to ensure consistency with the General Slide-QA schema, followed by manual review and refinement by pathologists. All questions and answers were created and validated directly on the corresponding diagnostic slides to ensure accuracy and clinical validity.

Details of the source data are presented in Supplementary Table 1, while information on the curated datasets and data splits is provided in Supplementary Table 2. Additional statistical insights and example questions of the General Slide-QA dataset are illustrated in Supplementary Fig. 1a–f and Supplementary Tables 3–6. All prompts used for data curation are detailed in Supplementary Note 5: Prompts.

#### Quality control

Given that the General Slide-Caption and General Slide-QA datasets form the foundation of model training and rely on LLM-generated text, we conducted a multi-stage quality assessment to evaluate their reliability and quantify potential noise introduced by automated summarisation and Q&A generation. First, we performed a targeted assessment of 40 randomly sampled cases (20 TCGA from different cancer projects and 20 GTEx from distinct tissue sites). For each case, pathologists reviewed the original PDF pathology reports (TCGA) or text notes (GTEx) and compared them against the LLM-generated summaries, with completeness and hallucination assessed independently. Minor omissions were observed in 6 TCGA cases (4 missing stage information and 2 missing venous invasion status), all attributable to OCR misalignment in table-formatted reports; GTEx showed one omission related to aliquot thickness, a detail not relevant to slide interpretation. These missing elements have minimal impact on downstream QA generation, as such attributes are never used as question targets in the QA generation schema. Hallucinations were rare, one TCGA case (reactive stromal cells) and one GTEx case (clean specimen). Both arose from reasonable but report-unsupported inferences, and their overall low frequency reflects the effectiveness of our prompts that restrict generation to report-verifiable content.

Then, we further evaluated the 430 QA pairs associated with the same 40 cases by inspecting the diagnostic slides, assessing each item for both answer correctness and answerability given the information visible on the slide. Among TCGA QAs (*n *= 271), 262 answers were correct, and 228 were answerable. Inaccuracies arose mainly from over-inference based on stage or tumour type (e.g., inferring patterns of tumour involvement, enumerating expected cell types, or generating unsupported N-stage assignments). Unanswerable items primarily required information not present on a single slide (e.g., margin status, lymph-node involvement, advanced staging). For GTEx QA (*n *= 159), 158 answers were correct, and 153 were answerable; the single incorrect answer concerned fibrosis not mentioned in the note but present on the slide, and the unanswerable questions involved fine-grained tissue provenance (e.g., sun-exposed vs non-sun-exposed skin).

Overall, the LLM-generated training data exhibited high fidelity (Supplementary Fig. 1g, h). For captions, 10–20% of cases contained minor omissions, but only involving small ancillary details, and hallucinations occurred in approximately 5% of cases, almost entirely consisting of plausible inferences. For QA pairs, about 98% of answers were correct, and 88% were answerable based on the information visible on the slide. These findings indicate that the curated data provide reliable supervision for model training with limited synthetic noise. The noise level was slightly higher in TCGA than in GTEx, largely due to the use of PDF-formatted patient reports in TCGA, in contrast to the more structured text-based slide notes available in GTEx.

### Pre-processing of WSIs

Based on the general pre-processing pipeline for WSI analysis, we began by detecting tissue regions in each WSI and dividing them into small patches with a 50% overlap. Since different questions for the same case or the same question across different cases may require information from varying levels of the WSI or their combination, WSI tessellation was performed hierarchically. Patch sizes were set to 1024, 2048, and 4096 at 40x magnification. If the maximum magnification of the original slide was 20x, the patch sizes were adjusted to 512, 1024, and 2048, respectively, to maintain consistency. These patches were then resized to a uniform size (224 × 224), mimicking tiles at 40x, 20x, and 10x magnifications to capture information across multiple levels of each WSI. Then, we used the frozen CONCH^42^ encoder to extract features from patches at different magnifications, forming hierarchical patch embeddings. CONCH is a state-of-the-art pathology image encoder aligned with text using 1.17 million image-caption pairs, making it well-suited for multi-magnification feature extraction.

### ALPaCA architecture and various adaptors

Built upon the hierarchical patch embeddings of each WSI, it is necessary to compress and aggregate the patch embeddings into corresponding WSI tokens for two primary reasons: (1) the raw patch embeddings are too sparse, containing redundant information, and (2) the sequence length of uncompressed patch embeddings would be excessively long, making it impractical for LLM training. To achieve this, six distinct types of adaptors were proposed and implemented, designed to aggregate patch embeddings at different magnifications into WSI tokens. These tokens were then concatenated with text tokens in a specific structure for LLM training. Specifically, we adopted the 8-billion-parameter version of Llama3.1, a decoder-only transformer-based autoregressive language model, as the LLM for ALPaCA because it provides strong language-modelling capability, public accessibility, and manageable training cost for academic research. We implemented four vision-only adaptors and two vision-text interactive adaptors. The key difference lies in whether the adaptors incorporate any text context, such as questions, during the compression and aggregation of WSI tokens. The detailed descriptions of each adaptor are as follows:

- Sampling: The most straightforward approach involves sampling a subset of patch embeddings to represent the WSI. To ensure the representativeness of the sampling, patch embeddings are grouped into clusters using the KMeans algorithm, and one patch embedding is randomly sampled from each cluster to serve as a token in the final WSI representation.
- KMeans: Another straightforward method involves clustering the patch embeddings and using the centroid of each cluster as a token in the WSI representation. This approach ensures that each token represents the central tendency of the corresponding cluster, providing a compact and representative summary of the WSI.
- GMM^46^: A prototype-based approach rooted in the Gaussian Mixture Model (GMM) is used to summarise patch embeddings into a set of morphological prototypes. Specifically, each patch embedding is assumed to be generated from a mixture distribution, where each mixture component represents a morphological exemplar. The resulting prototypes provide a compact representation of the WSI by capturing the dominant patterns present in patches.
- ABMIL^47^: A classic aggregation method in WSI analysis, Attention-Based Multi-Instance Learning (ABMIL) uses a multi-head gated attention network to aggregate patch embeddings into diverse WSI tokens. This method dynamically assigns attention weights to patches, ensuring that the features most relevant to correct answers contribute most to the final WSI representation.
- Qformer^27^: A vision-text interaction adaptor that incorporates both self-attention and cross-attention layers. It begins by initialising multiple learnable query tokens, which are first refined through a self-attention mechanism applied to the query tokens and question embeddings (from the LLM tokeniser). The optimised query tokens are then used in the cross-attention layers to query the patch embeddings, enabling question-specific refinement of the query tokens for the downstream QA task. To simplify the adaptor, we utilised a lightweight design for each magnification level. Specifically, a BERT encoder with a single hidden layer and a single cross-attention layer was employed.
- LongFormer: A custom-designed vision-text interaction adaptor, LongFormer differs from QFormer by not merely querying fixed patch embeddings for task-specific WSI representations but also enabling the optimisation of patch embeddings. By utilising a LongNet structure^48^, it efficiently handles ultra-long sequence feature optimisation, allowing for better integration and refinement of information across the entire WSI. This design ensures that patch embeddings dynamically adapt to the specific task requirements, enabling the generation of more precise and context-aware WSI tokens.

All adaptors produced 32, 16, and 8 WSI tokens for 40x, 20x, and 10x magnifications, respectively. A resampler (composed of one hidden layer, ReLU activation, and a dropout rate of 0.25) then maps these WSI tokens to the required 4096 dimensions for Llama3.1.

Except for the GMM adaptor, which produces 1025-dimensional tokens, all other adaptors generate 512-dimensional tokens, consistent with the original patch embedding dimensions. Note that Sampling, KMeans, and GMM adaptors do not include trainable parameters aside from the resampler. ABMIL uses a trainable gated attention network with three linear layers and 256 hidden units. QFormer and LongFormer have a reducer with one hidden layer that maps question tokens from 4096 to 512 dimensions, followed by a layer normalisation. The cross-attention part for both of them is implemented using a single MultiheadAttention layer with 512 hidden states and 8 heads. For the self-attention part, QFormer employs a BertModel with one hidden layer, 512 hidden states, and 8 heads. LongFormer uses a regular transformer block with one self-attention layer (512 hidden states, 8 heads) and one feed-forward layer (2048 hidden states). In addition, LongFormer integrates a two-layer LongNet module to efficiently model long-range dependencies among patch tokens. Each LongNet layer operates with 512 hidden states and 8 attention heads, and adopts a dilated segment-based sparse attention scheme rather than sliding-window attention. Specifically, multiple dilated segments are used with dilation ratios {1, 2, 4, 8, 16} and corresponding segment lengths {1024, 1448, 2048, 2896, 4096}, enabling multi-scale contextual aggregation over gigapixel WSIs. The number of visual tokens is determined by the number of extracted patches per slide, while positional encodings support up to 10^6^ patch positions.

Moreover, due to the complementary performance of GMM and LongFormer adaptors in answering different types of questions, the best-performing ALPaCA variant (ALPaCA-Hybrid) is derived from their combination. Specifically, the WSI tokens extracted by both adaptors are combined additively after the resampler layer and then fed into Llama3.1.

### Three-stage training of ALPaCA

For training, we adopted a three-stage strategy, with the first two stages commonly used in LVLM training. The first stage, Comprehension, involves freezing the LLM weights while updating only the adaptors. This stage is supervised, with ALPaCA trained to predict the description corresponding to each WSI in the General Slide-Caption dataset. The adaptors learn to aggregate patch tokens into WSI tokens and project the image space into the shared embedding space of text tokens utilised by the LLM. The second stage, Instruction, involves training both the LLM and the adaptors end-to-end. In this stage, ALPaCA is trained to generate responses to diverse general instructions from the General Slide-QA dataset. The third stage, Specialisation, fine-tunes both the LLM and adaptors end-to-end on specific and in-depth datasets. In this stage, ALPaCA is fine-tuned to address domain-specific questions related to a particular tissue or cancer type. For this, we use two Specific Slide-QA datasets, one focusing on the molecular aspects of breast cancer and the other on the fine-grained morphological patterns of gastric, renal, and ovarian cancers.

Specifically, we use the next-word prediction loss across all three stages, treating ALPaCA as an autoregressive language model. The loss is calculated only for the answer text tokens, and the prompt is included solely for close-ended instructions. The structure of the LLM input list is as follows:

<∣Image∣> <∣H∣> 40x WSI tokens <∣M∣> 20x WSI tokens <∣L∣> 10x WSI tokens <∣Question∣> question text tokens <∣Prompt∣> prompt text tokens <∣Answer∣> answer text tokens <∣pad∣> <∣pad∣> <∣pad∣>

Note that the first stage also follows this input structure, transforming the captioning task into a QA task by setting several fixed questions to guide the caption generation. The learning rate was set to 2.0e-5, with a gradient accumulation step size of 8. The training batch size was 4, and the warmup phase included 1000 steps. The training process spanned 20 epochs for the first stage and 5 epochs for the second and third stages. To prevent out-of-memory failures caused by exceptionally large WSIs, we capped the maximum number of patch tokens per slide at 15,000. Under this constraint, training remained stable, with a peak memory usage of 68.29 GB per GPU during the second and third stages, where the LLM backbone was fully fine-tuned.

The total training time amounted to approximately 60 GPU hours for Stage 1, 634 GPU hours for Stage 2, and 50.8 and 28.5 GPU hours for Stage 3 on the Specific Slide-QA (Breast) and Specific Slide-QA (Morphology) datasets, respectively.

### Evaluation

We compared different versions of ALPaCA, equipped with various adaptors or trained using different strategies. For close-ended questions, all versions of ALPaCA generated predicted choices in a consistent format (e.g., ‘Yes’, ‘A. Grade III’), enabling direct calculation of evaluation metrics such as accuracy. For open-ended questions, we computed the word-level macro F1-score and the CIDEr score against the ground truth answers, with the CIDEr score being commonly used in captioning tasks. However, these automatic quantitative metrics often fail to reflect the quality of predictions accurately. To address this, we conducted further evaluations using a prompt-guided GPT-4o judger alongside manual assessments by professional pathologists.

All inference experiments were conducted in full FP32 precision using pre-extracted and cached WSI patch features. Under this setting, ALPaCA-Hybrid required approximately 37-38 GB of GPU memory across batch sizes ranging from 1 to 16 and achieved an average inference latency of 0.22 s per question. These measurements reflect only the multimodal reasoning stage and exclude offline WSI feature extraction, which is performed once per slide.

#### Evaluating with GPT-4o

We began by designing detailed assessment guidelines for GPT-4o, which are provided in Supplementary Note 5: Prompts. Since no commercial models currently support direct WSI input, we provided GPT-4o with the question, the corresponding ground truth answer, and ALPaCA’s response for evaluation. To minimise the randomness of GPT-4o evaluations, each response was assessed five times, and the final evaluation result was determined by taking the majority vote across the five evaluations. GPT-4o primarily assessed the degree of alignment between these elements and categorised each response into one of four options: totally correct, partially correct, indeterminate, or incorrect, considering both the consistency between the ground truth answer and ALPaCA’s response, as well as the content of the question itself. It is worth noting that even though the ground truth answers were derived from pathology reports or notes, these reports are typically written for a collection of slides from each patient and can sometimes be overly general. As a result, the ground truth may lack completeness or fail to align perfectly with the single WSI observed by ALPaCA. Moreover, for open-ended questions, multiple valid answers often exist that may be correct but do not overlap entirely. These factors highlight the limitations of evaluations solely based on the alignment between the ground truth and the model’s response without directly examining the corresponding WSIs.

#### Evaluating with expert pathologists

With access to the original WSIs, pathology reports/notes, and patient metadata, two consultant pathologists evaluated the best variant (Hybrid) of ALPaCA’s responses to open-ended questions. To ensure objectivity and consistency, the evaluation process was conducted as follows:

- Step 1: Joint evaluation of a subset of cases (one case per tissue or tumour type). This step aimed to establish a consistent evaluation standard and ensure alignment between the two pathologists.
- Step 2: Independent evaluation of the remaining cases by each pathologist. This increased the efficiency of the evaluation process.
- Step 3: Joint discussion for cases that were difficult to determine. This step resolved ambiguities and ensured consensus on challenging cases.

In alignment with the GPT-4o evaluation process, each response was categorised into one of four options: totally correct, partially correct, indeterminate, or incorrect. Since they had access to the original WSIs, their evaluations were based on both the questions and the exact slides analysed by the model rather than solely on answers extracted from reports or notes. This greatly enhanced the reliability of the assessments. In addition, the GPT-4o evaluation results were provided for their reference.

### Benchmarks

We compared ALPaCA’s performance with five representative LVLMs using the same automatic evaluation procedures described above.

- General-purpose LVLMs: We benchmarked four representative LVLMs widely used in multimodal reasoning tasks: Gemini-2.0^49^, GPT-4o^24^, QwenVL-Plus^50^, and MedGemma-4B^51^. Here, GPT-4o is evaluated purely as a multimodal LVLM baseline, distinct from the GPT-4o-based judger used in our automatic evaluation pipeline. Because these models cannot directly process gigapixel WSIs, we evaluated them using two input strategies: (1) full-slide thumbnails with the shorter side resized to 1024 pixels; and (2) 32 representative patches per slide, obtained by applying K-means clustering (*K* = 32) to CONCH feature embeddings and selecting the patch closest to each cluster centroid.
- Pathology-specific LVLM: We further included SlideChat^41^, a pathology-oriented slide-level LVLM equipped with domain-specific priors, whose training data are predominantly sourced from tumour slides.

### Hardware and software

Python (v3.10.4) was used for all experiments and analyses in this study. All three stages of ALPaCA were trained on two 80 GB NVIDIA A100 GPUs configured for distributed training, utilising the PyTorch (v2.2.1) deep learning framework with CUDA 11.8. Training acceleration was achieved through the integration of Accelerate (v0.31.0) with DeepSpeed (v0.14.0). For inference, all experiments were conducted on a single NVIDIA A100 (80 GB) GPU.

### Reporting summary

Further information on research design is available in the Nature Portfolio Reporting Summary linked to this article.

## Supplementary information

Supplementary Information

Reporting Summary

Transparent Peer Review file

## Source data

Source Data

## Data Availability

The TCGA WSIs, pathology reports, and associated clinical metadata are accessible via the NIH Genomic Data Commons (https://portal.gdc.cancer.gov). The GTEx WSIs, pathology notes, and associated clinical metadata are available from the Genotype-Tissue Expression project (https://www.gtexportal.org/). The curated datasets are publicly available on Hugging Face, including General Slide-Caption (CNX-PathLLM/TCGA-WSI-Description-4onew and CNX-PathLLM/GTEx-WSI-Description); General Slide-QA (CNX-PathLLM/TCGA-WSI-CloseQA-Balanced, CNX-PathLLM/GTEx-WSI-CloseQA-Balanced, CNX-PathLLM/TCGA-WSI-OpenQA and CNX-PathLLM/GTEx-WSI-OpenQA); Specific Slide-QA (Breast) (CNX-PathLLM/TCGA-BRCA-Details-CloseQA and CNX-PathLLM/TCGA-BRCA-Details-OpenQA); and Specific Slide-QA (Morphology) (CNX-PathLLM/PathChat-CloseQA-Balanced and CNX-PathLLM/PathChat-OpenQA). The datasets are available at https://huggingface.co/CNX-PathLLM/datasets. The two external validation cohorts (XJTU-RG and CUH-OV) contain patient-derived clinical pathology data and cannot be made publicly available because of patient privacy, ethical approval and data protection restrictions. Access requires approval from the corresponding institutional data controllers and ethics/governance committees and completion of an institutionally approved data-use agreement. XJTU-RG access requests should be directed to Chunbao Wang (bingliziliao2012@163.com) and reviewed by the First Affiliated Hospital of Xi’an Jiaotong University. CUH-OV access requests should be directed to Mireia Crispin-Ortuzar (mc973@cam.ac.uk) and reviewed by Cambridge University Hospitals NHS Foundation Trust and the CTCR-OV04 study governance body. Data can only be shared for non-commercial research purposes where permitted by the applicable approvals and data-transfer agreements. Source data are provided in this paper.

## Code availability

The code for training and evaluating ALPaCA is publicly available under the MIT License at https://github.com/ZeyuGaoAi/ALPaCA. Documentation of the model architecture, training procedure and evaluation protocols is provided in the repository. The repository also includes non-weight resources required for the released workflows. The three fine-tuned checkpoints are separately available at https://huggingface.co/CNX-PathLLM/Llama-slideQA. These checkpoints are derivative Llama 3.1 materials and are distributed under the Llama 3.1 Community License. Users must separately obtain access to the Llama-3.1-8B-Instruct base model under Meta’s applicable terms.
