# Supplementary material for: ALPaCA: Adapting Llama for Pathology Context Analysis to enable slide-level question answering
Source: Nat Commun. 2026 Aug 20;17:9943. doi: 10.1038/s41467-026-76372-z (PMC13586377; doi:10.1038/s41467-026-76372-z)
Supplement: Supplementary file 1 [file 41467_2026_76372_MOESM1_ESM.pdf]

Supplementary Information for:

ALPaCA: Adapting Llama for Pathology Context Analysis to enable slide-level question answering

Zeyu Gao[1,2], Kai He[3], Weiheng Su[4], Xiaobo Pang[4], Ines P. Machado[1,2], Mercedes Jimenez-Linan[2,5], Brian Rous[5], Chunbao Wang[4,6], Chengzu Li[7], William McGough[1,2], Shangqi Gao[1,2], Di Zhang[4], Tieliang Gong[4], Ming Y. Lu[8], Faisal Mahmood[8], Mengling Feng[3], Chen Li[4] and Mireia Crispin-Ortuzar[1,2]

[1]Department of Oncology, University of Cambridge, Cambridge, UK
[2]Cancer Research UK Cambridge Centre, University of Cambridge, Cambridge, UK
[3]Saw Swee Hock School of Public Health, National University of Singapore, Singapore
[4]School of Computer Science and Technology, Xi'an Jiaotong University, Xi'an, China
[5]Department of Pathology, Cambridge University Hospitals, Cambridge, UK
[6]Department of Pathology, First Affiliated Hospital of Xi'an Jiaotong University, Xi'an, China
[7]Language Technology Lab, University of Cambridge, Cambridge, UK
[8]Department of Pathology, Harvard Medical School, Boston, MA, USA

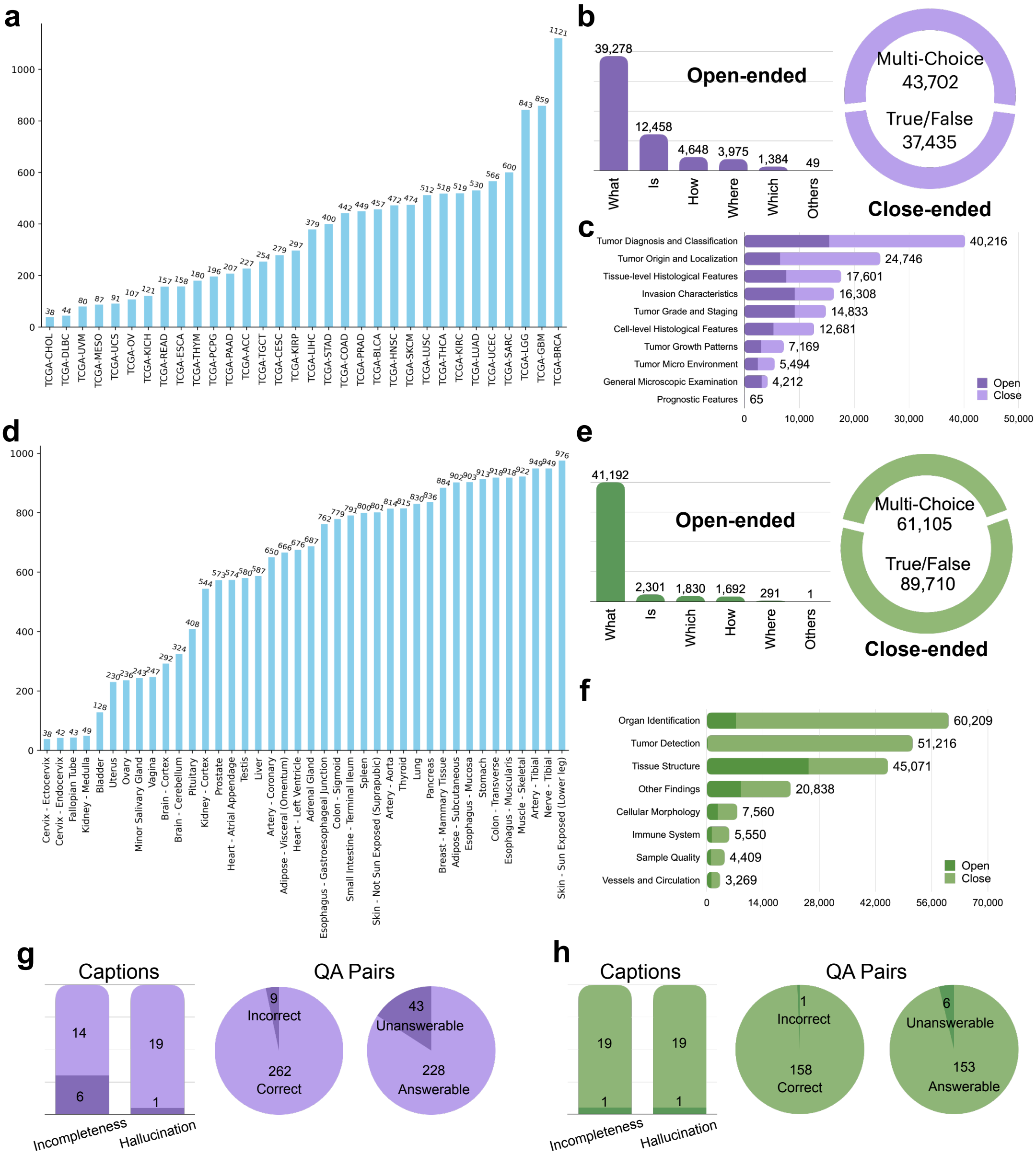

Supplementary Figure 1: Dataset Distribution. a, Case distribution across different cancer projects in TCGA. b, Distribution of question types based on their nature in TCGA cases from the General Slide-QA dataset. c, Distribution of question categories in TCGA cases from the General Slide-QA dataset. d, Case distribution across different tissue sites in GTEx. e, Distribution of question types based on their nature in GTEx cases from the General Slide-QA dataset. f, Distribution of question categories in GTEx cases from the General Slide-QA dataset. g, h, Quality assessment of captions and QA pairs in TCGA and GTEx.

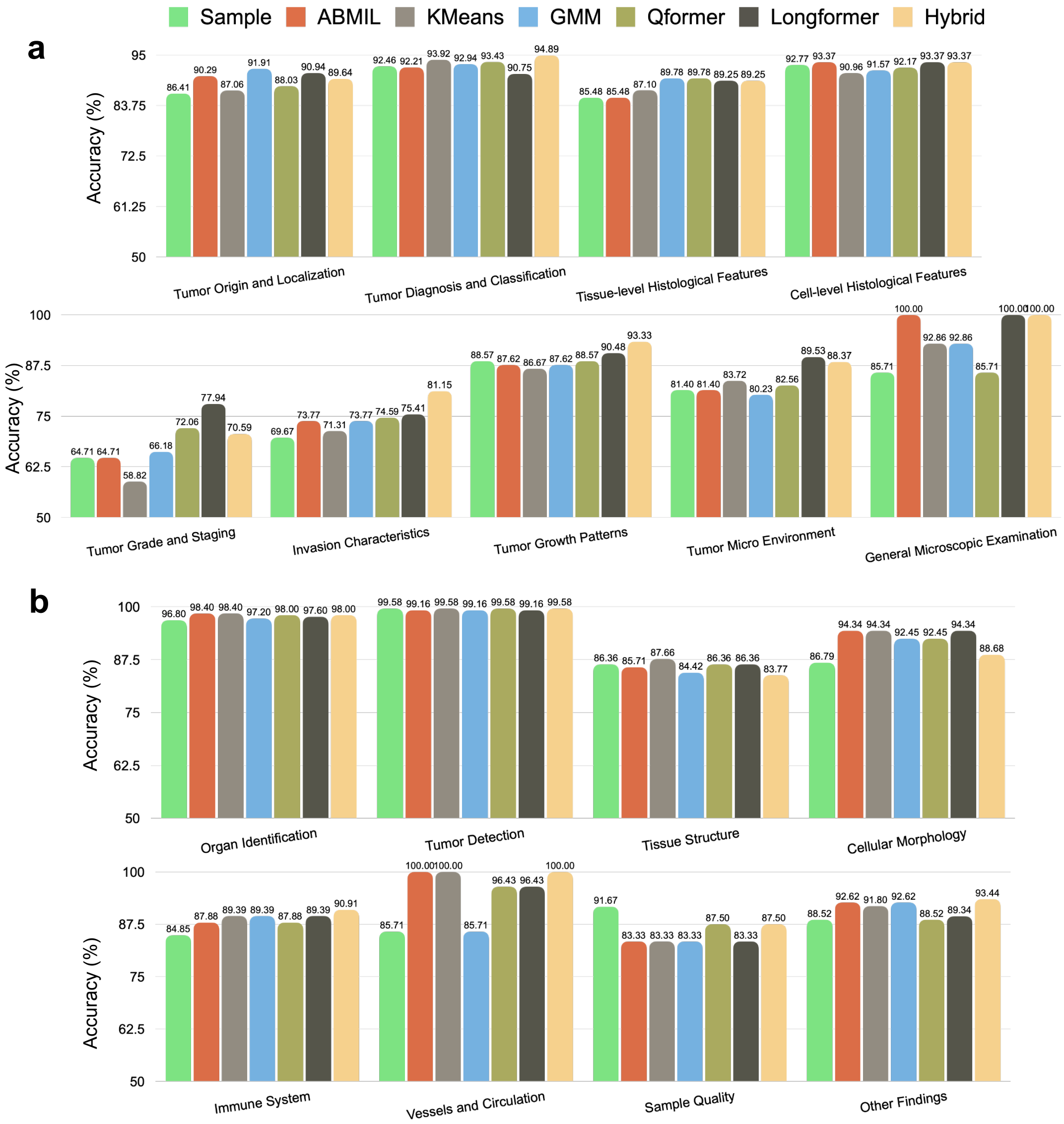

Supplementary Figure 2: Close-ended performance (accuracy) of ALPaCA variants on the test set of the General Slide-QA dataset. a, Performance across question categories in TCGA cases (n=1,467 questions). b, Performance across question categories in GTEx cases (n=936 questions).

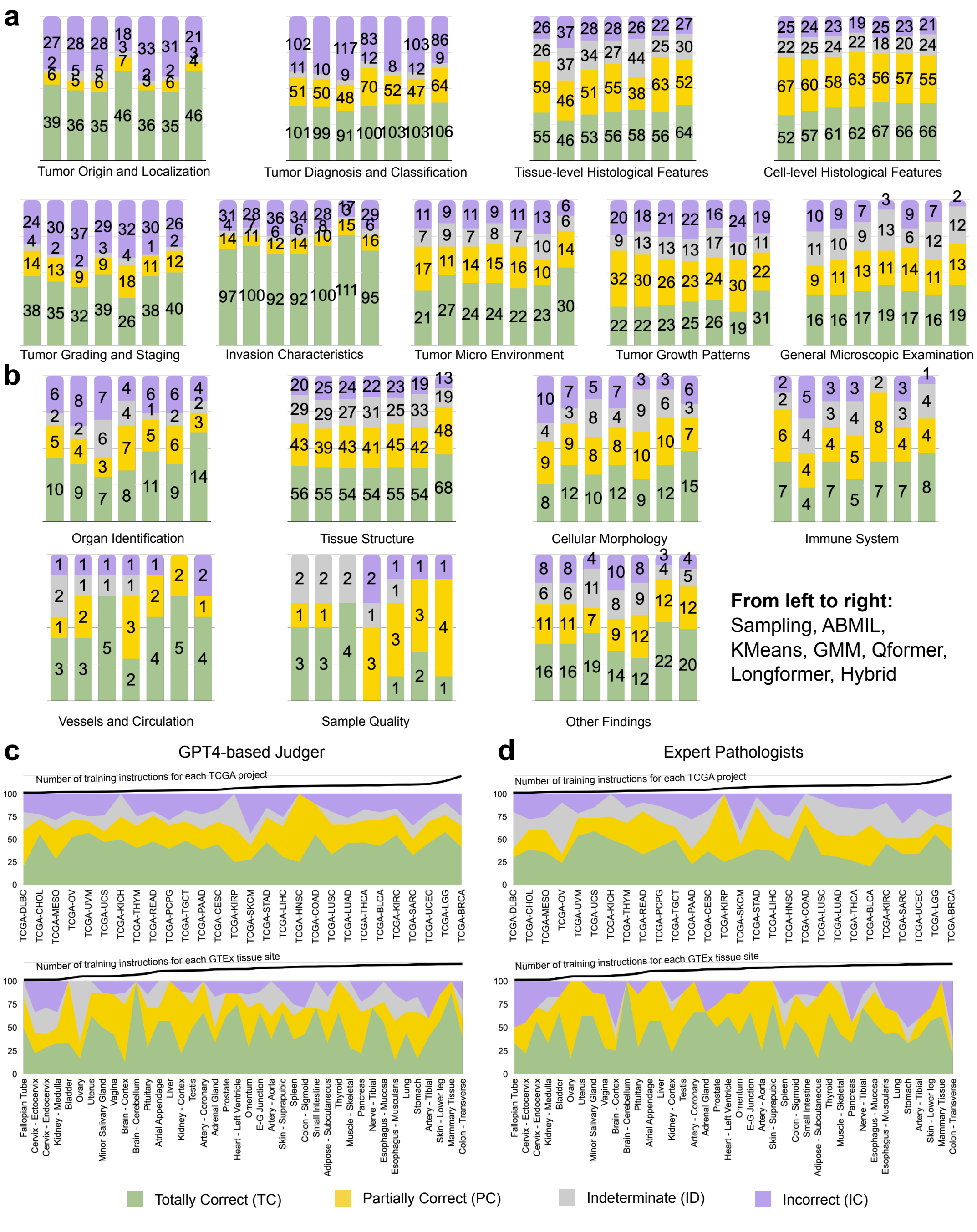

Supplementary Figure 3: Open-ended performance of ALPaCA variants on the test set of the General Slide-QA dataset. a, Performance across question categories in TCGA cases assessed by the GPT-4o-based judger on answerable questions (n=1,083 questions). b, Performance across question categories in GTEx cases assessed by the GPT-4o-based judger on answerable questions (n=274 questions). c, Performance of ALPaCA-Hybrid across TCGA cancer projects and GTEx tissue sites assessed by the GPT-4o-based judger on full open-ended test set (n=1,641 questions). d, Corresponding performance assessed by expert pathologists on the full open-ended test set (n=1,641 questions).

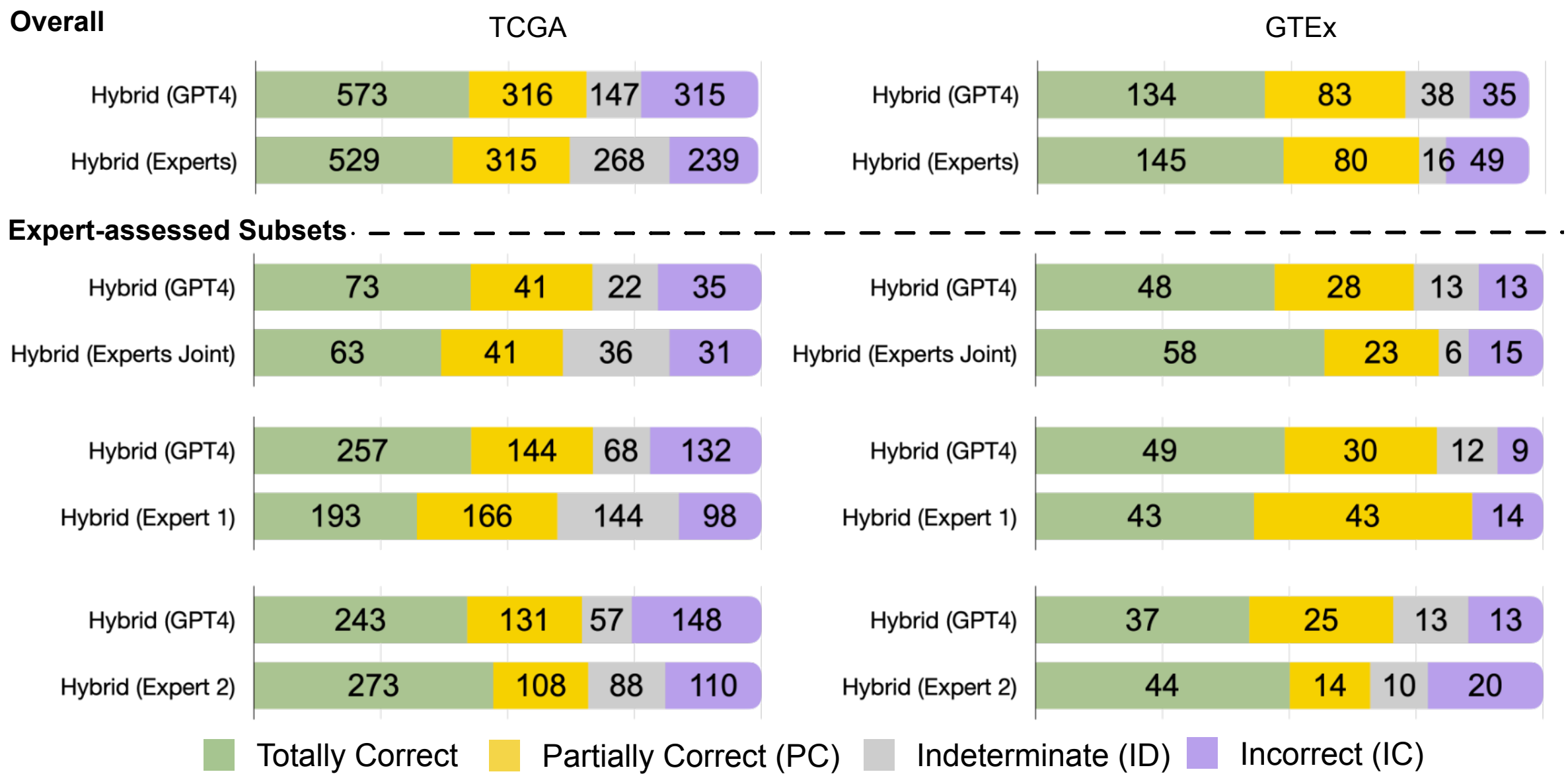

Supplementary Figure 4: Open-ended response evaluation comparison of ALPaCA-Hybrid between the GPT-4o-based judger and expert pathologists on the full open-ended test set of the General Slide-QA dataset, broken down by expert-assessed subsets for TCGA (n=1,351 questions) and GTEx (n=290 questions).

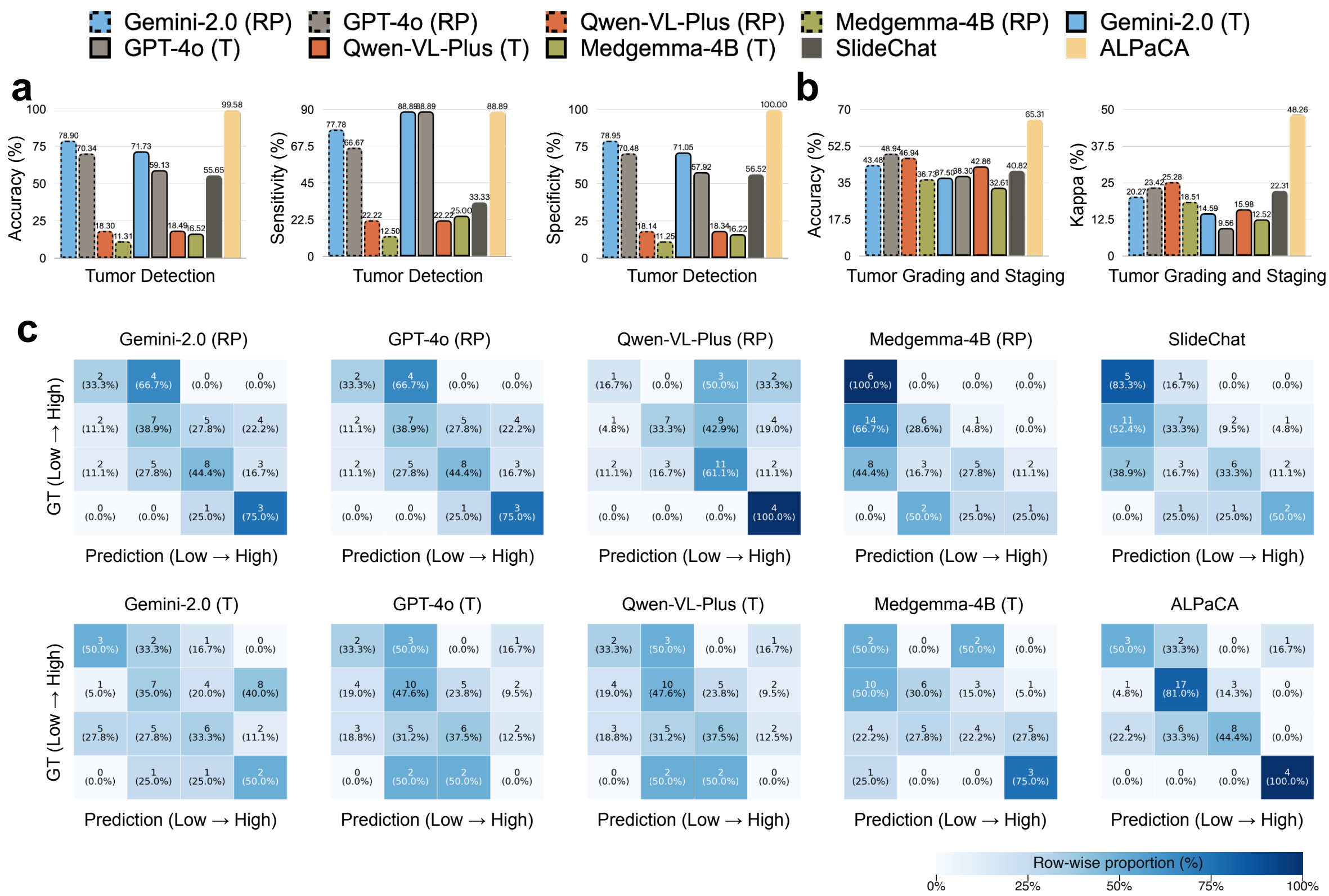

Supplementary Figure 5: Clinical-task evaluation comparing ALPaCA with LVLM baselines on the test set of General Slide-QA. a, Performance on close-ended tumour detection questions, reported using Accuracy, Sensitivity, and Specificity. This analysis includes the full tumour Detection subset (n=239), comprising both True/False items and multiple-choice QAs describing tumour vs non-tumour status. Binary labels were unified as tumour-positive vs tumour-negative; for multiple-choice items, labels were derived from option semantics. b, Performance on tumour grading and staging questions, reported using Accuracy and Cohen's kappa. This analysis includes clinically orderable grading/staging multiple-choice questions only (n=49). The tumour Grade and Staging category contains 79 close-ended QAs in total; we included only multiple-choice QAs with robust low-to-high ordering and excluded True/False QAs and non-orderable QAs. c, Row-normalized confusion matrices for tumour grading and staging. Cell annotations show count and row percentage. Classes were mapped to ordinal levels (Low to High) per question based on option semantics before computing agreement metrics.

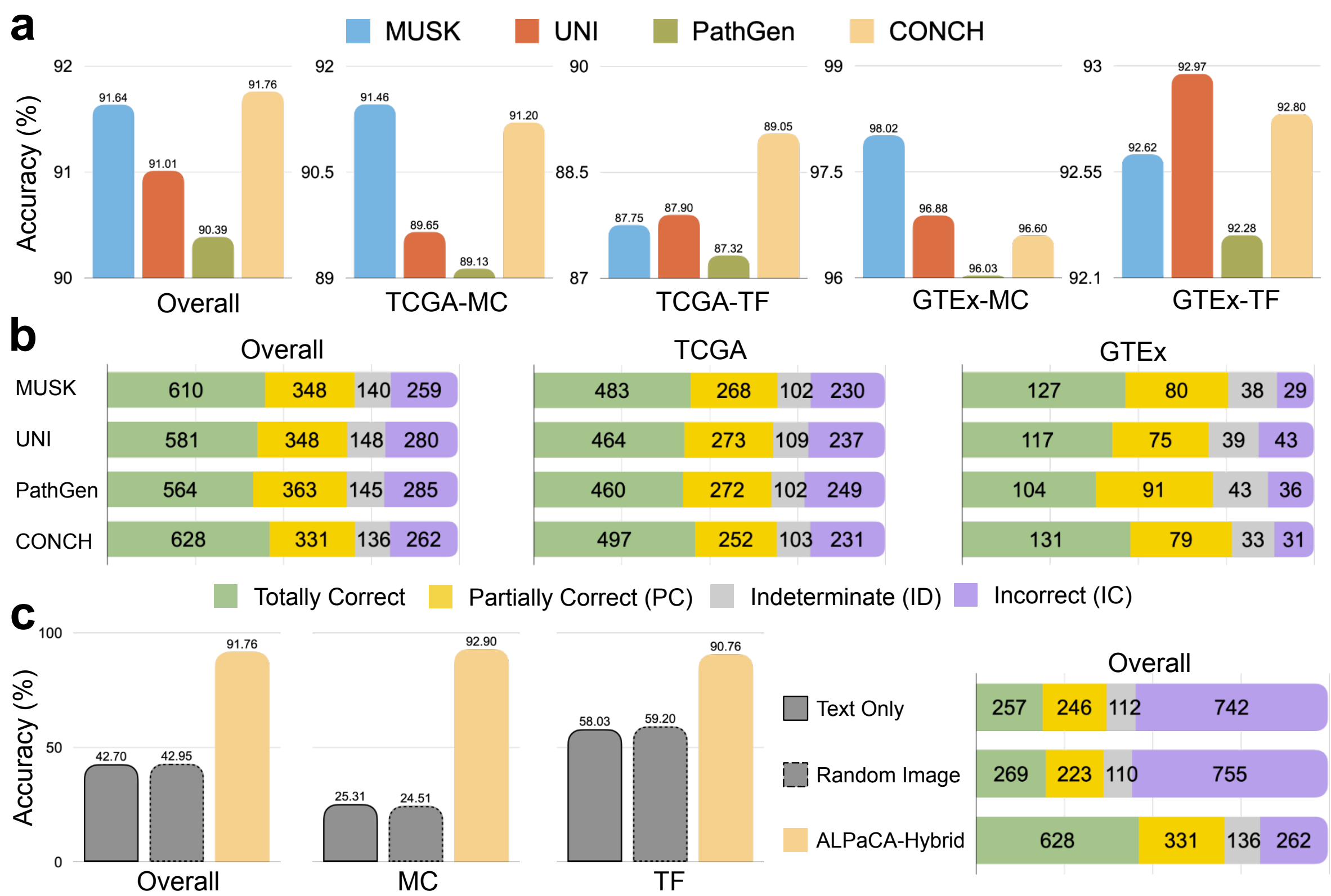

Supplementary Figure 6: Ablation studies of ALPaCA-Hybrid on the General Slide-QA test set. a, Close-ended performance obtained using different pathology foundation models as the patch encoder (n=2,403 questions). b, Open-ended performance obtained using different pathology foundation models as the patch encoder on answerable questions (n=1,357 questions). c, Performance of text-only and random-image baselines on close-ended questions (n=2,403 questions) and open-ended answerable questions (n=1,357 questions).

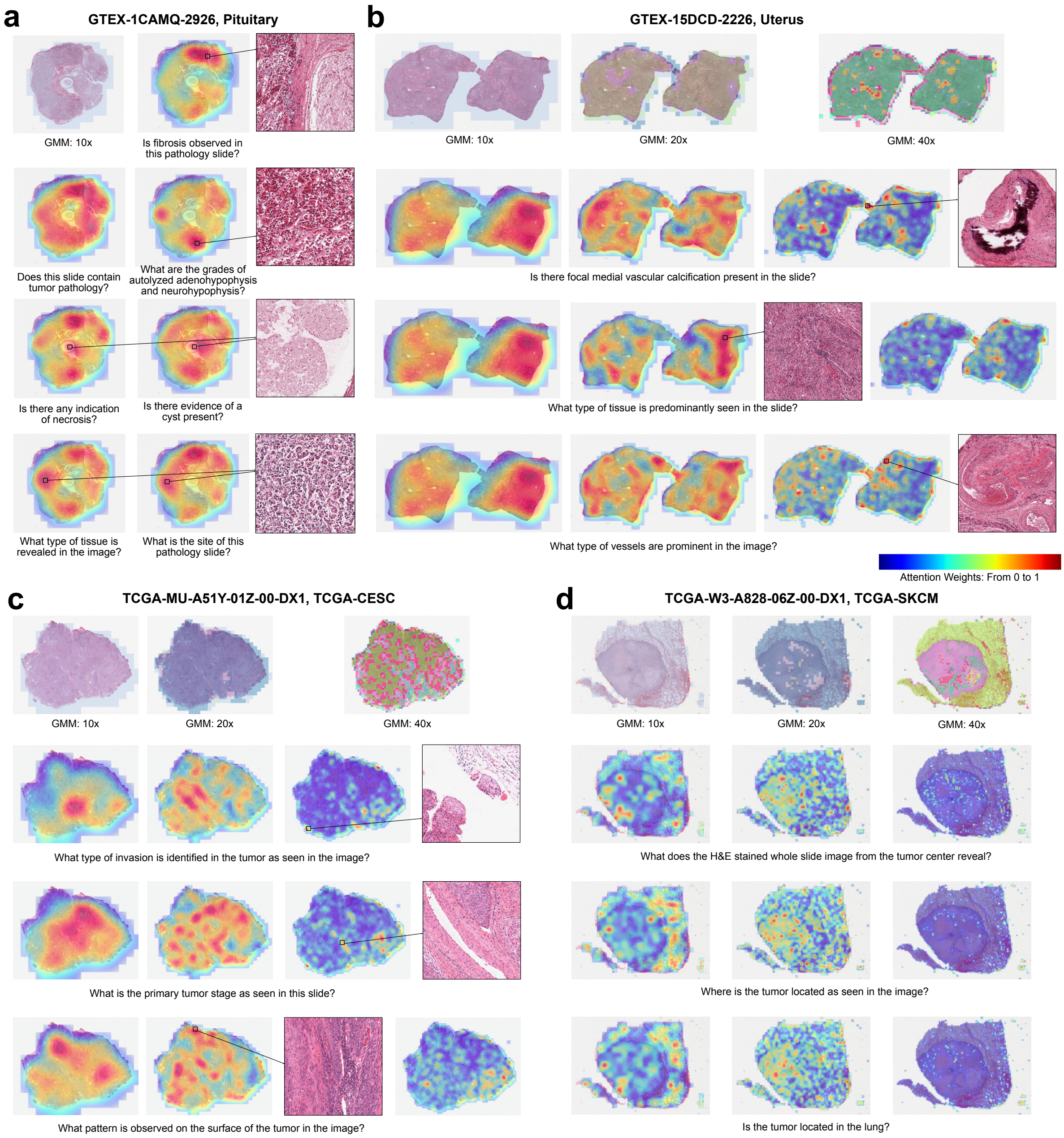

Supplementary Figure 7: Qualitative visualisation of ALPaCA-Hybrid on four General Slide-QA test cases. For each case, both GMM-based prototype maps and LongFormer-based attention maps are shown. a, Question-dependent LongFormer attention maps at the same magnification level for a representative case. b-d, Multi-scale visualisation across different magnification levels and question types for three additional cases. Colors in the GMM-based prototype maps denote slide- and magnification-specific prototypes and are not shared across maps.

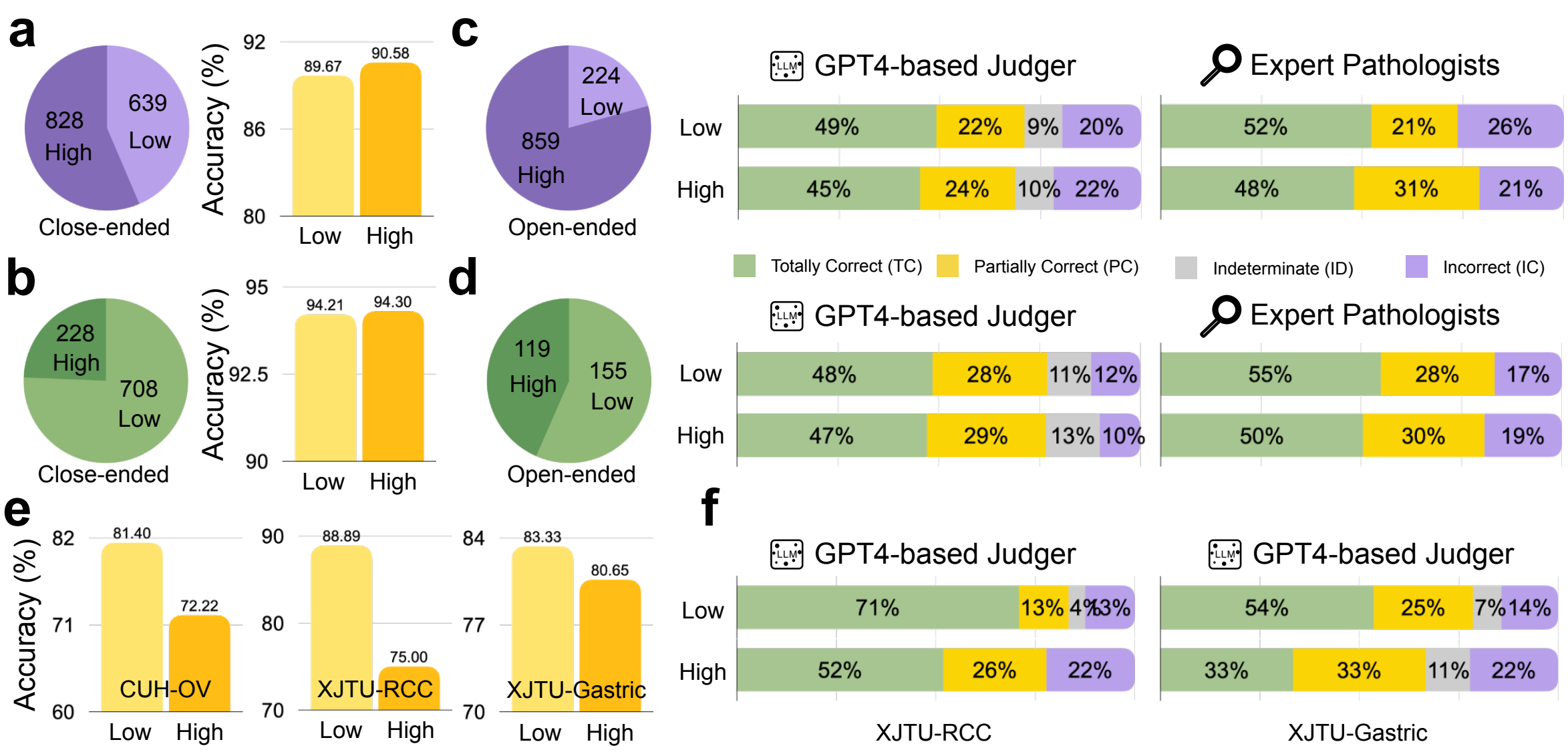

Supplementary Figure 8: Question distribution and performance comparison between low- and high-power questions for ALPaCA-Hybrid on the test set of General Slide-QA and external clinical cohorts. a, Close-ended questions in TCGA (low-power, n=639 questions; high-power, n=828 questions). b, Close-ended questions in GTEx (low-power, n=708 questions; high-power, n=228 questions). c, Open-ended answerable questions in TCGA (low-power, n=224 questions; high-power, n=859 questions). d, Open-ended answerable questions in GTEx (low-power, n=155 questions; high-power, n=119 questions). e, Close-ended questions in CUH-OV (low-power, n=43 questions; high-power, n=36 questions), XJTU-RG renal cell carcinoma (low-power, n=27 questions; high-power, n=24 questions) and XJTU-RG gastric cancer (low-power, n=18 questions; high-power, n=31 questions). f, Open-ended questions in XJTU-RG renal cell carcinoma (low-power, n=24 questions; high-power, n=27 questions) and XJTU-RG gastric cancer (low-power, n=28 questions; high-power, n=18 questions).

Project	Tissue Site	Diagnostic Type	Patient	Slide	Report/Note
TCGA	151	134	9,544	11,634	9,544
GTEx	40	-	971	24,279	24,279
TCGA-BRCA	6	22	1,046	1,110	1,046
TCGA-STAD	7	8	375	400	375
TCGA-KIRC	1	2	492	498	492
TCGA-OV	2	3	93	93	93
XJTU-RCC	1	3	10	11	-
XJTU-Gastric	1	3	10	10	-
CUH-OV04	6	5	10	21	-

Supplementary Table 1: Statistics of Source Data

Dataset	Data Source	Question Type	Number of Q&A Pairs		
			Train	Test	Total
General Slide-Caption	TCGA	Description	11,417	217	11,634
	GTEx	Description	24,159	120	24,279
General Slide-QA	TCGA	Open-ended	60,441	1,351	61,792
	TCGA	Close-ended	79,670	1,467	81,137
	GTEx	Open-ended	47,017	290	47,307
	GTEx	Close-ended	149,879	936	150,815
General Slide-QA (XJTU-RG)	XJTU-RCC,XJTU-Gastric	Open-ended	-	97	97
	XJTU-RCC,XJTU-Gastric	Close-ended	-	100	100
General Slide-QA (CUH-OV)	CUH-OV04	Close-ended	-	79	79
Specific Slide-QA (Breast)	TCGA-BRCA	Open-ended	12,830	359	13,189
	TCGA-BRCA	Close-ended	17,000	475	17,475
Specific Slide-QA (Morphology)	TCGA-STAD	Open-ended	2,676	59	2,735
	TCGA-STAD	Close-ended	3,886	79	3,965
	TCGA-KIRC	Open-ended	3,694	28	3,722
	TCGA-KIRC	Close-ended	5,102	37	5,139
	TCGA-OV	Open-ended	581	18	599
	TCGA-OV	Close-ended	916	24	940

Supplementary Table 2: Statistics of Curated Datasets

Categories	Example Questions
tumour Origin and Localization	Where is the tumour located as seen in the image?<br>- A. In the breast. B. In the lung. C. In the skin. D. In the liver.<br>Which organ is the origin of the tumour seen in the image?<br>- A. Stomach. B. Lung. C. Kidney. D. Liver.<br>Is the location of the tumour in the cervix uteri?<br>Is the primary site of the tumour located in the breast?
tumour Diagnosis and Classification	What type of tumour is observed in the image?<br>- A. Mixed germ cell tumour. B. Squamous cell carcinoma. C. Melanoma. D. Adenocarcinoma.<br>What type of neoplasm is primarily seen in the image?<br>- A. Meningioma. B. Neuroblastoma. C. High-grade glioblastoma. D. Low-grade glial neoplasm.<br>Is the tumour identified as malignant epithelioid mesothelioma?<br>Is the primary diagnosis a nodular melanoma?
Tissue-level Histological Features	What histological feature is noted in the tumour?<br>- A. Bone marrow infiltration. B. Fibrous tissue. C. Cartilage formation. D. Necrosis.<br>What type of appearance does one of the lesions in the image have?<br>- A. Ulcerated appearance. B. Papillary appearance. C. Flat appearance. D. Cystic appearance.<br>Is there evidence of necrosis in the image?<br>Are psammoma bodies visible in the image?
Cell-level Histological Features	What type of cells predominantly compose the tumour in the image?<br>- A. Fibroblasts. B. Squamous cells. C. Lymphocytes. D. Epithelioid cells.<br>Which cellular feature is NOT observed in the image?<br>- A. Plasma cells. B. Cartilage cells. C. Myoblasts. D. Squamous cells.<br>Are there glial cells with oligodendroglial features seen in the image?<br>Does the image show evidence of nuclear atypia?
tumour Grade and Staging	What is the pathologic stage of the tumour in the image?<br>- A. pT2. B. pT4. C. pT3. D. pT1.<br>What is the Nottingham Grade of the tumour seen in the image?<br>- A. Grade 2. B. Grade 1. C. Grade 3. D. Grade 4.<br>Is the tumour characterized by a high mitotic rate?<br>Does the image show high-grade malignant features?
Invasion Characteristics	What type of invasion is noted in the tumour as seen in the image?<br>- A. Vascular invasion. B. Lymphatic invasion. C. Perineural invasion. D. Transcapsular invasion.<br>What does the tumour invade according to the image?<br>- A. Bone. B. Blood vessels. C. Pleura and mediastinal fat. D. Skin.<br>Is there evidence of vascular invasion in the image?<br>Does the image show lymphovascular invasion?
tumour Micro Environment	What notable feature is observed in the surrounding kidney tissue in the image?<br>- A. Hyperplasia. B. Inflammation. C. Glomerulosclerosis. D. Fibrosis.<br>What type of changes are noted in the image?<br>- A. Metaplastic changes. B. Inflammatory changes. C. Necrotic changes. D. Proliferative fibrocystic changes.<br>Is there evidence of necrosis and fibrosis in the image?<br>Is there chronic inflammation observed in the image?
tumour Growth Patterns	What pattern is observed on the surface of the tumour in the image?<br>- A. Solid pattern. B. Papillary pattern. C. Necrotic pattern. D. Cystic pattern.<br>What is the predominant growth pattern of the tumour?<br>- A. Solid. B. Cystic. C. Sarcomatoid. D. Tubulopapillary.<br>Does the tumour exhibit a cribriform growth pattern?<br>Is the tumour well-circumscribed as seen in the image?
General Microscopic Examination	What additional findings are noted in the image?<br>- A. Chondrosarcoma. B. Hemangioma. C. Osteosarcoma. D. Atypical lobular hyperplasia.<br>What type of staining was used for the whole slide images?<br>- A. Giemsa staining. B. PAS staining. C. H&E staining. D. Silver staining.<br>Is the histological examination based on H&E staining?<br>Is the carcinoma clear from all margins microscopically?

Supplementary Table 3: Example close-ended questions of different categories in General Slide QA dataset (TCGA).

Categories	Example Questions
Organ Identification	Which site is represented in the pathology slide?<br>- A. Lymph node. B. Tibial nerve. C. Brain tissue. D. Bone marrow.<br>Which organ is represented in the pathology slide?<br>- A. Lung. B. Heart. C. Liver. D. Kidney.<br>Is the site of the slide from the esophagus?<br>Is the location of the pathology slide in the bladder?
Tissue Structure	What type of tissue is predominantly observed in the image?<br>- A. Fibrovascular tissue. B. Prostatic tissue. C. Melanoma tissue. D. Neural tissue with adipose.<br>What condition is observed in the adipose tissue in this image?<br>- A. Necrosis. B. Hyperplasia. C. Hypertrophy. D. Atrophy.<br>Is there visible muscularis in the slide?<br>Is there any indication of fibrous tissue in the image?
Cellular Morphology	What type of cells are predominantly seen in this testis pathology?<br>- A. Fibroblasts. B. Leydig cells. C. Epithelial cells. D. Muscle cells.<br>What changes are seen in Purkinje cells according to the image?<br>- A. Necrotic degeneration. B. Hypoxic damage. C. tumoural enlargement. D. Fibrotic changes.<br>Are degenerated myocytes visible in the image?<br>Are there pigmented macrophages visible in the image?
Immune System	What type of immune cells are noted in the pathology?<br>- A. Basophils. B. Eosinophils. C. Neutrophils. D. Lymphocytes.<br>What does the image indicate about the severity of the inflammation?<br>- A. Severe necrosis. B. No inflammation. C. Chronic fibrosis. D. Mild-moderate inflammation.<br>Is there evidence of inflammation in the pathology slide?<br>Is there evidence of fibrosis observed in the image?
Vessels and Circulation	What is the condition of the vascular structures seen in the image?<br>- A. Congested capillaries. B. Numerous intact arteries.<br>- C. Several thrombosed arteries. D. Only veins present.<br>What is noted about the stromal vessels in the image?<br>- A. They are thin-walled and collapsed. B. They are congested, thick-walled.<br>- C. They are absent. D. They are invaded by tumour cells.<br>Is there evidence of congestion seen in the image?<br>Are vessels prominently observed in the slide?
Sample Quality	What is observed regarding the epithelial preservation in the slide?<br>- A. Well preserved in all pieces. B. Completely destroyed.<br>- C. Well preserved in some pieces. D. Exhibiting necrosis.<br>What is the condition of the specimen as seen in the image?<br>- A. Poorly fixed with separation and fragmenting. B. Well fixed with clear margins.<br>- C. Completely intact with no issues. D. Freezing artifacts present.<br>Is the tissue sample large enough to assess detailed features?<br>Is the overall quality of the section poor?
Other Findings	What is the primary pathology observed in this slide?<br>- A. tumour growth. B. Necrosis. C. Cellular mitosis. D. Fibrosis.<br>What type of condition is observed in the slide?<br>- A. Colonic adenoma. B. Squamous cell carcinoma. C. Hashimoto thyroiditis. D. Normal thyroid function.<br>Is atherosis present in the image?<br>Are there large infarcts visible in the image?

Supplementary Table 4: Example close-ended questions of different categories in General Slide QA dataset (GTEx).

Categories	Example Questions
tumour Origin and Localization	Where is the tumour present microscopically in this slide?<br>Where does the tumour originate from according to this slide?<br>What is the original site of the tumour as seen in this slide?<br>From which tissues does the tumour in this slide originate?<br>From which part of the lung does the carcinoma in this slide originate?
tumour Diagnosis and Classification	What type of tumour is identified in this slide?<br>What is the primary diagnosis of the tumour described in this slide?<br>How is the tumour diagnosed in terms of its histological type?<br>What subtype of carcinoma is mentioned in this slide?<br>How is the carcinoma specifically classified in this slide?
Tissue-level Histological Features	What is the histological composition of the tumour?<br>How does the tumour appear histologically in this slide?<br>Are there any areas of necrosis in the tumour as seen in the slide?<br>What histological features are observed in the primary tumour's H&E slide?<br>What additional findings are present within the myometrium in the image?
Cell-level Histological Features	What nuclear features are observed in the cells in this slide?<br>What is the mitotic activity observed in this slide?<br>What cellular features are identified in this slide?<br>What are the characteristics of the epithelial cells seen in the image?<br>Does the tumour in this slide exhibit any mitotic activity?
tumour Grade and Staging	What is the stage classification of this slide?<br>What histological grade is the tumour in this slide?<br>What is the nuclear grade of the tumour in this slide?<br>What histologic grade is assigned to the tumour seen in this slide?<br>What FIGO grade is the tumour classified as?
Invasion Characteristics	Which structures does the tumour invade as seen in this slide?<br>What tissues does the tumour invade in this slide?<br>What layers are invaded by the tumour as seen in this slide?<br>Is perineural invasion present in the melanoma as seen in this slide?<br>Does the tumour show any invasion in this slide?
tumour Micro Environment	What type of reaction is observed around the tumour in this slide?<br>What kind of inflammatory response is induced by the tumour in this slide?<br>What is the level of lymphocytic infiltrate in the tumour in this slide?<br>What is found in the surrounding tissues of the tumour in this slide?<br>What evidence of tissue reaction is seen in this slide?
tumour Growth Patterns	What growth patterns are observed in the tumour in this slide?<br>What histological growth pattern is observed in this slide?<br>What pattern do the tumour cells exhibit in this slide?<br>What components make up the tumour seen in this slide?<br>What is the composition of the stroma in the tumour?
General Microscopic Examination	What notable features are seen in this slide?<br>What evidence of pathology is shown in this slide?<br>What components does this slide consist of?<br>What staining technique is used in the slide of the tumour?<br>What additional findings are present in this slide?

Supplementary Table 5: Example open-ended questions of different categories in General Slide QA dataset (TCGA).

Categories	Example Questions
Organ Identification	Which area of the cervix is highlighted in the image?<br>What part of the kidney is primarily observed in the image?<br>What anatomical structure is depicted in the image?<br>From what area of the body is the skin sample taken?<br>What type of brain tissue is shown in the image?
Tissue Structure	What type of stroma is observed in the prostate slide?<br>What histological features are observed in the pancreatic tissue?<br>What type of epithelium is observed in the image?<br>What structures are observed within the subcutaneous adipose tissue in the image?<br>What characteristic features are present in the atypical glands?
Cellular Morphology	What type of cell accumulation is present in the lung tissue?<br>Is there any evidence of dysplasia in the foveolar cells?<br>What type of cellular collections are seen in the outer fibrous areas?<br>What type of casts are present in the tubular structures?<br>What cellular components are present alongside the visceral adipose tissue?
Immune System	What type of infiltrate is present in the liver slide?<br>What surrounds the necrotic areas in the pancreas?<br>What is indicated by the presence of interstitial fibrosis?<br>What type of inflammation is observed in the salivary gland elements?<br>What type of infiltrate is present in the peribronchial area?
Vessels and Circulation	What type of vessels are prominent in the image?<br>What type of vascular condition is observed in the aorta artery?<br>What type of sclerosis is observed in the vessels?<br>What condition is indicated by the appearance of the stromal vessels?<br>What type of vascular structures are observed in the spleen pathology slide?
Sample Quality	What is the condition of the specimens seen in the image?<br>What is noted about the technical quality of the slide?<br>What artifacts are present in the image?<br>How is the tissue quality described in the slide?<br>What degree of autolysis is present in the muscle observed in the slide?
Other Findings	What type of nephrosclerosis is observed in the kidney cortex?<br>What type of myocarditis is observed in the image?<br>What is observed in the portal areas of the liver slide?<br>What condition is observed in the Tibial Artery?<br>What additional components are seen in the testis pathology slide?

Supplementary Table 6: Example open-ended questions of different categories in General Slide QA dataset (GTEx).

Datasets/Types	Example Questions
Breast/Close-ended	What is the level of inflammation noted in the image?<br>- A. Absent. B. Moderate inflammation observed.<br>- C. Mild inflammation detected. D. Severe inflammation present.<br>What is the PAM50 and Claudin-low molecular subtype observed in the image?<br>- A. Normal-like. B. LumA. C. Basal-like. D. LumB.<br>Is there any evidence of lymphovascular invasion in the image?<br>Is there necrosis present in the image?
Breast/Open-ended	What is the PAM50 and Claudin-low molecular subtype of the breast cancer?<br>What is the score for mitosis in the breast cancer?<br>What is the nuclear pleomorphism score for the breast cancer?<br>What type of fibrous focus is present in the breast cancer?
Morphology/Close-ended	What type of cells predominantly feature in the slide?<br>- A. Spindle-shaped cells embedded in fibrous stroma B. Columnar cells with goblet cells.<br>- C. Squamous cells in clusters D. Round cells with abundant cytoplasm<br>What type of inflammatory changes are observed in the non-neoplastic sections?<br>- A. Chronic inflammation with lymphocytic infiltration B. Granulomatous inflammation with giant cells<br>- C. Acute inflammation with neutrophils D. Fibrinous exudate.<br>Is there a notable increase in mitotic activity indicating rapid proliferation?<br>Are there any signs of ulceration visible in the image?
Morphology/Open-ended	What evidence of acute inflammation is noted in the slide?<br>What type of stromal reaction is associated with the carcinoma in the slide?<br>What are the characteristics of the nuclei in the tumour cells?<br>What indicates the aggressive behavior of the tumour in the image?

Supplementary Table 7: Example questions of two Specific Slide QA datasets.

Case ID (Correction)	Question	Answer (GT)	Response (ALPaCA-Hybrid)	Explanation from Pathologists
TCGA-97-7937 (IC ->TC)	What is the primary diagnosis of the tumour in this slide?	The primary diagnosis is micropapillary carcinoma.	The primary diagnosis is adenocarcinoma originating from the lung.	Micropapillary carcinoma is a type of adenocarcinoma. The tumour is actually described in the report as being mixed consisting of micropapillary (70%), acinar (10%), lepidic (10%) and giant cell (10%) components, so summarising as 'adenocarcinoma' in response to the question is appropriate.
TCGA-EW-A1P4 (IC ->TC)	What is the primary diagnosis of the tumour seen in this slide?	The primary diagnosis is medullary carcinoma of the breast.	The primary diagnosis is infiltrating duct carcinoma of the breast.	Medullary carcinoma is no longer recognised as specific carcinoma type within the WHO classification of tumours so by modern classification standards this would be classified as Invasive breast carcinoma of no special type (and infiltrating duct carcinoma is a commonly used synonym).
TCGA-EW-A2FV (IC ->PC)	Is ductal carcinoma in situ present in the slide, and if so, what grade?	Yes, ductal carcinoma in situ (DCIS) of high nuclear grade (DIN 3) is present.	Yes, ductal carcinoma in situ of intermediate grade is present in the image.	Grade is wrong, so partially correct.
TCGA-WE-A8JZ (IC ->TC)	What type of melanoma is identified in this slide?	A malignant melanoma of the nodular type with a vertical growth phase is identified in the image.	The image shows a malignant melanoma of the epithelial subtype.	Typing on the melanoma as a nodular type with vertical growth phase requires assessment of the primary lesion, but this lesion was in a lymph node so not possible to make that assessment. Some of the melanoma in the lymph node morphologically resembled metastatic carcinoma so referring to it as 'epithelioid' or 'epithelial-type' is appropriate
TCGA-XE-AANI (IC ->TC)	What cancer type is identified in the image?	The cancer type identified is teratocarcinoma.	The cancer type identified is a mixed germ cell tumour.	The suggested diagnosis was I think based on the ICD-O coding written on the pathology report, but the pathology report text actually gave the diagnosis as 'Mixed malignant germ cell tumour'.

Supplementary Table 8: Examples of QA from the General Slide-QA dataset in the Tumour Diagnosis and Classification category, where expert pathologists corrected the judgments made by the GPT-4o-based judger and provided detailed explanations.

Supplementary Note 1: Patch encoders

To examine how the choice of patch-level encoder affects downstream slide-level QA performance, we evaluated ALPaCA using three alternative pathology foundation models: MUSK, UNI, and PathGen, in addition to the default CONCH encoder.

PathGen and MUSK are vision–language pathology foundation models, similar to CONCH, whereas UNI is a vision-only foundation model. UNI and MUSK adopt the larger ViT-L/16 backbone, whereas PathGen uses a ViT–B/16, similar to CONCH. Previous studies report that these pathology foundation models achieve comparable performance on patch-level classification and retrieval tasks, with PathGen generally exhibiting slightly lower representational strength.

By substituting these pathology foundation models as patch encoders of ALPaCA, we assessed whether higher-capacity encoders translate into measurable gains in slide-level QA, whether vision–language foundation models offer advantages over vision-only foundation models for multimodal reasoning, and whether ALPaCA's performance remains robust to variation in the underlying patch-level feature extractor. For a controlled comparison, all experiments were conducted using the ALPaCA-Hybrid architecture, with only the patch encoder substituted.

Across the close-ended QA evaluations (Supplementary Fig. 6a), ALPaCA-Hybrid exhibited strong robustness to the choice of patch encoder: all encoders except PathGen achieved overall accuracies above 91%, indicating that when the underlying patch representations are of broadly comparable quality, downstream slide-level QA performance varies only modestly. Among the alternatives, MUSK and CONCH achieved highly similar performance, while UNI performed slightly worse overall, suggesting that vision–language foundation models provide a modest advantage over vision-only encoders. However, the gap between vision–language and vision-only foundation models was substantially smaller than the performance drop associated with reduced model capacity, as reflected by the consistently lower accuracy of PathGen.

For open-ended QA (Supplementary Fig. 6b), we observed a broadly similar pattern. Using the GPT-4o-based judger, ALPaCA-Hybrid with MUSK or CONCH achieved the highest overall correctness (TC+PC), with UNI performing slightly worse but still close, and PathGen yielding the weakest results. Notably, for PathGen the main deficit lay in a reduced proportion of TC predictions, whereas its correctness rate (TC+PC) remained comparable to the other encoders, indicating that responses were often partially correct rather than entirely incorrect. Moreover, no consistent systematic differences emerged between TCGA and GTEx subsets across encoders.

Overall, these patterns suggest that encoder capacity is more critical than modality alignment for slide-level QA: once a reasonable pathology foundation model is used, ALPaCA-Hybrid is largely insensitive to whether the encoder is vision–language or vision-only, but degrades more noticeably when encoder capacity is reduced.

Supplementary Note 2: Baseline controls

To isolate the contribution of visual information in slide-level QA, we incorporated two baseline controls. First, a text-only baseline was constructed by providing ALPaCA-Hybrid with only the slide-level question or prompt and no image-derived input, allowing us to quantify the extent to which performance can be achieved without any visual evidence. Second, a random-image baseline was designed to test whether generic or distribution-matched visual tokens could inadvertently boost performance. Because natural images differ substantially from histopathology slides, we instead generated pseudo-visual tokens by sampling from the empirical mean and covariance of WSI embeddings within each batch, matching token scale and distributional statistics while removing all meaningful histological content. Together, these baselines serve to determine (1) whether ALPaCA relies on visual information and (2) whether its performance could be explained by non-specific visual patterns rather than genuine slide-level reasoning.

Across both close-ended and open-ended QA tasks (Supplementary Fig. 6c), the text-only and random-image baselines performed substantially worse than models given real slide information, demonstrating that neither question semantics alone nor generic visual tokens contain enough signal to answer pathology questions meaningfully. For close-ended QA, both baselines reached only 42–43% overall accuracy (MC: 24–25%, TF: 58–59%), which approaches the performance expected from chance-level guessing in multiple-choice formats and provides only minimal signal gain for true/false questions. For open-ended QA, GPT-4o-based evaluations showed that the vast majority of baseline responses were incorrect (*e.g.*, text-only: 742 IC; random-image: 755 IC), in contrast to the substantially higher proportion of semantically correct outputs when real slide features were provided (262 IC).

Supplementary Note 3: Visualisation

To better characterise how ALPaCA-Hybrid processes slide information, we performed qualitative visualisation analyses (Supplementary Fig. 7) based on its two key vision components: GMM and LongFormer-based slide adaptors. Together, these complementary views illustrate how the model organises morphological patterns and how it retrieves question-relevant visual cues during inference.

The GMM adaptor provides a global, question-independent representation of each slide. By assigning each patch to the prototype with the highest probability, we obtain prototype maps that reflect how the model partitions the slide into visually coherent regions across magnification levels. While the prototypes are not bound to predefined semantic labels, they reveal the structural distinctions that the model internally recognises, highlighting broad differences between stromal, epithelial, necrotic, or other tissue patterns. These maps provide an overview of the model's baseline morphological understanding before question-specific reasoning occurs.

The LongFormer adaptor, in contrast, captures the question-conditioned interaction

between visual evidence and question context. We extracted the final-layer attention weights across all heads, normalised them, and aggregated them to generate patch-level attention maps for each magnification level. These maps indicate which regions the model emphasises when formulating an answer to a given question, showing how fine- and coarse-resolution features are selectively integrated depending on the diagnostic task.

In Supplementary Fig. 7a, we present question-conditioned LongFormer attention maps at a fixed magnification level (10×) for a representative case. For a global question such as tumour presence, attention is broadly distributed across the tissue, consistent with the need to assess overall slide-level morphology in a normal specimen. In contrast, more specific questions induce relative shifts in attention, with increased weighting of morphologically relevant regions compared with other queries, reflecting question-dependent reweighting of visual evidence. We further illustrate how attention patterns vary jointly across question types and magnification levels in Supplementary Fig. 7b–d. Overall, the GMM prototype maps reveal increasingly rich structural detail at higher magnifications, particularly at 40×, which is accompanied by comparatively sparser and more selective LongFormer attention patterns at this scale. In Supplementary Fig. 7b, attention maps at 10× show limited differentiation across questions, reflecting the dominance of global contextual cues at low magnification. At 20×, tissue-related questions elicit more focused attention on representative tissue regions relative to other queries. For the question concerning vascular calcification, the 40× attention map concentrates on the corresponding vascular structure, consistent with the fine-grained morphological evidence required. Similarly, for questions regarding prominent vessel types, attention at 40× extends over a broader set of vascular regions than for other question categories. A similar trend is observed in Supplementary Fig. 7c. At 40× magnification, attention for the lymphovascular invasion question highlights focal regions consistent with suspected infiltrative areas. For the tumour staging question ("The primary tumour is staged as pT2a"), although the stage cannot be definitively determined from the slide alone, the model assigns increased attention at 40× to regions containing thick-walled vessels, a histological feature commonly associated with deeper cervical involvement. For questions related to tumour growth patterns, attention at 20× shifts toward papillary structures located in the upper portion of the slide. In Supplementary Fig. 7d, although the questions are relatively simple, distinct differences in attention allocation are still evident. For the two slide-location questions, attention at 10× is preferentially distributed over the right lung tissue region, indicating reliance on low-magnification contextual cues. In contrast, for the tumour-related question, attention shifts toward the tumour region at 40×, reflecting increased reliance on higher-magnification features compared with location-based queries.

It is important to note that, owing to the relatively dense visual tokenisation (32/16/8 tokens across magnification levels) and the absence of explicit sparsity or localisation constraints in the vision adaptor, attention is not expected to concentrate on a small number of discrete regions. Instead, the model retains a broad contextual representation of the slide while modulating relative attention weights across regions in a question-dependent manner. Accordingly, the attention maps should be interpreted comparatively

across different questions, reflecting how diagnostic intent biases the integration of visual information rather than providing strict spatial localisation.

Supplementary Note 4: Low- vs high-power question analysis

In routine diagnostic practice, pathologists switch between low-power (overview) and high-power (cellular-level) examination based on the specific diagnostic task. To examine how ALPaCA's behaviour varies with magnification requirements, we compared its performance on questions answerable at low power versus those requiring high-power visual detail. For this analysis, a pathologist reviewed all close-ended and open-ended questions in the test set of General Slide-QA and annotated based on the diagnostic intent of each question, whether it would typically require high-magnification inspection or could be addressed using low-magnification contextual features only.

The distribution of low- versus high-power questions, along with ALPaCA-Hybrid's corresponding close-ended and open-ended performance on TCGA and GTEx, is shown in Supplementary Fig. 8a-d. TCGA contains a larger proportion of high-power questions, consistent with tumour-related diagnostic tasks, such as grading, invasion, and cytological atypia, that typically require high-magnification review. In contrast, GTEx questions are mostly low-power, reflecting the normal-tissue notes from which they are derived and their emphasis on global architectural features. Across both datasets, open-ended questions include a greater fraction of high-power items than close-ended ones, as they more frequently probe descriptive or fine-grained histological details requiring cellular-level examination, whereas close-ended queries tend to target broader slide-level attributes.

For close-ended QA, ALPaCA-Hybrid performed consistently on low- and high-power questions in both TCGA and GTEx, with only marginal differences ($<1\%$) in accuracy (Supplementary Fig. 8a,b). In contrast, open-ended QA showed clear magnification-related effects (Supplementary Fig. 8c,d). Under the GPT-4o judger, low-power questions achieved slightly higher "totally correct" rates than high-power questions, and this gap widened in the expert evaluation (TCGA: 52% vs. 48%; GTEx: 55% vs. 50%). This pattern reflects the nature of high-power questions, which rely on fine-grained cellular and subcellular details, nuclear atypia, specific cytological patterns, or subtle tumour–stroma interactions, that slide-level LVLMs may struggle to represent fully.

We further extended this analysis to the independent clinical cohorts (XJTU-RG, and CUH-OV) to assess robustness under domain shift (Supplementary Fig. 8e,f). Across all external cohorts, a consistent pattern emerged: low-power questions exhibited greater robustness than high-power questions, for both close-ended and open-ended QA. For close-ended QA (Supplementary Fig. 8e), ALPaCA-Hybrid maintained relatively high accuracy on low-power questions across all cohorts, whereas performance dropped more noticeably for high-power questions, particularly in CUH-OV and XJTU-RG (Renal).

A similar but more pronounced trend was observed for open-ended QA (Supplementary Fig. 8f), where high-power questions showed a clear reduction in totally correct rates and an increase in partially correct or incorrect responses under the GPT-4o-based judger. This behaviour is expected under external validation: high-power questions are intrinsically more complex and heterogeneous, depending on fine-grained cellular cues that are sensitive to both domain shift and question-level ambiguity, whereas low-power questions rely on more stable global architectural features. Overall, ALPaCA-Hybrid maintains reliable performance on low-power questions in both close-ended and open-ended settings, while high-power questions highlight the primary challenge for slide-level LVLMs.

Supplementary Note 5: Prompts

All prompts used for data curation and result evaluation are provided as follows:

- TCGA Report Summary (GPT3.5 Turbo): *As a pathologist, please summarize the pathology report in one description paragraph. Content related to pathological examination must be retained as much as possible, and gross examination content is not required. If can not find any related content, return TBD.*
- TCGA Report Refinement (GPT4o): *Imagine you are a pathologist looking at a primary tumour slide, you only know it is from which organ. To describe H&E stained WSI from the tumour center, please use the information from this pathology report. Note that the information here might involve multiple slides, you need to distinguish which of them can be observed from one single primary tumour slide only. Don't mention any information that cannot be obtained from the information of the primary tumour slide. Don't mention any information on metastasis, tumour measurements, tumour size, lymph node metastasis, tumour margins, and gross examination. The description should identify the original site, cancer type, specific subtype, stage (which system, like AJCC), and grade. Also include relevant descriptions from the primary tumour's H&E slide, if it exists. Do not describe anything that does not exist in the provided text, and summarize it into one paragraph.*
- TCGA Open-ended Q&A Generation (GPT4o): *As a senior pathologist. You are given a text where a pathologist is describing a histopathology image. You are given the summary of a pathology slide. Generate well-defined question/answer pairs from these sentences. Consider the following requirements to generate questions and answers: - Only generate questions about information being seen in the image. - Do not generate questions from the context if the question is not explicitly asked. - Do not generate questions/answers based on information that can not be seen in the image being described like the patient's age, gender, medical history, or other studies/images outside of the current case. - If the text does not explicitly provide the answer to a question, do not generate a question/answer pair. - Do not answer the questions from information outside of the given text. - Do not use phrases like "mentioned", "suggests" or "text" in the answers. Instead, refer to*

the information as being seen "in the image". - Do not reveal answers to the questions. In the returned JSON file, use key input to represent the question use the key output to represent the answer, and use qas as the key of a series of QA pairs.

- TCGA Close-ended (multi-choice) Q&A Generation (GPT4o): *You are a senior pathologist. You are given a text where a pathologist is describing a histopathology image. You are given the summary of a pathology slide. Generate concise multiple choice question answer pairs from the provided pathology slide summary. If there is enough information, you should at least ask the location or site of this image. When generating question-answer pairs, consider the following requirements: - Do not answer the questions as if you are reading a report but as if you are looking directly at the pathology slide. - Do not generate questions that need to be answered with numbers like scores or calculations. - Do not include right or left direction in any question and answer since you can not tell direction from the local image. - The key question should include the question. The key choices should include four choices. Each choice should be apparently diverse and contain a different format or pattern. Two choices should be apparently wrong with different formats. One choice should be similar to the correct answer. The key answer should include the correct choice. Provide choices with the numbers A, B, C, and D. Provide an answer with the proper number and the answer content. - In the generated choices, two of the choices should be apparently wrong with apparently different patterns. One choice should have a similar pattern with the correct answer serving as the confusing choice. - Do not generate questions/answers based on information that can not be seen in the image being described like the patients' age, gender, medical history, or other studies/images outside of the current case. - Do not answer the questions using information outside of the given text. - Do not use phrases like "mentioned", "suggests" or "text" in the answers. Instead, refer to the information as being seen "in the image". In the returned JSON file, use the key question to represent the question and four choices. Use the key answer to represent the corresponding answer and use qas to include a series of QA pairs. Don't forget to include two apparently irrelevant choices. Here is an example format 'qas':['question':'What is the colour of the leaf?' 'choices': 'A. Green. B. Red. C. Yellow. D. Bule','answer':'A. Green.'].*

- TCGA Close-ended (true or false) Q&A Generation (GPT4o): *You are a senior pathologist. You are given a text where a pathologist is describing a histopathology image. You are given the summary of a pathology slide. Generate yes-no question-answer pairs from the provided pathology slide summary. Consider the following requirements to generate questions and answers: - Do not answer the questions as if you are reading a report but like you are looking directly at the pathology slide. - Do not generate questions that are related to numbers like scores or calculations. - Change affirmation or denial tone to make sure more questions should be answered with 'No'. - The key question should include the question. The key answer should be only yes or no. Any other keys are not needed - Do not generate questions/answers based on information that can not be seen in the image being described like the patients' age, gender, medical history, or other stud-*

ies/images outside of the current case. - Do not answer the questions using information outside of the given text. - Do not use phrases like "mentioned", "suggests" or "text" in the answers. Instead, refer to the information as being seen "in the image". - Do not reveal answers to the questions. In the returned JSON file, use the key question to represent the question. Use the key answer to represent the corresponding answer and use qas to include a series of QA pairs. Here is an example format 'qas':['question':'Is the colour of the leaf green?' 'answer':'Yes'] The summary content is as follows: content

- GTEx Notes Rephrasing (GPT4o): *Generate a one-sentence short descriptive report caption summarizing the key findings of a pathology assessment on the specific tissue, incorporating the major diagnostic categories related to the tissue condition, and providing a brief summary of the notable pathology information. Do not come to hypothetical conclusions. Avoid providing content that can not be found in the original report. The provided report is from a non-tumour pathology slide. Do not include information about 'pieces', 'length', 'area', 'thickness', and other quantitive information. Generate the caption in a descriptive report tone like this slide is something; this slide reveals something. Rephrase the returned answer to remove the hyphen in the tissue column. In the returned JSON file, only one key called the 'caption' value should be the generated caption.*

- GTEx Open-ended Q&A Generation (GPT4o): *As a senior pathologist, you are tasked with generating question/answer pairs based on the descriptions provided in a histopathology image summary. Please adhere to the following guidelines when formulating the questions and answers: 1. Limit the generation to two or three question/answer pairs per image caption, focusing on producing the most reliable pairs first. 2. Ensure that all captions and resultant question/answer pairs pertain exclusively to non-tumour pathology slides. 3. Questions should only pertain to specific, observable details in the image; avoid vagueness such as using terms like "notable." 4. Base questions solely on the content explicitly described in the image. Do not infer or assume additional information not presented. 5. Avoid creating question/answer pairs based on unobservable data such as the patient's demographic details, medical history, or external sources. 6. Formulate questions only if the text provides a clear answer; refrain from hypothesizing or assuming details not contained within the provided text. 7. In your answers, specify that details are "seen in the image," and avoid referencing the "text" or suggesting implications of the findings. 8. Craft questions in a way that they do not give away the answer. By following these guidelines, you will ensure that the question/answer pairs are accurate, specific, and strictly relevant to the given pathology slide images.*

- GTEx Close-ended (multi-choice) Q&A Generation (GPT4o): *You are an experienced pathologist who is good at generating multiple-choice question-answer pairs. The provided report is from a non-tumour pathology slide. You should at least ask about the location or site of this image and whether this slide is non-tumour or contains a tumour. When generating question-answer pairs, consider the following requirements: - Do not answer the questions as if you are reading a report but as if you are looking directly at the pathology*

slide. - Do not generate questions that need to be answered with numbers like scores or calculations. - Do not include right or left direction in any question and answer since you can not tell direction from the local image. - The key question should include the question. The key choices should include four choices. Each choice should be apparently diverse and contain a different format or pattern. Two choices should be apparently wrong with different formats. One choice should be similar to the correct answer. The key answer should include the correct choice. Provide choices with the numbers A, B, C, and D. Provide an answer with the proper number and the answer content. - In the generated choices, two of the choices should be apparently wrong with apparently different patterns. One choice should have a similar pattern with the correct answer serving as the confusing choice. - Do not generate questions/answers based on information that can not be seen in the image being described like the patients' age, gender, medical history, or other studies/images outside of the current case. - Do not answer the questions using information outside of the given text. - Do not use phrases like "mentioned", "suggests" or "text" in the answers. Instead, refer to the information as being seen in the image. In the returned JSON file, use the key question to represent the question and four choices. Use the key answer to represent the corresponding answer and use qas to include a series of QA pairs. Don't forget to include two apparently irrelevant choices. Here is an example format 'qas':['question':'What is the colour of the leaf?' 'choices': 'A. Green. B. Red. C. Yellow. D. Bule', 'answer': 'A. Green.'].

- GTEx Close-ended (true or false) Q&A Generation (GPT4o): *You are an experienced pathologist who is good at generating multiple-choice question-answer pairs. The provided report is from a non-tumour pathology slide. You should at least ask the location or site of this image and whether this slide is non-tumour or contains tumour.' When generating question-answer pairs, consider the following requirements: - Do not generate questions related to "pieces" in the clinical notes. - Generate the most clear and certain question pair. Do not generate questions that are similar to each other. - Do not answer the questions as if you are reading a report but as if you are looking directly at the pathology slide. - Do not generate questions that need to be answered with numbers like scores or calculation or counting results. - Do not include right or left direction in any question or answer since you can not tell direction from the local image. - Change affirmation or denial tone to make sure answer "Yes" and "No" are evenly distributed. - The key question should include the question. The key answer should be only yes or no. No other keys are needed - Do not generate questions/answers based on information that can not be seen in the image being described like the patients' age, gender, medical history, or other studies/images outside of the current case. - Do not answer the questions using information outside of the given text. - Do not use phrases like "mentioned", "suggests" or "text" in the answers. Instead, refer to the information as being seen "in the image". In the returned JSON file, use the key question to represent the question. Use the key answer to represent the corresponding answer and use qas to include a series of QA pairs. Here is an example format 'qas':['question':'Is the color of the leaf green?' 'answer':'Yes']*

The summary content is as follows: report

- TCGA-BRCA Open-ended Q&A Generation (GPT4o): *You will be given a Dict containing detailed information about breast cancer. Generate well-defined question/answer pairs from these key-value pairs. Try using only the original content and avoid adding hallucination. You can only make a little rephrase to avoid repetition. In the returned JSON file, use key input to represent the question and use key output to represent the answer and use qas as the key of a series of QA pairs. The summary content is as follows: content*

- TCGA-BRCA Close-ended (multi-choice) Q&A Generation (GPT4o): *You will be given a Dict containing detailed information about breast cancer. Generate well-defined question/answer pairs from these key-value pairs. Try using only the original content and avoid adding hallucination. You can only make a little rephrase to avoid repetition. When generating question-answer pairs, consider the following requirements: - Do not answer the questions as if you are reading a report but as if you are looking directly at the pathology slide. - Do not include right or left direction in any question and answer since you can not tell direction from the local image. - Do not generate questions and answers that should be answered with numbers like how many counting results. - The key question should include the question. The key choices should consist of four choices. Each choice should be apparently diverse and contain a different format or pattern. Two choices should be apparently wrong with different formats. One choice should be similar to the correct answer. The key answer should include the correct choice. Provide choices with the numbers A, B, C, and D. Provide an answer with the proper number and the answer content. - In the generated choices, two of the choices should be apparently wrong with apparently different patterns. One choice should have a similar pattern with the correct answer serving as the confusing choice. - Do not generate questions/answers based on information that can not be seen in the image being described like the patients' age, gender, medical history, or other studies/images outside of the current case. - Do not answer the questions using information outside of the given text. - Do not use phrases like "mentioned", "suggests" or "text" in the answers. Instead, refer to the information as being seen "in the image". In the returned JSON file, use the key question to represent the question and four choices. Use the key answer to represent the corresponding answer and use qas to include a series of QA pairs. Don't forget to include two apparently wrong choices. Here is an example format 'qas':['question':'What is the color of the leaf?' 'choices': 'A. Green. B. Red. C. Yellow. D. Bule','answer':'A. Green. '] The summary content is as follows: content*

- TCGA-BRCA Close-ended (true or false) Q&A Generation (GPT4o): *You will be given a Dict containing detailed information about breast cancer. Generate well-defined question/answer pairs from these key-value pairs. When generating question-answer pairs, consider the following requirements: - Generate the most clear and certain question pair. Generate at most 5 certain question-answer pairs for each summary. Do not generate*

questions that are similar to each other. - Do not answer the questions as if you are reading a report but as if you are looking directly at the pathology slide. - Do not generate questions that need to be answered with numbers like scores or calculation or counting results. - Do not include right or left direction in any question or answer since you can not tell direction from the local image. - Change affirmation or denial tone to make sure answers "Yes" and "No" are evenly distributed. - The key question should include the question. The key answer should be only yes or no. No other keys are needed - Do not generate questions/answers based on information that can not be seen in the image being described like the patients' age, gender, medical history, or other studies/images outside of the current case. - Do not answer the questions using information outside of the given text. - Do not use phrases like "mentioned", "suggests" or "text" in the answers. Instead, refer to the information as being seen "in the image". In the returned JSON file, use the key question to represent the question. Use the key answer to represent the corresponding answer and use qas to include a series of QA pairs. Here is an example format 'qas':['question':'Is the color of the leaf green?' 'answer':'Yes'] The summary content is as follows: content

- Open-ended Q&A evaluation (GPT4o): *You will be provided with a question, a ground truth (GT) answer, and a model-generated result related to a pathology case. Your task is to evaluate the correctness of the model's predicted result. You are the final judge and must follow these instructions: 1. If the model's result and the GT answer convey similar or equivalent information, it is correct, even if the wording or structure differs. 2. If the model's result is broader but includes the GT answer or its key elements, it should still be considered correct. 3. If part of the model's result matches part of the key information from the GT answer, the result is partially correct. 4. If the model's result and the GT answer do not overlap, but the result could plausibly serve as an alternate answer to the question, it is uncertain. 5. Since the GT answer is derived from a pathology report that summarizes information from multiple slides, it may not always be accurate or fully representative of the specific slide in question. Only GT answers directly related to a definitive diagnosis can be fully relied upon. 6. For morphology-related questions, the model's result should only be considered incorrect if it directly and completely contradicts the GT answer, such that both cannot plausibly coexist on the same slide. Provide your evaluation as a single number: 2 if the model's result is correct, 1 if it is partially correct, 0 if it is uncertain, -1 if it is incorrect. Ensure your assessment reflects the clinical context and the accuracy of the provided content.*
