# Supplementary material for: ALPaCA: Adapting Llama for Pathology Context Analysis to enable slide-level question answering
Source: Nat Commun. 2026 Aug 20;17:9943. doi: 10.1038/s41467-026-76372-z (PMC13586377; doi:10.1038/s41467-026-76372-z)
Supplement: Supplementary file 2 [file 41467_2026_76372_MOESM2_ESM.pdf]

nature portfolio

Corresponding author(s): Mireia Crispin-Ortuzar

Last updated by author(s): Jul 23, 2026

Reporting Summary

Nature Portfolio wishes to improve the reproducibility of the work that we publish. This form provides structure for consistency and transparency in reporting. For further information on Nature Portfolio policies, see our Editorial Policies and the Editorial Policy Checklist.

Statistics

For all statistical analyses, confirm that the following items are present in the figure legend, table legend, main text, or Methods section.

n/a	Confirmed	
☐	☒	The exact sample size (n) for each experimental group/condition, given as a discrete number and unit of measurement
☐	☒	A statement on whether measurements were taken from distinct samples or whether the same sample was measured repeatedly
☒	☐	The statistical test(s) used AND whether they are one- or two-sided<br>*Only common tests should be described solely by name; describe more complex techniques in the Methods section.*
☒	☐	A description of all covariates tested
☒	☐	A description of any assumptions or corrections, such as tests of normality and adjustment for multiple comparisons
☒	☐	A full description of the statistical parameters including central tendency (e.g. means) or other basic estimates (e.g. regression coefficient) AND variation (e.g. standard deviation) or associated estimates of uncertainty (e.g. confidence intervals)
☒	☐	For null hypothesis testing, the test statistic (e.g. F, t, r) with confidence intervals, effect sizes, degrees of freedom and P value noted<br>*Give P values as exact values whenever suitable.*
☒	☐	For Bayesian analysis, information on the choice of priors and Markov chain Monte Carlo settings
☒	☐	For hierarchical and complex designs, identification of the appropriate level for tests and full reporting of outcomes
☒	☐	Estimates of effect sizes (e.g. Cohen's d, Pearson's r), indicating how they were calculated

Our web collection on statistics for biologists contains articles on many of the points above.

Software and code

Policy information about availability of computer code

Data collection	Using GDC Application Programming Interface to download the whole slide image data from TCGA and GTEx. https://gdc.cancer.gov/developers/gdc-application-programming-interface-api, https://gtexportal.org/api/v2/redoc.<br>WSIs from XJTU-RG were scanned using a KFBIO-005 digital slide scanner (Jiangfeng Biology), and WSIs from CUH-OV were scanned using a Leica Aperio AT2 scanner (Leica Biosystems), both at 20× magnification (approximately 0.5 µm per pixel).
Data analysis	Using the custom code provided by us to do the data analysis. Pre-processing: https://github.com/ZeyuGaoAi/SMMILe, Model training and evaluation: https://github.com/ZeyuGaoAi/ALPaCA.<br>TCGA PDF pathology reports were converted to text using PyMuPDF. GPT-3.5 Turbo was used to summarise TCGA reports by removing gross-examination information while retaining microscopic pathological findings; GPT-4o then refined these summaries into slide-level captions with patient metadata. GTEx pathology notes were rephrased into slide-level captions using GPT-4o with tissue metadata. GPT-4o was used to generate slide-level question-answer pairs from the curated captions/notes.

For manuscripts utilizing custom algorithms or software that are central to the research but not yet described in published literature, software must be made available to editors and reviewers. We strongly encourage code deposition in a community repository (e.g. GitHub). See the Nature Portfolio guidelines for submitting code & software for further information.

Data

Policy information about availability of data

All manuscripts must include a data availability statement. This statement should provide the following information, where applicable:

- Accession codes, unique identifiers, or web links for publicly available datasets
- A description of any restrictions on data availability
- For clinical datasets or third party data, please ensure that the statement adheres to our policy

The TCGA WSIs, pathology reports, and associated clinical metadata are accessible via the NIH Genomic Data Commons (https://portal.gdc.cancer.gov).
The GTEx WSIs, pathology notes, and associated clinical metadata are available from the Genotype-Tissue Expression project (https://www.gtexportal.org/).
The curated datasets are publicly available on Hugging Face, including General Slide-Caption (CNX-PathLLM/TCGA-WSI-Description-4onew and CNX-PathLLM/GTEx-WSI-Description); General Slide-QA (CNX-PathLLM/TCGA-WSI-CloseQA-Balanced, CNX-PathLLM/GTEx-WSI-CloseQA-Balanced, CNX-PathLLM/TCGA-WSI-OpenQA and CNX-PathLLM/GTEx-WSI-OpenQA); Specific Slide-QA (Breast) (CNX-PathLLM/TCGA-BRCA-Details-CloseQA and CNX-PathLLM/TCGA-BRCA-Details-OpenQA); and Specific Slide-QA (Morphology) (CNX-PathLLM/PathChat-CloseQA-Balanced and CNX-PathLLM/PathChat-OpenQA). The datasets are available at https://huggingface.co/CNX-PathLLM/datasets.
The two external validation cohorts (XJTU-RG and CUH-OV) contain patient-derived clinical pathology data and cannot be made publicly available because of patient privacy, ethical approval and data protection restrictions.
Access requires approval from the corresponding institutional data controllers and ethics/governance committees and completion of an institutionally approved data-use agreement. XJTU-RG access requests should be directed to Chunbao Wang (bingliziliao2012@163.com) and reviewed by the First Affiliated Hospital of Xi'an Jiaotong University. CUH-OV access requests should be directed to Mireia Crispin-Ortuzar (mc973@cam.ac.uk) and reviewed by Cambridge University Hospitals NHS Foundation Trust and the CTCR-OV04 study governance body. Data can only be shared for non-commercial research purposes where permitted by the applicable approvals and data-transfer agreements.
Source data are provided with this paper.

Research involving human participants, their data, or biological material

Policy information about studies with human participants or human data. See also policy information about sex, gender (identity/presentation), and sexual orientation and race, ethnicity and racism.

Reporting on sex and gender	Sex and gender were not variables of interest in the study design or analysis and were not used as model inputs, labels or covariates. Although sex metadata are available for some TCGA and GTEx cases, they were not analysed in this study. No sex or gender data were collected for XJTU-RG, and the study team made no independent determination by self-report or assignment. The parent CTCR-OV04 study reported all CUH-OV participants as female, but did not report gender identity or the method of sex ascertainment. No sex- or gender-stratified analyses were performed because the study focused on developing and evaluating slide-level question answering rather than participant-level sex- or gender-associated differences.
Reporting on race, ethnicity, or other socially relevant groupings	Race, ethnicity and other socially relevant groupings were not variables of interest in the study design or analysis and were not used as model inputs, labels or covariates. The study team did not extract, infer or analyse these characteristics from whole-slide images, pathology reports or associated metadata. No analyses stratified by race, ethnicity or other socially relevant groupings were performed because the study focused on developing and evaluating slide-level question answering rather than participant-level demographic or social-group differences.
Population characteristics	Our study focused solely on the analysis of whole slide images and corresponding pathology reports. Several TCGA cohorts contain clinical data that has been previously reported, but we did not analyse it.
Recruitment	The XJTU-RG cohort was retrospectively collected from the First Affiliated Hospital of Xi'an Jiaotong University and includes patients diagnosed with renal cell carcinoma (RCC) or gastric cancer who underwent surgical resection between 2012 and 2019. From the eligible pool, 10 RCC cases and 10 gastric cancer cases were randomly selected. For each patient, one or two representative diagnostic whole-slide images were included. The CUH-OV cohort was obtained from Cambridge University Hospitals and consists of 10 ovarian cancer cases enrolled between 2021 and 2022 as part of the CRUK OV04 project (https://www.cancerresearchuk.org/about-cancer/find-a-clinical-trial/a-study-look-more-closely-factors-affecting-how-well-chemotherapy-works-for-cancer-ovary-ov04). Cases were randomly selected from the study cohort, with one to three diagnostic slides per patient included. For each case, diagnostic slides were selected by experienced pathologists as representative whole-slide images considered sufficient for routine histopathological diagnosis.
Ethics oversight	This research complied with all relevant ethical regulations. Publicly available de-identified TCGA and GTEx data were used in accordance with the terms and conditions of the respective data providers. The collection and use of data from the XJTU-RG cohort were approved by the Ethics Committee of the First Affiliated Hospital of Xi'an Jiaotong University (KYLLSL2021-420 and KYLLSL2022-333), which also approved a waiver of informed consent. The CUH-OV cohort was derived from the prospective Cambridge Translational Cancer Research Ovarian Study 04 (CTCR-OV04), approved by the Cambridgeshire and Hertfordshire Research Ethics Committee (REC reference: 08/H0306/61). All CUH-OV participants provided written informed consent for research use of their donated samples.

Note that full information on the approval of the study protocol must also be provided in the manuscript.

Field-specific reporting

Please select the one below that is the best fit for your research. If you are not sure, read the appropriate sections before making your selection.

☒ Life sciences ☐ Behavioural & social sciences ☐ Ecological, evolutionary & environmental sciences

For a reference copy of the document with all sections, see nature.com/documents/nr-reporting-summary-flat.pdf

Life sciences study design

All studies must disclose on these points even when the disclosure is negative.

Sample size	This study utilized all available cases from the TCGA and GTEx databases that contained both whole-slide images (WSIs, FFPE) and corresponding pathology reports or notes. No statistical methods were used to predetermine the sample size. Instead, we included the full set of qualified data to ensure broad coverage across various cancer types and tissue sites.<br>After excluding samples with missing WSIs or reports, the final dataset consisted of 11,634 TCGA cases and 24,279 GTEx cases, all of which were formalin-fixed paraffin-embedded (FFPE) WSIs accompanied by diagnostic pathology notes.<br>For external validation, two independent clinical cohorts were included. The XJTU-RG cohort consisted of 20 retrospectively collected cancer cases (renal cell carcinoma and gastric cancer), represented by 21 FFPE diagnostic slides. The CUH-OV cohort included 10 ovarian cancer cases from the OV04 study, comprising 21 FFPE diagnostic slides. These external cohorts were used exclusively for evaluation and were not involved in any stage of model training.
Data exclusions	Samples with corrupted or incomplete whole-slide image (WSI) files—such as those lacking stratification metadata—were excluded. Additionally, we excluded samples without corresponding pathology reports.
Replication	Based on the code and datasets we provided and referenced, all the results are reproducible.
Randomization	For internal testing and evaluation, we randomly selected three cases per non-tumour tissue site from the GTEx dataset and one to two cases per diagnosis type from the TCGA dataset as hold-out test cases, resulting in a total of 337 test cases. The remaining samples were used for model training. Random selection was performed to ensure diverse representation of tissue types and diagnostic categories in the test set. All test cases were strictly held out and were not used at any stage of model training, validation, or hyperparameter tuning.<br>In addition to this internal evaluation, external validation was performed on two independent clinical cohorts (XJTU-RG and CUH-OV), which were collected from separate medical institutions and were entirely disjoint from the TCGA and GTEx datasets. These external cohorts were not subject to random sampling and were instead evaluated in a fully held-out, zero-shot manner to assess real-world generalizability.
Blinding	During evaluation, especially for the open-ended question answering task, the annotators (expert pathologists) were blinded to GPT-4-based judgments to ensure unbiased assessment. For automatic evaluation, no blinding was applicable, as metrics were computed directly from model outputs and ground-truth annotations without human intervention.

Reporting for specific materials, systems and methods

We require information from authors about some types of materials, experimental systems and methods used in many studies. Here, indicate whether each material, system or method listed is relevant to your study. If you are not sure if a list item applies to your research, read the appropriate section before selecting a response.

Materials & experimental systems

n/a	Involved in the study
☒	☐ Antibodies
☒	☐ Eukaryotic cell lines
☒	☐ Palaeontology and archaeology
☒	☐ Animals and other organisms
☒	☐ Clinical data
☒	☐ Dual use research of concern
☒	☐ Plants

Methods

n/a	Involved in the study
☒	☐ ChIP-seq
☒	☐ Flow cytometry
☒	☐ MRI-based neuroimaging

Plants

Seed stocks	*Report on the source of all seed stocks or other plant material used. If applicable, state the seed stock centre and catalogue number. If plant specimens were collected from the field, describe the collection location, date and sampling procedures.*
Novel plant genotypes	*Describe the methods by which all novel plant genotypes were produced. This includes those generated by transgenic approaches, gene editing, chemical/radiation-based mutagenesis and hybridization. For transgenic lines, describe the transformation method, the number of independent lines analyzed and the generation upon which experiments were performed. For gene-edited lines, describe the editor used, the endogenous sequence targeted for editing, the targeting guide RNA sequence (if applicable) and how the editor was applied.*
Authentication	*Describe any authentication procedures for each seed stock used or novel genotype generated. Describe any experiments used to assess the effect of a mutation and, where applicable, how potential secondary effects (e.g. second site T-DNA insertions, mosiacism, off-target gene editing) were examined.*
