# Supplementary material for: ALPaCA: Adapting Llama for Pathology Context Analysis to enable slide-level question answering
Source: Nat Commun. 2026 Aug 20;17:9943. doi: 10.1038/s41467-026-76372-z (PMC13586377; doi:10.1038/s41467-026-76372-z)
Supplement: Supplementary file 3 [file 41467_2026_76372_MOESM3_ESM.pdf]

nature portfolio

Peer Review File

ALPaCA: Adapting Llama for Pathology Context Analysis to enable slide-level question answering

Corresponding Author: Dr Mireia Crispin-Ortuzar

This file contains all reviewer reports in order by version, followed by all author rebuttals in order by version.

Version 0:

Reviewer comments:

Reviewer #1

(Remarks to the Author)
The authors present ALPACA, a framework designed to enable WSI question answering in computational pathology. The main novelty is the development of a slide-level Large Vision Language Model that can analyze gigapixel-scale pathology images and answer both general and specialized medical questions. The authors propose various slide-level adaptors to bridge a pathology vision encoder (CONCH) with Llama3.1, with their hybrid approach (combining LongFormer and GMM adaptors) achieving the best performance.

Major concerns:
1. The authors used CONCH as patch level encoder and claimed that CONCH is better suited for LLM integration than other pre-trained vision-only pathology image encoders without providing any context or explanation. However, several other SOTA Vision-Language foundational models in digital pathology have been developed. There needs to be a detailed comparison of different models after slide level integration.

2. The typical pathologist work flow starts from low power observation to high power assessment in almost all tasks. Low magnification examination is a critical first step in the pathologist's workflow when diagnosing cancer as well as other pathology assessments. The routine low power slide level tasks are one of the biggest the reasons why a slide level Q-A model is needed in digital pathology. The most typical use cases include identification potentially precancerous lesions on epithelial surfaces; qualitative and relative quantitative assessment of large structures or structures that appear all over the tissue. For example, assessing the proportion of sclerotic glomeruli amongst all glomeruli in a patient. Or assess the percentage of tissue area that a fibrotic. Pathology training also includes developing the visual mechanisms that support efficient examination of slides at low magnification. In practice, initial low-power viewing helps pathologists sort cases into "straightforward" versus "requiring further investigation" categories. Benign cases with clear visual cues can often be diagnosed at low magnification without requiring immunohistochemistry.

However, in this study, the questions are mixed between low power optimized tasks and high-power optimized tasks. For example, questions like “What characteristics do the tumor cells display in this slide?” is a cellular level pathology assessment question and needs to be answered by zooming in to the high-power resolution. While a question like primary diagnosis is an obvious low power question. In that light, the slide level QA, especially the questions, need to be divided into high power questions and low power questions by pathologists. The question categories are great in terms of understanding the model’s performance in a specific task, but I think dividing the tasks to low-power oriented vs high-power oriented questions in pathologist manual assessment can help better understand the model performance.

3. Following my previous comment - another critical reason why a slide level QA model is needed, is to mimic the pathologists’ visual assessment and decision making process – by deciding which areas are critical to diagnosis or prognosis, and to zoom in to these regions and observe the tissue morphology closely and then perform any downstream task needed. While in this study, the authors used various patch aggregation methods for slide level implementation, and ffter a detailed comparison of six candidate adaptors, we proposed the hybrid adaptor (ALPaCA-Hybrid), which achieved the best performance across multiple evaluation scenarios. However, the slide-level descriptions and QA pairs from patient-level pathology reports are not completely reliable, and ALPaCA lacks explicit spatial grounding. This brings up the question: Why not focus on implementation of at least some basic level of spatial interpretability? Out of all the slide adaptors the author proposed, the ABMIL and Qformer can both be used to generate attention heatmaps across all patches

on the WSIs. This is critical to slide level QA, to assess whether the model is looking at the correct regions when answering the high-power questions. I would argue that spatial interpretability of the model is much more important than a few percent's trade-off in accuracy.

4. Following comments 2 and 3. The study stops at a proof of concept but lacks demonstration of clinical utility via downstream use cases leveraging more diverse or private datasets. It would be essential that the authors show the model's clinical utility and accuracy on both high power and low power suited clinical tasks.

5. The use of GPT-3.5 and GPT-4 for summarizing and generating pathology reports and QA pairs introduces potential bias and noise, as acknowledged by the authors. However, there is no quantification of how much this synthetic data diverges from pathologist-authored ground truth, nor an ablation study to assess the impact of this synthetic data on model performance. This is particularly relevant given the known limitations of LLM-generated medical text.

Minor concerns:
1. There is no discussion of how the model handles questions that are genuinely unanswerable from a single WSI (e.g., due to missing tissue or ambiguous findings), nor is there a mechanism for the model to abstain or express uncertainty.
2. The manuscript does not report inference times, memory requirements, or scalability to larger datasets or real-time clinical workflows. This information is important for assessing practical deployment.

(Remarks on code availability)

Reviewer #2

(Remarks to the Author)
1. Overall assessment
The paper presents the first general-purpose large vision–language model for whole-slide pathology question answering (WSI-QA), named ALPaCA. The authors build a complete pipeline covering dataset construction, adapter design, and evaluation. They automatically generate 36 k slide-level captions and 340 k QA pairs from public TCGA/GTEx slides, explore six types of visual / vision-language adapters, and finally propose a Hybrid LongFormer + GMM solution. The model achieves > 90 % accuracy on closed-form QA; on open-form QA it outperforms competitors under both GPT-4o-based and human pathologist evaluations, and it can be further fine-tuned for breast cancer molecular subtyping and multi-cancer morphology tasks. The work is innovative and potentially useful, yet there are issues regarding data quality, evaluation sufficiency, and methodological details.

2. Strengths
• Clear problem definition: addresses a genuine clinical need—multi-region, multi-magnification reasoning on the same WSI—an ability missing in existing ROI-level LVLMs.
• Dataset contribution: automatically produces the largest slide-level caption & QA dataset from public WSIs and reports/notes, with an intention to release it to the community.
• Systematic exploration: compares six adapter families (Sampling / KMeans / GMM / ABMIL / Q-Former / LongFormer) and proposes a Hybrid scheme; the experimental design is fairly thorough.
• Multi-layer evaluation: combines closed-form, open-form, automatic metrics, GPT-4o scoring, and expert pathologist review to mitigate the intrinsic subjectivity of open QA.
• Transferability: fine-tuning on two disease-specific QA sets shows good performance, demonstrating extensibility.

3. Major concerns
1) Data quality & label consistency
• A large fraction of QAs are generated by GPT-4o from pathology reports, yet one report may correspond to several slides. The authors acknowledge the presence of "unanswerable" or noisy labels. Although later filtered, experts still flagged a considerable portion of the held-out test questions, indicating residual noise.
• Please describe the human QC in detail (sampling rate, correction rules, final noise ratio) and report the main results on the "expert-answerable subset" to reflect the true model capability.

2) Evaluation sufficiency & missing baselines
• Comparisons are limited to the authors'six adapters; no direct comparison with the most relevant prior work (Prism, PathAlign, PathChat, multimodal WSI foundation models, etc.). Hence the magnitude of improvement is unclear.
• No "vision-free" or "random patch" baseline is provided, nor the performance of a pure Llama-3.1 using slide report / caption only. Such baselines are needed to quantify the real contribution of visual information.

3) LongFormer design & computational cost
• Only a brief mention of a "two-layer LongNet-based structure" is given. Key hyper-parameters (number of tokens, window size, sparsity scheme, complexity analysis) and ablations are missing.
• How are memory footprint and latency handled with an 8 B Llama plus > 1 M patch tokens? Have you run batch > 1 sampling on two 80 GB GPUs? Please add resource usage statistics and inference speed.

4) Generalization & external validation
• The test set contains only 337 slides, all still from TCGA/GTEx, likely close to the training distribution. There is no evaluation on independent institutions, scanners, or rare cancers, so generalization remains unproven.

• Please add at least one external open dataset (e.g., PAIP, CAMELYON, PANDA) or an in-house clinical cohort for zero/low-shot evaluation.

4. Suggestions for improvement
1) Comparative experiments: add results vs. public LVLMs (Prism, PathAlign, PathChat, Pathologyvlm) and vs. text-only / random-vision baselines. If code is unavailable, re-implement or cite the original reported numbers.
2) Data QC: describe the expert filtering process in the appendix and provide main results on the expert-approved subset. Consider releasing a public test leaderboard for future methods.
3) External validation: perform zero-shot tests on a small real-world clinical WSI set (anonymized) or community challenge data to show robustness to domain shift.
4) Explainability: include visualizations—heatmaps or patch-level attentions—for correct and incorrect cases to enhance interpretability.

5. Conclusion & recommendation
The manuscript presents a systematic framework and reproducible data/code resources for slide-level multimodal QA, which could push pathology LVLM research forward. However, data noise, missing baselines, and lack of external validation currently weaken the empirical evidence. Further experiments and methodological clarifications are required before publication.

(Remarks on code availability)

Reviewer #3

(Remarks to the Author)
I co-reviewed this manuscript with one of the reviewers who provided the listed reports. This is part of the Nature Communications initiative to facilitate training in peer review and to provide appropriate recognition for Early Career Researchers who co-review manuscripts.

(Remarks on code availability)

Version 1:

Reviewer comments:

Reviewer #1

(Remarks to the Author)
I thank the authors for their detailed rebuttal and effort put into the revised manuscript. However, several critical issues remain unresolved. My remaining major concerns are detailed below:

1. "General-Purpose" claims contradicted by external validation. The abstract and introduction continue to frame ALPACA as the "first general-purpose slide-level LVLM". However, the new external validation results contradict this broad claim. Specifically, performance on the external CUH-OV (ovarian) cohort dropped significantly to ~77% (compared to >90% on internal sets), which the authors attribute to "dataset-level mismatches" and a lack of exposure to "fine-grained subtype diversity". This indicates the model is brittle to distribution shifts and is not robustly "general-purpose." Authors should provide more convincing evidence for these broad claims, or they should be tempered in the Abstract and Introduction. The term "general-purpose" should be qualified to reflect the specific training distribution (TCGA/GTEx contexts). Furthermore, the Abstract should explicitly mention the performance degradation observed in external cohorts to provide a scientifically balanced view of the model's current capabilities.

2. Lack of downstream clinical task demonstration. The manuscript claims ALPACA is general purpose and enhances "diagnostic decision-making". However, the evaluation relies almost exclusively on aggregated QA correctness (e.g., multiple-choice accuracy). "Answering a question correctly" is often a proxy for clinical utility rather than a demonstration of it. To justify the "general-purpose" claim, the authors must demonstrate competence in standard pathological tasks using standard clinical metrics. Without clinical task validation the “general purpose” claim does not stand. Recommend authors to perform a retrospective analysis on the existing test sets (or external cohorts) to report standard metrics for at least one core pathological task beyond QA accuracy. For example, for the "Tumor Grade and Staging" category, provide a Confusion Matrix and Kappa score comparing ALPACA's predicted grade vs. the ground truth. Or, for the "Tumor Detection" category, report Sensitivity and Specificity rather than just accuracy.

3. There is an unexplained discrepancy in how the external cohorts were evaluated. The XJTU-RG cohort was evaluated on both close-ended and open-ended tasks , but the CUH-OV cohort appears to have been evaluated only on close-ended questions (79 pairs). Please provide open-ended evaluation results for the CUH-OV cohort or explicitly explain why this was omitted. Given the lower performance on close-ended questions for this cohort, omitting the open-ended results (the model's primary novelty) obscures potential failure modes.

4. Specific Slide-QA (Morphology) is constructed using PathChat-generated patch descriptions that are summarized by

GPT-4o into slide-level reports, and these AI-generated reports are then used to curate QA pairs. This introduces a risk that the benchmark measures consistency with PathChat/GPT-4o-generated priors rather than independent, pathologist-verified ground truth. This is a significant limitation that should be addressed.

(Remarks on code availability)

Reviewer #2

(Remarks to the Author)
authors have addresses all my concerns.

(Remarks on code availability)

Reviewer #3

(Remarks to the Author)
I co-reviewed this manuscript with one of the reviewers who provided the listed reports. This is part of the Nature Communications initiative to facilitate training in peer review and to provide appropriate recognition for Early Career Researchers who co-review manuscripts.

(Remarks on code availability)

Version 2:

Reviewer comments:

Reviewer #1

(Remarks to the Author)
All prior critiques have been addressed in this revision. I have no other concerns.

(Remarks on code availability)

Open Access This Peer Review File is licensed under a Creative Commons Attribution 4.0 International License, which permits use, sharing, adaptation, distribution and reproduction in any medium or format, as long as you give appropriate credit to the original author(s) and the source, provide a link to the Creative Commons license, and indicate if changes were made.
In cases where reviewers are anonymous, credit should be given to 'Anonymous Referee' and the source.

The images or other third party material in this Peer Review File are included in the article's Creative Commons license, unless indicated otherwise in a credit line to the material. If material is not included in the article's Creative Commons license and your intended use is not permitted by statutory regulation or exceeds the permitted use, you will need to obtain permission directly from the copyright holder.
To view a copy of this license, visit https://creativecommons.org/licenses/by/4.0/

Point-by-point responses

Reviewer #1 (Remarks to the Author):

The authors present ALPACA, a framework designed to enable WSI question answering in computational pathology. The main novelty is the development of a slide-level Large Vision Language Model that can analyze gigapixel-scale pathology images and answer both general and specialized medical questions. The authors propose various slide-level adaptors to bridge a pathology vision encoder (CONCH) with Llama3.1, with their hybrid approach (combining LongFormer and GMM adaptors) achieving the best performance.

Response: We thank the reviewer for appreciating the advantages of our work and are glad to receive these detailed and constructive comments. Below we address the comments one by one.

Major concerns

Comment 1: The authors used CONCH as patch level encoder and claimed that CONCH is better suited for LLM integration than other pre-trained vision-only pathology image encoders without providing any context or explanation. However, several other SOTA Vision-Language foundational models in digital pathology have been developed. There needs to be a detailed comparison of different models after slide level integration.

Response: Thanks for this insightful comment. In the revised manuscript, we have substantially expanded our analysis of patch-level encoders and their impact on downstream slide-level QA performance. Specifically, we now include new ablation experiments in which ALPaCA is equipped with three additional pathology foundation models, MUSK (Nature, 2025), UNI (Nature Medicine, 2024), and PathGen (ICLR, 2025), in place of the default CONCH encoder. MUSK and PathGen are vision–language pathology foundation models, similar to CONCH, whereas UNI is a vision-only pathology foundation model. Note that previous studies report that these pathology foundation models achieve comparable performance on patch-level classification and retrieval tasks, with PathGen generally exhibiting slightly lower representational strength.

Our experimental results show that *the capacity of pathology foundation models is more critical than modality alignment for slide-level QA*: given a reasonably strong

pathology foundation model, ALPaCA-Hybrid is relatively insensitive to whether the encoder is vision–language or vision-only (with CONCH and MUSK performing slightly better than UNI), while performance degrades more noticeably when the encoder capacity is reduced.

Action:

- ➔ We have revised our original wording regarding CONCH in the Method section (Pre-processing of WSIs): instead of stating that "CONCH is better suited for LLM integration than other vision-only encoders," we now describe CONCH as "well suited" for integration with LLMs (**Page 20**).
- ➔ We have added the new ablation results and their description to a dedicated section entitled "Patch encoders"(Supplementary Note, **Page 1**), and we present the corresponding comparisons in **Extended Data Fig. 5a,b** (Supplementary Note, **Page 28**).

Comment 2: The typical pathologist work flow starts from low power observation to high power assessment in almost all tasks. Low magnification examination is a critical first step in the pathologist's workflow when diagnosing cancer as well as other pathology assessments. The routine low power slide level tasks are one of the biggest the reasons why a slide level Q-A model is needed in digital pathology. The most typical use cases include identification potentially precancerous lesions on epithelial surfaces; qualitative and relative quantitative assessment of large structures or structures that appear all over the tissue. For example, assessing the proportion of sclerotic glomeruli amongst all glomeruli in a patient. Or assess the percentage of tissue area that a fibrotic. Pathology training also includes developing the visual mechanisms that support efficient examination of slides at low magnification. In practice, initial low-power viewing helps pathologists sort cases into "straightforward" versus "requiring further investigation" categories. Benign cases with clear visual cues can often be diagnosed at low magnification without requiring immunohistochemistry.

However, in this study, the questions are mixed between low power optimized tasks and high-power optimized tasks. For example, questions like "What characteristics do the tumor cells display in this slide?" is a cellular level pathology assessment question and needs to be answered by zooming in to the high-power resolution. While a question like primary diagnosis is an obvious low power question. In that light, the slide level QA, especially the questions, need to be divided into high power questions and low power questions by pathologists. The question categories are great in terms of understanding the model's performance in a specific task, but

I think dividing the tasks to low-power oriented vs high-power oriented questions in pathologist manual assessment can help better understand the model performance.

Response: We completely agree and thank you for suggesting a stratified analysis on this aspect. In the revised manuscript, we now analyse ALPaCA's behaviour on questions that predominantly rely on low-power global assessment versus those that require high-power, cellular-level inspection. A pathologist (C.W.) reviewed all close-ended and open-ended questions in the "General Slide-QA" test set and annotated based on diagnostic intent, whether each question would typically be answered at low magnification or would require high-magnification examination.

This analysis shows that TCGA contains a larger proportion of high-power questions (e.g. grading, invasion, cell-level analysis), whereas GTEx questions are predominantly low-power and focus on global architectural features. For close-ended QA, ALPaCA-Hybrid achieved highly similar accuracies on low- and high-power questions in both TCGA and GTEx (differences <1%). In contrast, for open-ended QA we observe a clearer magnification effect: both GPT-4o and expert evaluation indicate slightly higher "totally correct" rates for low-power questions, with high-power questions more often yielding partially correct descriptions, reflecting the greater challenge of capturing fine-grained cytological and subcellular details at slide level without the support of predefined answer options. Overall, these results provide a more nuanced view of ALPaCA's performance across low- and high-power use cases.

Action:

➔ We have added a new section entitled **"**Low- vs high-power question analysis**"** (Supplementary Note, **Page 6**). The question distributions and results are presented in **Extended Data Fig. 7** (Supplementary Note, **Page 30**).

Comment 3: Following my previous comment - another critical reason why a slide level QA model is needed, is to mimic the pathologists' visual assessment and decision making process – by deciding which areas are critical to diagnosis or prognosis, and to zoom in to these regions and observe the tissue morphology closely and then perform any downstream task needed. While in this study, the authors used various patch aggregation methods for slide level implementation, and ffter a detailed comparison of six candidate adaptors, we proposed the hybrid adaptor (ALPaCA-Hybrid), which achieved the best performance across multiple evaluation scenarios. However, the slide-level descriptions and QA pairs from

patient-level pathology reports are not completely reliable, and ALPaCA lacks explicit spatial grounding. This brings up the question: Why not focus on implementation of at least some basic level of spatial interpretability? Out of all the slide adaptors the author proposed, the ABMIL and Qformer can both be used to generate attention heatmaps across all patches on the WSIs. This is critical to slide level QA, to assess whether the model is looking at the correct regions when answering the high-power questions. I would argue that spatial interpretability of the model is much more important than a few percent's trade-off in accuracy.

Response: We agree with the reviewer that spatial interpretability is an important aspect of slide-level QA. In this study, our primary objective was to establish the feasibility and performance characteristics of slide-level QA at scale, using patient-level pathology reports as supervision and systematically comparing different slide adaptors. As discussed in the Discussion, explicit spatial grounding was not imposed as a design constraint in this initial framework.

Although ALPaCA-Hybrid is not constructed as an explicitly spatially grounded model, its two core components, the GMM-based prototype maps and the LongFormer-based attention mechanism, enable a form of qualitative spatial analysis that provides insight into the model's internal reasoning. The GMM adaptor produces a global, question-independent partitioning of each slide into visually coherent regions across magnification levels, offering an overview of the morphological structures recognised by the model. In contrast, the LongFormer adaptor supports question-conditioned attention visualisation, highlighting how visual evidence is differentially weighted under distinct diagnostic intents.

We illustrate these behaviours using four representative cases. The examples show how attention patterns vary with both question type and magnification level, including high-power questions that require fine-grained morphological cues. We emphasise that, due to dense visual tokenisation and the absence of explicit sparsity or localisation constraints, these attention maps are intended to be interpreted comparatively across questions rather than as strict spatial localisation signals.

Finally, we fully agree that incorporating stronger spatial grounding mechanisms, such as explicit region selection, hierarchical zooming strategies, or integration with region-level annotations. This represents an important and natural direction for future work. We view the current study as a foundational step toward slide-level QA, upon which more explicitly spatially grounded and pathologist-interactive models can be developed.

Action:

- ➔ We have added a new “Visualisation” section (Supplementary Note, **Page 3**) and included qualitative visualisations for four representative cases in **Extended Data Fig. 6** (Supplementary Note, **Page 29**).

Comment 4: Following comments 2 and 3. The study stops at a proof of concept but lacks demonstration of clinical utility via downstream use cases leveraging more diverse or private datasets. It would be essential that the authors show the model’s clinical utility and accuracy on both high power and low power suited clinical tasks.

Response: Agreed, this is an important point. A key limitation previously was the absence of any publicly recognised or standardised slide–QA benchmark dataset for evaluating clinically relevant slide-questioning tasks. To address the reviewer’s comment, we have now constructed two private, pathologist-curated external datasets specifically designed to assess the general QA capabilities of our model in real clinical utility.

The first dataset includes two cancer types (renal cell and gastric) and six diagnostic categories, comprising 20 cases and a total of 297 QA pairs. The second dataset focuses on ovarian cancer but spans six tissue sites and five diagnostic categories, containing 10 cases and 79 QA pairs. Importantly, all questions in both datasets were explicitly separated into low-power and high-power categories.

Action:

- ➔ We have added detailed descriptions of the two external cohorts in Methods: Curation of datasets (Evaluation datasets, **Page 17**). Their evaluation results are reported in Results: Evaluation on independent clinical cohorts (**Page 10**), Fig. 4d,e (**Page 41**), and **Extended Data Fig. 7** (Supplementary Note, **Page 30**).

Comment 5: The use of GPT-3.5 and GPT-4 for summarizing and generating pathology reports and QA pairs introduces potential bias and noise, as acknowledged by the authors. However, there is no quantification of how much this synthetic data diverges from pathologist-authored ground truth, nor an ablation study to assess the impact of this synthetic data on model performance. This is particularly relevant given the known limitations of LLM-generated medical text.

Response: We thank the reviewer for raising this important point. To address this concern, our expert-pathologists evaluated the quality of the LLM-generated data. Specifically, they reviewed a randomly sampled set of 40 cases (20 from TCGA and

20 from GTEx) together with their corresponding captions and 430 associated QA pairs.

The assessment results show that the LLM-generated captions were largely reliable, with only 10–20% of cases exhibiting minor omissions involving small ancillary details, and hallucinations occurring in approximately 5% of cases, and almost all of which were plausible inferences. For the QA data, around 98% of answers were correct and roughly 88% were answerable based on the information visible on the diagnostic slides. We also observed that synthetic noise was slightly more pronounced in TCGA than in GTEx, primarily because TCGA uses PDF-formatted patient reports whereas GTEx provides more structured text-based slide notes.

These findings demonstrate that the LLM-generated supervision is of high fidelity with limited synthetic noise. Given that the omissions and hallucinations were minimal and clinically non-misleading, and that the majority of QA items were both correct and answerable, we expect negligible adverse impact on model performance.

Action:

➔ We have added a new subsection entitled "Quality control" in Methods: Curation of datasets (**Page 18**) and presents the corresponding statistical results in **Extended Data Fig. 1g,h** (Supplementary Note, **Page 24**).

Minor concerns

Comment 6: There is no discussion of how the model handles questions that are genuinely unanswerable from a single WSI (e.g., due to missing tissue or ambiguous findings), nor is there a mechanism for the model to abstain or express uncertainty.

Response: We thank the reviewer for raising this important issue. In the current work, our primary goal was to establish the feasibility and performance characteristics of slide-level QA under the simplifying assumption that questions are answerable from a single WSI. Accordingly, our experimental setup does not include an explicit mechanism for abstention or uncertainty estimation, and we did not systematically identify or model genuinely unanswerable cases. We fully agree that, in realistic diagnostic workflows, such cases are common and that a clinically useful system must be able to recognise when a question cannot be reliably answered from the available slide alone. In the revised manuscript, we now explicitly acknowledge this limitation and outline potential extensions. We regard these abstention and

uncertainty-aware mechanisms as essential next steps toward safe deployment, but beyond the scope of this study.

Action:

➔ We have added a dedicated discussion in the Discussion section (**Page 14**).

Comment 7: The manuscript does not report inference times, memory requirements, or scalability to larger datasets or real-time clinical workflows. This information is important for assessing practical deployment.

Response: In the current study, all inference experiments were conducted in full FP32 precision on a single NVIDIA A100 (80GB). Under this setting, ALPaCA-Hybrid requires approximately 37–38 GB GPU memory during inference and achieves an average latency of ~0.22 seconds per QA using pre-extracted and cached patch features. The reported timings therefore reflect only the multimodal reasoning stage and exclude offline WSI feature extraction, which is performed once per slide. We note that, in practical settings, WSI feature extraction typically dominates the overall computational cost, often ranging from tens of seconds to several minutes per slide depending on slide size and resolution. This, however, is a common characteristic of computational pathology workflows and is routinely addressed through offline or post-scanning precomputation of patch/slide features. Once extracted, these features can be efficiently reused across multiple downstream tasks, including slide-level QA, enabling the reasoning stage to be performed with low latency.

Action:

➔ We have added a detailed description of inference-time hardware requirements, memory usage, and runtime performance to Methods: Evaluation (**Page 25**) and Hardware and software (**Page 27**).

Reviewer #2 (Remarks to the Author):

The paper presents the first general-purpose large vision–language model for whole-slide pathology question answering (WSI-QA), named ALPaCA. The authors build a complete pipeline covering dataset construction, adapter design, and evaluation. They automatically generate 36 k slide-level captions and 340 k QA pairs from public TCGA/GTEx slides, explore six types of visual / vision-language adapters, and finally propose a Hybrid LongFormer + GMM solution. The model

achieves > 90 % accuracy on closed-form QA; on open-form QA it outperforms competitors under both GPT-4o-based and human pathologist evaluations, and it can be further fine-tuned for breast cancer molecular subtyping and multi-cancer morphology tasks. The work is innovative and potentially useful, yet there are issues regarding data quality, evaluation sufficiency, and methodological details.

Strengths:

• Clear problem definition: addresses a genuine clinical need—multi-region, multi-magnification reasoning on the same WSI—an ability missing in existing ROI-level LVLMs.

• Dataset contribution: automatically produces the largest slide-level caption & QA dataset from public WSIs and reports/notes, with an intention to release it to the community.

• Systematic exploration: compares six adapter families (Sampling / KMeans / GMM / ABMIL / Q-Former / LongFormer) and proposes a Hybrid scheme; the experimental design is fairly thorough.

• Multi-layer evaluation: combines closed-form, open-form, automatic metrics, GPT-4o scoring, and expert pathologist review to mitigate the intrinsic subjectivity of open QA.

• Transferability: fine-tuning on two disease-specific QA sets shows good performance, demonstrating extensibility.

Conclusion & recommendation

The manuscript presents a systematic framework and reproducible data/code resources for slide-level multimodal QA, which could push pathology LVLM research forward. However, data noise, missing baselines, and lack of external validation currently weaken the empirical evidence. Further experiments and methodological clarifications are required before publication.

Response: We would like to thank the reviewer for their thorough review, positive comments and extremely useful suggestions. Below we address the questions one by one.

Major concerns

Comment 1: Data quality & label consistency

A large fraction of QAs are generated by GPT-4o from pathology reports, yet one report may correspond to several slides. The authors acknowledge the presence of “unanswerable” or noisy labels. Although later filtered, experts still flagged a considerable portion of the held-out test questions, indicating residual noise.

Please describe the human QC in detail (sampling rate, correction rules, final noise ratio) and report the main results on the “expert-answerable subset” to reflect the true model capability.

Response: We thank the reviewer for this great suggestion. To assess the fidelity of LLM-generated supervision, we conducted a dedicated human QC study on a randomly sampled subset from "General Slide-QA" training data, comprising 40 cases (20 TCGA and 20 GTEx), together with their corresponding slide captions and 430 associated QA pairs. Each QA pair was independently reviewed by expert pathologists.

This QC analysis showed that the majority of QA pairs were both clinically correct and answerable at the slide level, with only a limited fraction exhibiting ambiguity due to report–slide misalignment. Importantly, the identified omissions or hallucinations were minimal and non–clinically misleading, indicating that LLM-generated supervision is of high fidelity with low residual noise.

Action:

➔ We revised the reporting of open-ended QA results in the main manuscript. All primary open-ended evaluation results are now reported exclusively on the expert-answerable subset, as shown in Results: Automatic and pathologist evaluation of open-ended questions (**Pages 7-10**), **Fig. 3b,c** (**Page 40**) and **Extended Data Fig. 3** (Supplementary Note, **Page 26**).

➔ We have added a new subsection entitled "Quality control" in Methods: Curation of datasets (**Page 18**) and presents the corresponding statistical results in **Extended Data Fig. 1g,h** (Supplementary Note, **Page 24**).

Comment 2: Evaluation sufficiency & missing baselines

• Comparisons are limited to the authors'six adapters; no direct comparison with the most relevant prior work (Prism, PathAlign, PathChat, multimodal WSI foundation models, etc.). Hence the magnitude of improvement is unclear.

• No "vision-free" or "random patch" baseline is provided, nor the performance of a pure Llama-3.1 using slide report / caption only. Such baselines are needed to quantify the real contribution of visual information.

Response: We agree that appropriate baselines are critical for contextualizing performance gains in slide-level QA. To address this concern, we substantially expanded the baseline comparisons in the revised manuscript.

First, we benchmarked ALPaCA against four representative large vision–language models (LVLMs) that are widely used for multimodal reasoning tasks: Gemini-2.0, GPT-4o, QwenVL-Plus, and MedGemma-4B, using standardized input strategies

(WSI thumbnails and representative patches) adapted for gigapixel WSIs. In addition, we included SlideChat, a pathology-oriented slide-level LVLM with domain-specific priors, as the most relevant domain-specific comparator. Regarding other prior pathology models such as Prism and PathAlign, these approaches are designed for report or caption generation rather than slide-level question answering. They do not support flexible open- or close-ended QA formulations and therefore are not directly comparable under our evaluation protocol.

Second, to explicitly quantify the contribution of visual information, we added two vision-ablation baselines: (1) a text-only baseline, where the model receives only the question or prompt without any visual input; and (2) a random-image baseline, where pseudo-visual tokens are sampled from the empirical distribution of WSI embeddings, controlling for token scale while removing meaningful histological content.

Action:

- ➔ We have added comparison results and corresponding analyses of representative LVLM baselines in the Results sections (**Pages 7 and 9**), as well as in **Fig. 4** (**Page 41**). Detailed descriptions of the benchmarked LVLMs and evaluation protocols are provided in Methods: Benchmarks (**Page 27**).
- ➔ We have added a new “Baseline controls” section and **Extended Data Fig. 5c** (Supplementary Note, **Pages 2 and 27**) to report the performance of the text-only and random-image baselines.

Comment 3: LongFormer design & computational cost

• Only a brief mention of a “two-layer LongNet-based structure” is given. Key hyper-parameters (number of tokens, window size, sparsity scheme, complexity analysis) and ablations are missing.

• How are memory footprint and latency handled with an 8 B Llama plus > 1 M patch tokens? Have you run batch > 1 sampling on two 80 GB GPUs? Please add resource usage statistics and inference speed.

Response: In ALPaCA-Hybrid, each magnification level employs a lightweight LongNet module consisting of two layers with 512 hidden dimensions and 8 attention heads. LongNet adopts a dilated segment-based sparse attention scheme (rather than sliding-window attention), with dilation ratios {1,2,4,8,16} and corresponding segment lengths {1024, 1448, 2048, 2896, 4096}. The effective number of visual tokens is determined by the number of extracted patches per slide, while positional encodings support up to 10^6 patch positions. This configuration follows the original

LongNet design but uses a reduced depth and hidden size to balance expressivity and efficiency. We did not perform exhaustive ablations over LongNet hyper-parameters, as such experiments would be prohibitively expensive for large-scale slide-level QA training.

Regarding the training and inference resource usage, patch features are pre-extracted and cached, and we additionally cap the maximum number of patch tokens per WSI at 15,000 to avoid out-of-memory failures on exceptionally large slides. Training was conducted on two NVIDIA A100 (80GB) GPUs with distributed training; under the 15k-token cap, the peak GPU memory reached 68.29 GB per GPU in Stages 2–3 (ALPaCA-Hybrid), where the 8B LLM was fully fine-tuned (batch size = 4, gradient accumulation = 8). For inference, all experiments were run in full FP32 precision using the cached patch features (i.e., timings exclude the one-off offline feature extraction stage). Under this setting, ALPaCA-Hybrid required approximately 37–38 GB GPU memory (tested with batch sizes 1–16) and achieved an average latency of ~0.22 seconds per question.

Action:

- ➔ We have added the details of the LongNet-based adaptor to the Methods: ALPaCA architecture and various adaptors section (**Page 23**).
- ➔ We have added the training and inference resource usage to the Methods: Three-stage training of ALPaCA and Evaluation sections, respectively (**Page 24-25**).

Comment 4: Generalization & external validation
• The test set contains only 337 slides, all still from TCGA/GTEx, likely close to the training distribution. There is no evaluation on independent institutions, scanners, or rare cancers, so generalization remains unproven.
• Please add at least one external open dataset (e.g., PAIP, CAMELYON, PANDA) or an in-house clinical cohort for zero/low-shot evaluation.

Response: We agree that evaluation beyond TCGA/GTEx is essential for assessing generalization. However, most publicly available WSI datasets (e.g., CAMELYON, PAIP, PANDA) provide slides without corresponding slide-level descriptive text or question–answer annotations, which makes them unsuitable for evaluating general pathology QA under our formulation.

To address this limitation, we have added two independent, institutionally distinct clinical cohorts for external validation. The first cohort (XJTU-RG) was collected from the First Affiliated Hospital of Xi’an Jiaotong University and includes renal cell

carcinoma and gastric cancer cases with expert-curated open- and close-ended QA pairs. The second cohort (CUH-OV) was obtained from Cambridge University Hospitals and consists of ovarian cancer cases spanning multiple subtypes and tissue sites.

For both cohorts, all QA pairs were authored and validated by experienced pathologists following the same schema as the "General Slide-QA" dataset, with questions and answers generated directly from the diagnostic slides. These evaluations were performed in a zero-shot setting and demonstrate that ALPaCA-Hybrid maintains strong performance under meaningful distribution shifts in institution, cancer type, and tissue context.

Action:

➔ We added external validation experiments to assess generalization, evaluating ALPaCA-Hybrid on two independent private clinical cohorts and comparing it against the available open-source LVLM baselines. The results are shown in Results: Evaluation on Independent Clinical Cohorts (Page 10-12) section and **Fig. 4d,e** (Page 41)

Comment 5: Suggestions for improvement

1) Comparative experiments: add results vs. public LVLMs (Prism, PathAlign, PathChat, Pathologyvlm) and vs. text-only / random-vision baselines. If code is unavailable, re-implement or cite the original reported numbers.

2) Data QC: describe the expert filtering process in the appendix and provide main results on the expert-approved subset. Consider releasing a public test leaderboard for future methods.

3) External validation: perform zero-shot tests on a small real-world clinical WSI set (anonymized) or community challenge data to show robustness to domain shift.

4) Explainability: include visualizations—heatmaps or patch-level attentions—for correct and incorrect cases to enhance interpretability.

Response: We thank the reviewer for the detailed suggestions. Points (1) Comparative experiments, (2) Data QC, and (3) External validation have been addressed in response to the corresponding comments above.

Regarding (4) Explainability, we agree that interpretability is an important consideration for slide-level QA. we provide qualitative interpretability by visualising two core components of ALPaCA-Hybrid: (i) GMM-based prototype maps, which

capture global, question-independent morphological organisation across magnification levels; and (ii) LongFormer-based attention maps, which reflect question-conditioned reweighting of visual evidence across scales.

Action:

- ➔ We added a new “Visualisation” section (Supplementary Note, **Page 3**) and included qualitative visualisations for four representative cases in **Extended Data Fig. 6** (Supplementary Note, **Page 29**).

Point-by-point responses

Reviewer #1 (Remarks to the Author):

I thank the authors for their detailed rebuttal and effort put into the revised manuscript.

Response: Thank you!

Major concerns

Comment 1: "General-Purpose" claims contradicted by external validation. The abstract and introduction continue to frame ALPACA as the "first general-purpose slide-level LVLM". However, the new external validation results contradict this broad claim. Specifically, performance on the external CUH-OV (ovarian) cohort dropped significantly to ~77% (compared to >90% on internal sets), which the authors attribute to "dataset-level mismatches" and a lack of exposure to "fine-grained subtype diversity". This indicates the model is brittle to distribution shifts and is not robustly "general-purpose." Authors should provide more convincing evidence for these broad claims, or they should be tempered in the Abstract and Introduction. The term "general-purpose" should be qualified to reflect the specific training distribution (TCGA/GTEx contexts). Furthermore, the Abstract should explicitly mention the performance degradation observed in external cohorts to provide a scientifically balanced view of the model's current capabilities.

Response: Thank you, we agree and we have followed all your suggestions.

“General-purpose” typically denotes unified whole-slide pathological reasoning across heterogeneous question categories and diagnostic contexts within a single framework; it does not necessarily imply complete invariance to all domain shifts.

It is however an important limitation to note and we have revised the Abstract, Introduction and Discussion to clarify the scope and explicitly report external validation performance, ensuring a balanced representation of the model’s current capabilities.

Action:

- ➔ We have mentioned the performance of the external cohorts in the **Abstract** (**Page 2**).

  "...exceeding 90% accuracy on internal close-ended benchmarks and maintaining robust performance on independent external cohorts (77–82%)."

- ➔ We have clarified the origin of the training data in the **Abstract (Page 2)** and further discussed the impact in the **Discussion** (**Page 14-15**).

  "... leveraging two large publicly available databases to extract 35,913 WSIs with curated descriptions alongside 341,051 question and answer pairs"

  "The training data are primarily derived from TCGA and GTEx cohorts, which cover a broad range of cancer types and tissue sites but may not fully reflect the diversity and heterogeneity encountered in real-world clinical settings."

- ➔ We have further qualified the term "general-purpose LVLM" by specifying that the model is designed for WSI question answering across a broad range of cancer types and tissue sites in the **Abstract** (Page 2) and **Introduction** (**Page 3**).

  "[...] question and answer pairs encompassing diverse diagnoses, procedures, cancer types and tissue sites."

  "To address this gap, we introduce ALPaCA (Adapting Llama for Pathology Context Analysis), a general-purpose LVLM designed for WSI question-answering (QA) across a broad range of cancer types and tissue sites"

Comment 2: Lack of downstream clinical task demonstration. The manuscript claims ALPACA is general purpose and enhances "diagnostic decision-making". However, the evaluation relies almost exclusively on aggregated QA correctness (e.g., multiple-choice accuracy). "Answering a question correctly" is often a proxy for clinical utility rather than a demonstration of it. To justify the "general-purpose" claim, the authors must demonstrate competence in standard pathological tasks using standard clinical metrics. Without clinical task validation the "general purpose" claim does not stand. Recommend authors to perform a retrospective analysis on the existing test sets (or external cohorts) to report standard metrics for at least one core pathological task beyond QA accuracy. For example, for the "Tumor Grade and Staging" category, provide a Confusion Matrix and Kappa score

comparing ALPACA's predicted grade vs. the ground truth. Or, for the "Tumor Detection" category, report Sensitivity and Specificity rather than just accuracy.

Response: Thank you, this is a good suggestion and we have performed the requested analyses.

To provide clinically interpretable evidence beyond aggregate QA accuracy, we added two retrospective downstream-task analyses and benchmarked ALPaCA against public LLM baselines (GPT-4o, Gemini, Qwen, MedGemma; SlideChat) on the test set of “General Slide-QA”.

For Tumor Detection, we report sensitivity, specificity, and accuracy, and we used all close-ended QAs (True/False and multiple-choice), yielding 239 evaluable samples. ALPaCA achieved Sensitivity = 0.889, Specificity = 1.000, and Accuracy = 0.996. The best-performing public LLM baseline reached Accuracy = 0.789 (Cluster-Gemini), with Sensitivity = 0.778 and Specificity = 0.789.

For Tumor Grade and Staging, we now report confusion matrices, Cohen’s kappa, and accuracy. Since kappa/confusion analysis requires order-consistent labels, we restricted this analysis to multiple-choice QAs with clinically orderable categories (low-to-high) and excluded non-orderable QAs. Concretely, this category contains 79 close-ended QAs in total; 49 were retained for ordinal evaluation (excluded: 26 yes/no QAs and 4 non-orderable QAs). Under this setting, ALPaCA outperformed all compared public LLM baselines in kappa (baseline range: 0.096–0.253; ALPaCA: 0.483).

Action:

- ➔ We added a new extended data figure (**Extended Data Figure 3**) to summarize performance in these two clinical question categories, including confusion matrices/kappa and sensitivity/specificity (**Supplementary Note, Page 26**).
- ➔ We have added these new analyses in the Results section (**Performance on close-ended questions, Page 7**). Detailed analysis definitions are provided in the figure caption of Extended Data Figure 3 (**Supplementary Note, Page 26**).

  “Beyond overall accuracy, we further evaluated clinically meaningful performance in two question categories, i.e., tumor grading and staging, and tumor detection, using confusion matrices, Cohen’s kappa, sensitivity, and specificity (**Extended Data Fig. 3**), where ALPaCA-Hybrid consistently outperformed competing LVLM baselines.”

Comment 3: There is an unexplained discrepancy in how the external cohorts were evaluated. The XJTU-RG cohort was evaluated on both close-ended and open-ended tasks , but the CUH-OV cohort appears to have been evaluated only on close-ended questions (79 pairs). Please provide open-ended evaluation results for the CUH-OV cohort or explicitly explain why this was omitted. Given the lower performance on close-ended questions for this cohort, omitting the open-ended results (the model's primary novelty) obscures potential failure modes.

Response: We thank the reviewer for raising this important point. The absence of open-ended evaluation on the CUH-OV cohort was not intended to omit results, but due to data governance constraints associated with this dataset. Construction of open-ended Slide-QA pairs requires LLM-assisted (GPT-based) processing of original pathology reports to generate structured slide-level summaries and candidate questions under a consistent question–answering schema (aligned with the General Slide-QA dataset), followed by manual review and refinement by board-certified pathologists.

However, the CUH-OV data usage agreement restricts external transmission of report content to third-party services, which precludes this LLM-assisted curation workflow for that cohort. In contrast, the XJTU-RG cohort does not impose such restrictions, allowing open-ended evaluation under the same schema.

Action:

➔ We have clarified this point and provided additional details on the open-ended QA generation process in the Methods section (**Curation of datasets, Evaluation datasets, Page 19**).

“(2) CUH-OV: … Open-ended evaluation was not conducted for this cohort due to data governance restrictions. For both external cohorts, close-ended questions were directly authored by pathologists based on slide-level assessment. Open-ended questions, where permitted by data governance, were derived from original pathology reports through an LLM-assisted summarization and question-generation process to ensure consistency with the “General Slide-QA” schema, followed by manual review and refinement by pathologists. All questions and answers were created and validated directly on the corresponding diagnostic slides to ensure accuracy and clinical validity.”

Comment 4: Specific Slide-QA (Morphology) is constructed using PathChat-generated patch descriptions that are summarized by GPT-4o into

slide-level reports, and these AI-generated reports are then used to curate QA pairs. This introduces a risk that the benchmark measures consistency with PathChat/GPT-4o-generated priors rather than independent, pathologist-verified ground truth. This is a significant limitation that should be addressed.

Response: We agree that benchmarks constructed using LLM-generated descriptions may introduce potential bias if not carefully validated. To address this concern, we conducted an additional pathologist review of the entire test set of "Specific Slide-QA (Morphology)".

For close-ended questions, the correctness of each labeled answer was reviewed by pathologists against the corresponding whole-slide image, and corrections were made when necessary. For open-ended questions, instead of modifying the reference answers, model predictions were directly evaluated by expert pathologists following the same expert-based assessment protocol used in the "General Slide-QA" benchmark.

Action:

- ➔ We have updated the reported close-ended performance results and incorporated expert pathologist assessment for the open-ended QAs for the "Specific Slide-QA (Morphology)'' dataset, in the Results section (**Accommodating in-depth disease-specific question answering, Page 13**) and the corresponding figure. (**Fig. 5, Page 43**).

  "In contrast, for the "Specific Slide-QA (Morphology)" dataset, open-ended responses were evaluated by pathologists rather than the GPT-4o-based judger to ensure that this LLM-assisted benchmark reflects independent, pathologist-verified judgment rather than consistency with LLM-generated priors. Among the 105 open-ended questions, ALPaCA-Hybrid was rated as TC (70) or PC (26) on 91.43% of questions, with only 8 IC responses."

- ➔ We have also provided additional details on the validation process for the close-ended QAs in the "Specific Slide-QA (Morphology)'' dataset (**Curation of datasets, Evaluation datasets, Page 18**)

  "Additionally, to ensure the reliability of the evaluation benchmark and mitigate potential inconsistencies introduced during the PathChat-assisted description generation process, we further reviewed the "Specific Slide-QA (Morphology)" test set with expert pathologists. For the close-ended Q&A pairs, pathologists reviewed all items against the corresponding whole-slide images. Of the 167 close-ended Q&A pairs, 140 were retained, including 5 with corrected answers, while 27 were removed because the questions were incorrect or

could not be reliably answered from the current slide. For the open-ended Q&A pairs, 111 questions were reviewed, and 6 incorrect questions were removed, leaving 105 valid items for expert scoring.”
